# Supplementary material for: Effects of mesotrione on the control efficiency and chlorophyll fluorescence parameters of Chenopodium album under simulated rainfall conditions
Source: PLoS One. 2022 Jun 3;17(6):e0267649. doi: 10.1371/journal.pone.0267649 (PMC9165882; doi:10.1371/journal.pone.0267649)
Supplement: Attachment — Submitted filename: PONE-D-21-13877_reviewer.pdf [file pone.0267649.s008.pdf]

PLOS ONE

Effects of mesotrione on the control efficiency and chlorophyll fluorescence parameters of Chenopodium under simulated rainfall condations

--Manuscript Draft--

**Manuscript Number:**	PONE-D-21-13877
**Article Type:**	Research Article
**Full Title:**	Effects of mesotrione on the control efficiency and chlorophyll fluorescence parameters of Chenopodium under simulated rainfall condations
**Short Title:**	Effects of mesotrione on Chenopodium under simulated rainfall condations
**Corresponding Author:**	Meijun Guo<br>Jinzhong University<br>Yuci, CHINA
**Keywords:**	Mesotrione; Chenopodium; Rainfall; Weed Control effect; Chlorophyll fluorescence
**Abstract:**	This experiment was conducted to study the effects of mesotrione on the control efficiency and chlorophyll fluorescence parameters of Chenopodium album . Simulating three rainfall intensities of 2 mm/h (light rain), 6 mm/h (moderate rain) and 10 mm/h (heavy rain) at different interval times (0.5 h, 1 h, 2 h, 4 h)to analyze variable regulation of the control effect, the photosynthetic pigment content and chlorophyll fluorescence parameters of Chenopodium after spraying mesotrione. The control efficiency of mesotrione on Chenopodium was decreased after rainfall, but the effect of different interval treatments on Chenopodium was different under different rainfall intensities. With the extension of rainfall time interval,The inhibition rate of plant height, plant control effect and fresh weight control effect of Chenopodium were gradually increased, the inhibition effect of rainfall on the efficacy was gradually decreased, at the same time, the contents of chlorophyll a, chlorophyll b, carotenoids, the maximum photochemical quantum efficiency (Fv/Fm), the actual photochemical quantum yield (Y(II)) and quantum yield(Y(NO)) production of regular energy consumption of Chenopodium were also increased, while the nonregulatory energy decreased gradually. The results showed that the contents of chlorophyll a and chlorophyll b in leaves of Chenopodium increased significantly by 35.63% and 35.38% compared with the control under the condition of simulating 6 mm/h in interval 1 hour. The study suggested that simulating 10 mm/h rainfall intensity had the greatest effect on Chenopodium , the photosynthetic pigment content, Fv/Fm and Y(II) of leaves were significantly higher than those in the control groups under 0.5 h, 1 h and 2 h interval treatments. The carotenoid content was the lowest and Y(NO) was the largest under the 4 h interval treatment. At this time, the control effect of mesotrione on Chenopodium was for maximum benefits. As is displayed that rainfall reduced the weeding control effect in the aspect of controlling Chenopodium with mesotrione is partly contributed to increase photosynthetic pigment content and enhance the PS II photochemical activity. In conclusion, the rain intensity of ≤2 mm/h did not affect the control effect of mesotrione on Chenopodium , and the rain intensity of 6 mm/h within 1 h and 10 mm/h within 2 h significantly reduced the efficacy of mesotrione.
**Order of Authors:**	Meijun Guo
	Mengmeng Sun
	Shuai Guo
	Yanfen Li
	Shuqi Dong
	Xie Song
	Xiangyang Yuan
**Additional Information:**	
**Question**	**Response**

Powered by Editorial Manager® and ProduXion Manager® from Aries Systems Corporation

**Financial Disclosure**<br><br>Enter a financial disclosure statement that describes the sources of funding for the work included in this submission. Review the submission guidelines for detailed requirements. View published research articles from *PLOS ONE* for specific examples.<br><br>This statement is required for submission and **will appear in the published article** if the submission is accepted. Please make sure it is accurate.<br><br>**Unfunded studies**<br>Enter: *The author(s) received no specific funding for this work.*<br><br>**Funded studies**<br>Enter a statement with the following details:<br>• Initials of the authors who received each award<br>• Grant numbers awarded to each author<br>• The full name of each funder<br>• URL of each funder website<br>• Did the sponsors or funders play any role in the study design, data collection and analysis, decision to publish, or preparation of the manuscript?<br>• **NO** - Include this sentence at the end of your statement: *The funders had no role in study design, data collection and analysis, decision to publish, or preparation of the manuscript.*<br>• **YES** - Specify the role(s) played.<br><br>* typeset	This paper was supported by the system of National Modern Agriculture Technology (CARS-06-13.5-A28), the program for the Top Young Innovative Talents of Shanxi Agricultural University (TYIT201406) and the Key research and development plan of Shanxi Province (201903D22103).
**Competing Interests**<br><br>Use the instructions below to enter a competing interest statement for this submission. On behalf of all authors, disclose any competing interests that could be perceived to bias this work—acknowledging all financial support and any other relevant financial or non-financial competing interests.<br><br>This statement is required for submission	The authors have declared that no competing interests exist.

and **will appear in the published article** if the submission is accepted. Please make sure it is accurate and that any funding sources listed in your Funding Information later in the submission form are also declared in your Financial Disclosure statement.<br><br>View published research articles from *PLOS ONE* for specific examples.<br><br>**NO authors have competing interests**<br><br>Enter: *The authors have declared that no competing interests exist.*<br><br>**Authors with competing interests**<br><br>Enter competing interest details beginning with this statement:<br><br>*I have read the journal's policy and the authors of this manuscript have the following competing interests: [insert competing interests here]*<br><br>* typeset	
**Ethics Statement**<br><br>Enter an ethics statement for this submission. This statement is required if the study involved:<br><br>• Human participants<br>• Human specimens or tissue<br>• Vertebrate animals or cephalopods<br>• Vertebrate embryos or tissues<br>• Field research<br><br>Write "N/A" if the submission does not require an ethics statement.<br><br>General guidance is provided below. Consult the submission guidelines for detailed instructions. **Make sure that all information entered here is included in the Methods section of the manuscript.**	No

**Format for specific study types**<br><br>**Human Subject Research (involving human participants and/or tissue)**<br>• Give the name of the institutional review board or ethics committee that approved the study<br>• Include the approval number and/or a statement indicating approval of this research<br>• Indicate the form of consent obtained (written/oral) or the reason that consent was not obtained (e.g. the data were analyzed anonymously)<br><br>**Animal Research (involving vertebrate animals, embryos or tissues)**<br>• Provide the name of the Institutional Animal Care and Use Committee (IACUC) or other relevant ethics board that reviewed the study protocol, and indicate whether they approved this research or granted a formal waiver of ethical approval<br>• Include an approval number if one was obtained<br>• If the study involved *non-human primates*, add *additional details* about animal welfare and steps taken to ameliorate suffering<br>• If anesthesia, euthanasia, or any kind of animal sacrifice is part of the study, include briefly which substances and/or methods were applied<br><br>**Field Research**<br><br>Include the following details if this study involves the collection of plant, animal, or other materials from a natural setting:<br>• Field permit number<br>• Name of the institution or relevant body that granted permission	
**Data Availability**<br><br>Authors are required to make all data underlying the findings described fully available, without restriction, and from the time of publication. PLOS allows rare exceptions to address legal and ethical concerns. See the PLOS Data Policy and FAQ for detailed information.	Yes - all data are fully available without restriction

A Data Availability Statement describing where the data can be found is required at submission. Your answers to this question constitute the Data Availability Statement and **will be published in the article**, if accepted.<br><br>**Important:** Stating 'data available on request from the author' is not sufficient. If your data are only available upon request, select 'No' for the first question and explain your exceptional situation in the text box.<br><br>Do the authors confirm that all data underlying the findings described in their manuscript are fully available without restriction?	
**Describe where the data may be found in full sentences. If you are copying our sample text, replace any instances of XXX with the appropriate details.**<br><br>• If the data are **held or will be held in a public repository**, include URLs, accession numbers or DOIs. If this information will only be available after acceptance, indicate this by ticking the box below. For example: *All XXX files are available from the XXX database (accession number(s) XXX, XXX.).*<br>• If the data are all contained **within the manuscript and/or Supporting Information files**, enter the following: *All relevant data are within the manuscript and its Supporting Information files.*<br>• If neither of these applies but you are able to provide **details of access elsewhere**, with or without limitations, please do so. For example:<br><br>*Data cannot be shared publicly because of [XXX]. Data are available from the XXX Institutional Data Access / Ethics Committee (contact via XXX) for researchers who meet the criteria for access to confidential data.*<br><br>*The data underlying the results presented in the study are available from (include the name of the third party*	All relevant data are within the manuscript and its Supporting Information files.

and contact information or URL).<br>• This text is appropriate if the data are owned by a third party and authors do not have permission to share the data.<br><br>* typeset	
Additional data availability information:	

Powered by Editorial Manager® and ProduXion Manager® from Aries Systems Corporation

Manuscript Click here to access/download;Manuscript;manuscript.docx

Effects of mesotrione on the control efficiency and chlorophyll fluorescence parameters of *Chenopodium* under simulated rainfall condations

Mengmeng Sun[1#], Meijun Guo[2#], Shuai Guo[1], Yanfen Li[1], Shuqi Dong[1], Sie Song[1], Xiangyang Yuan[1]*

1.Laboratory of Crop Chemical Regulation and Chemical Weed Control, College of Agronomy, Shanxi Agricultural University, Taigu, Shanxi, China; 2. Department of biological science and technology, Jinzhong University, Jinzhong, Shanxi, China

*Corresponding author's E-mail: yuanxianyang200@163.com

#These authors contributed equally to this work. SM and GM are co-first authors on this work.

Abstract: This experiment was conducted to study the effects of mesotrione on the control efficiency and chlorophyll fluorescence parameters of *Chenopodium album*. Simulating three rainfall intensities of 2 mm/h (light rain), 6 mm/h (moderate rain) and 10 mm/h (heavy rain) at different interval times (0.5 h, 1 h, 2 h, 4 h)to analyze variable regulation of the control effect, the photosynthetic pigment content and chlorophyll fluorescence parameters of *Chenopodium* after spraying mesotrione. The control efficiency of mesotrione on *Chenopodium* was decreased after rainfall, but the effect of different interval treatments on *Chenopodium* was different under different rainfall intensities. With the extension of rainfall time interval, The inhibition rate of plant height, plant control effect and fresh weight control effect of *Chenopodium* were gradually increased, the inhibition effect of rainfall on the efficacy was gradually decreased, at the same time, the contents of chlorophyll a, chlorophyll b, carotenoids, the maximum photochemical quantum efficiency (Fv/Fm), the actual photochemical quantum yield (Y(II)) and quantum yield(Y(NO)) production of regular energy consumption of *Chenopodium* were also increased, while the nonregulatory energy decreased gradually. The results showed that the contents of chlorophyll a and chlorophyll b in leaves of *Chenopodium* increased significantly by 35.63% and 35.38% compared with the control under the condition of simulating 6 mm/h in interval 1 hour. The study suggested that simulating 10 mm/h rainfall intensity had the greatest effect on *Chenopodium*, the photosynthetic pigment content, Fv/Fm and Y(II) of leaves were significantly higher than those in the control groups under 0.5 h, 1 h and 2 h interval treatments. The carotenoid content was the lowest and Y(NO) was the largest under the 4 h interval treatment. At this time, the control effect of mesotrione on *Chenopodium* was for maximum benefits. As is displayed that rainfall reduced the weeding control effect in the aspect of controlling *Chenopodium* with mesotrione is partly contributed to increase photosynthetic pigment

content and enhance the PS II photochemical activity.In conclusion, the rain intensity of ≤2 mm/h did not affect the control effect of mesotrione on *Chenopodium*, and the rain intensity of 6 mm/h within 1 h and 10 mm/h within 2 h significantly reduced the efficacy of mesotrione.

Keywords: Mesotrione; *Chenopodium*; Rainfall; Weed Control effect; Chlorophyll fluorescence

Introduction

As we all know, weed will reduce the yield and quality of crops, hinder mechanized sowing and harvesting, which slow down the pace of China's agricultural modernization [1-2]. *Chenopodium* is one of the common farmland malignant weed in northern China. It has strong environmental adaptability and a large root system. It has obvious advantages when competing with crops for water and fertilizer, led to a sharp decline in crop yield [3]. Mesotrione is a herbicide of broad-spectrum, selective stem and leaf before bud and after seedling mainly used to effectively control broad-leaved weeds and some gramineous weeds by inhibiting the catalytic factors of plant photosynthetic process. Mesotrione is widely used in weed control of maize and winter wheat due to its high activity, low residue, strong compatibility and safety to the environment and subsequent crops [4-5]. Summer is the key period for crop growth and weeding. However, local microclimate and severe convective weather occur frequently, and rainfall interfere the herbicide efficacy every year [6]. Therefore, it is attached importance to study the influence of rainfall on herbicides. There are great differences in the absorption rate and rain resistance among different stem and leaf treatment agents, and the interval time of affecting the efficacy is different, but most herbicides will not affect the weed when rainfall after 4-6 h [7]. Wu *et al*. [8] found that rainfall after the application had a significant inhibitory effect on the efficacy of nicosulfuron methyl; paraquat could effectively control *Alopecurus aequalis* after 0.5 h and 1 h interval treatments; there was no significant difference in bensulfuron efficacy between without rainfall and after 2 h rainfall [10]; Wang *et al*. [11] showed that the effect of imazethapyr was no longer affected under the 9 mm/h rainfall intensity. When the interval time of rainfall is 0.5 h, the effect of rainfall on barnyardgrass was significantly reduced, it's down to 19.42% [12]. At present, it is known that mesotrione has effects on the growth of weeds and crops, such as reducing the plant height of maize, making *Amaranthus retroflexus* heart leaves be yellow, reducing the photosynthetic pigment content, reducing the maximum photochemical quantum efficiency (Fv/Fm) and the actual photochemical quantum yield Y(II) [13-15]. The inhibition rate of plant height, plant control effect and fresh weight control effect can well reflect the control effect of herbicides on weeds; photosynthetic pigment content reflects the green extent of plants; chlorophyll fluorescence parameters as a probe of photosynthetic capacity of leaves can reflect the status of weeds under herbicide stress [16]. Most

of the studies on mesotrione focused on its control effect and crop safety. There were no reports on the control effect and chlorophyll fluorescence parameters of mesotrione on broad-leaved weed *Chenopodium* under rainfall conditions. In this experiment, artificial rainfall was used to simulate rainfall, and *Chenopodium* is a common malignant weed in the field, was used to simulate different rain intensities at different intervals after spraying mesotrione.

In order to assess the possibility of mesotrione application and understand the related mechanism, we investigated the weed control, agronomic traits, photosynthetic pigment content and the chlorophyll fluorescence parameters of *Chenopodium* to mesotrione in this current study.

1 Material and methods

1.1 Experimental site

Field experiments were conducted at farming station of Shanxi Agricultural University during July to August 2019，Jinzhong, China (37.43°N, 112.61°E). The area is located in the northeast of Jinzhong region and belongs to a warm temperate continental monsoon climatic with an altitude of approximately 800 meters, the annual average temperature was 9.9 ℃, and the frost-free period was about 159 days. Annual precipitation of the province was 400-650 mm, but the seasonal distribution was uneven, with more than 60% of precipitation concentrated in June to August [17].

1.2 Experimental design

Four rainfall intervals were set: 0.5 h (T1), 1 h (T2), 2 h (T3), 4 h (T4), and no rainfall after mesotrione application was taken as control (CK). The completely randomized design was conducted by the field experiment with three replicates in 4 m^2 (2 m × 2 m) .The recommended dose (200 ml/667 m^2) of mesotrione (Shandong Shengbanglvye Chemical Limited Company) was sprayed by sprayer.

1.3 Rainfall designs

According to the standard of China Meteorological Administration(CMA): the hourly rainfall is 0.1～2.5 mm/h for light rain, 2.6～8 mm for moderate rain, and 8.1～15.9 mm for heavy rain. Simulating light rain 2 mm/h, moderate rain 6 mm/h and heavy rain 10 mm/h by electric sprayers (Lufeng, 3 WBD-20 C, 20 L). Artificial spraying was carried out by simulating natural rainfall, According to the calculation method that 1 L of rainfall per square meter is 1 mm [18], by adjusting the knob on the edge of the electric sprayer, 4 L, 12 L, and 20 L of water are sprayed out within half an hour to obtain 2 mm/h three rain intensities of 6 mm/h and 10 mm/h. Before the expertiment,

several tests were carried out with the meteorological rain gauge, and the positions of different rain intensities on the sprayer knob were accurately marked. The simulated rainfall is carried out under calm or breezy weather to reduce the error, and the test rain intensity is determined again every time the simulated rainfall is performed, and the error of these results is within 0.2 mm/h.

1.4 Data collections

1.4.1 Determination of weed control effect

To calculate the reductions in weed number and biomass, the shoots of all weed plants were cut from three 0.25 m^2 (0.[illegible]×0.5 m) quadrats in each plot at 15 and 30 d after treatment (DAT) [19]. When mesotrione in soil was analyzed, *Chenopodium* were collected carefully by taking out from the soil. The plant was weighed after washing, the biomass of each weed sample were measured. The efficacy and reduction was calculated by this equation:

$$Efficacy\ (\%) = [(C - B)/C] \times 100 \qquad (1)$$

where C is the aboveground height of the nontreated control plot and B is the height of an treated plot.

$$Reduction\ (\%) = [(SCP - TP)/SCP] \times 100 \qquad (2)$$

Where TP is the weed plant number or biomass in the treated plots and SCP is the sum of the plant number or biomass in the weedy controls.

1.4.2 Determination of photosynthetic pigment content

Photosynthetic data was collected after 3, 6 and 9 d DAT, weigh 0.05 g of mature and intact leaves with the same growth in the middle and upper part of the plant, cut them into pieces, and put them into a stoppered test tube containing 5 ml of 96% ethanol, and soak in a dark place at room temperature for 48 hours until the leaves are milky white and oscillate for several hours during the extraction process. The absorbance of the extract is measured at wavelengths of 470 nm, 649 nm and 665 nm, and the content of chlorophyll a, chlorophyll b, and carotenoid are calculated [20]. The calculation formulas are in the following:

$$\mathrm{Ca} = 13.95A_{665} - 6.88\,A_{649}$$

$$\mathrm{Cb} = 24.96\,A_{649} - 7.32A_{665}$$

$$\mathrm{Cx.c} = (1000\,A_{470} - 2.05\,\mathrm{Ca} - 114.8\,\mathrm{Cb})/245$$

1.4.3 Determination of chlorophyll fluorescence parameters

The chlorophyll fluorescence parameters were tested at 12 h, 24 h, 2 d and 4 d after application respectively. The middle and upper parts of the plant were selected to be fully stretched and mature leaves. The MINI-PAM-Ⅱ portable pulse modulated chlorophyll fluorometer (Walz, Germany), first use the leaf clip of the fluorometer to clamp the *Chenopodium* for 30 min. Actual photochemical efficiency Y(II), maximum photochemical quantum yield Fv/Fm of leaves, regulated energy dissipation quantum yield Y(NPQ) and non-regulated energy dissipation quantum yield Y(NO) were examined.[21].

1.5 Statistical analyses

DPS 6.5 Analysis System was used to conduct all statistical analyses. Data were presented as mean ± standard error of the mean. Duncan's test was used to analyze significant differences among treatments in growth parameters, chlorophyll content and fluorescence parameters.

2 Results and Discussions

2.1 The weed effect of simulated rainfall on mesotrione

After spraying mesotrione, *Chenopodium* exhibits disease symptoms such as chlorosis and albino of stems and leaves. With applicating the herbicide, the rainfall causes the plant height inhibition rate to decrease, and enhanced gradually increases with the extension of the interval. It is represented in (Table 2) that when the rain intensity is 2 mm/h, at 15 DAT, compared with CK, T1,T2 and T3 cause a significant reductions in the inhibition rate of plant height(11.70%, respectively). At 30DAT, there were no significant difference between treatments and CK. When the rain intensity was 6 mm/h, after 30 days, the inhibition rate of the treatment with only T1 interval significantly decreased by 5.57% compared with CK, while the other treatments had no significant difference compared with CK. In addition to, with the increase of rainfall intensity, there was significantly reduced by 7.53% and 4.91% at T1 and T2 in the inhibition rate of plant height in10 mm/h.

The control effect and fresh weight control effect of the plants are belong to the weed control effects, which is increased significantly at 30 DAT compared with 15 DAT. At 15 DAT, the weed control effects of only T1 and T2 were significantly lower than that of CK by 15.06%, 11.83%, 13.31%, and 11.38% in 2 mm/h; the rain intensity was at 6 mm/h and 10 mm/h, the weed control effects were significantly lower than CK. At 30 DAT, when the rain intensity was 2 mm/h, the weed control effects were still more than 90% at all treatments. However, the fresh weight control effect of T1 and T2 treatments is significantly lower than CK by 16.34% and 6.90% in 6 mm/h; compared

with CK, T1, T2 and T3 were reduced in the fresh weight control (19.30%, 8.43%, 6.80% respectively) when the rain intensity is 10 mm/h.

2.2 Effects of simulated rainfall on photosynthetic pigment content of *Chenopodium* leaves after spraying mesotrione

2.2.1 The effect of simulated rainfall on the carotenoid content of *Chenopodium* after spraying mesotrione

The data in Figure 1 showed that the corresponding rainfall intensity was 2 mm/h. Compared with CK at 3 DAT, T1 and T2 treatments show a significant increase, the increase have been 24.73% and 17.2%. Compared with all interval treatments, CK at 6 DAT did not see a big difference, which could indicate that the rainfall in this period is not capable of preventing the albino process of *Chenopodium*.

When the rainfall intensity reached 6 mm/h, it could find that the corresponding carotenoid content of T1 and T2 treatments increased at 3 and 6 DAT, and the ranges were 21.85%, 13.89%, 20.00% and 21.91%, respectively. Except that the T4 interval treatment was a bit different, the carotenoid content involved in other treatments looked higher than the content contained in CK. And the growth rates are 39.78%, 36.56%, 29.03% and 33.32%, 19.32%, 15.79%.

2.2.2 The effect of simulated rainfall on the chlorophyll content of *Chenopodium* after spraying mesotrione

Like the carotenoid content, the content of chlorophyll a and chlorophyll b decreased gradually and showed a decreasing trend with the extension of rainfall interval. As shown in Table 2, when the rain intensity was 2 mm/h, the chlorophyll a and chlorophyll b contents of all interval treatments were not significantly different than CK at 6 DAT. At the same time, chlorophyll a+b content under T1 treatment has an increase of 27.86% compared with CK, the difference was not very significant between the other treatments and CK.

Under the situation of 3 DAT, the rainfall intensity reached 6 mm/h. In this case, in addition to the different results produced by T4 processing, the chlorophyll a content corresponding to other treatments has a more significant increment compared to CK,; In addition, when the T1 and T2 intervals were used for treatment, the chlorophyll b content would increase significantly, with an increase of 46.41% and 35.38%. On the other hand, when DAT was 6 DAT, all treatments at certain intervals show that the contents of chlorophyll a and chlorophyll b were much higher than that situation of CK. It could be claimed that compared with carotenoids, 6 mm/h rainfall had a greater

impact on chlorophyll a and b. Although the interval is only 4 hours, it has a higher content than CK. When the rainfall intensity under 3 DAT reached 10 mm/h, the chlorophyll a content corresponding to all treatment intervals was at a high level, exceeding the chlorophyll content of CK. In the meantime, there was no significant difference between T1, T2 and T3 treatment intervals.

2.3 Effects of simulated rainfall on chlorophyll fluorescence parameters of *Chenopodium* after spraying mesotrione

2.3.1 The effect of 2 mm/h rain intensities on chlorophyll fluorescence parameters after spraying mesotrione

These two types of indicators Fv/Fm and Y(II) in the leaves showed a certain decline after using mesotrione. At the same time, all processing intervals were showing an upward trend. However, if the time between rains continued to extend, there was a possibility that there might be an overall decline between the two (Figure 2). The max value is T1 after the mesotrione, and the minimum will be T4. After taking relevant treatments, there is no significant difference in Fv/Fm indicators of other treatments compared with CK. But this does not include T1 treatment, T1 treatment caused a significant increase in Fv/Fm, with an amplitude of 27.65%;

In addition, it can be seen that there is no big difference between T4 interval treatment Y(II) and CK, and the remaining treatment Y(II) has a greatly increasment , whose amplitude are 43.85%, 36.97%, and 35.60% respectively. On the 4th day, if T1 treatment is not considered, the Fv/Fm of other treatments all show a certain increase. Compared with CK, the corresponding incidence rates are 66.54%, 46.62% and 23.42%, respectively; In the figure, the Y(II) treatment at T1 and T2 interval both increased to a certain extent, which increased by 210.88% and 127.98% respectively (Figure 2.A-B). In addition, after the use of mesotrione, the entire graph trend showed that Y (NPQ) would decrease with the extension of the rain interval, but the value of Y (NO) would continue to increase. If T1 was not considered at 4 DAT, the Y (NPQ) of T2 interval treatment would increase significantly, and there might not be much difference between other treatments and CK Y (NPQ); in addition, the Y (NO) index after T1 interval treatment had a certain drop of 10.38%. In contrast, there were not many differences between other treatments and CK (Figure 2.C-D).

2.3.2 Effect of 6 mm/h rain intensities on chlorophyll fluorescence parameters after spraying mesotrione

Based on the situation shown in Figure 2, if the rain interval continued to extend, the indicators Fv / Fm and Y(II) would both show a downward trend. In particular, if the application exceeds 12

hours, the effect of mesotrione will be greatly reduced due to rainfall. In addition, the Fv/Fm index values are much higher than in the CK case in all treatment intervals, and the corresponding growth rates are 42.47%, 33.27%, 24.97% and 17.96%, respectively. In addition, on the 4th day, the condition of the patients treated with the T4 interval will be relieved, and the Fv/Fm index will no longer be significantly different from that in the case of CK. It should also be noted that compared with the case of CK, the Fv/Fm of other treatments will be greatly improved, and the corresponding increases are 82.59%, 72.07% and 62.67% respectively (Figure 3.A).

Compared to the situation of CK, Y(II) of *Chenopodium* had a increasment by 528.50% and 398.96% at 4 DAT for T1, and T2 treatments, And it is noted that it is almost the same as T3, T4 treatments and CK (Figure 3. B), stating that if the coppresponding rain intensity reached 6 mm/h, the rainfall within 1 hour could be give a great influence at the efficacy of mesotrione.

The Y(NPQ) value of *Chenopodium* was measured at different periods, and its magnitude was as follows: 24 hours after treatment>12 hours after treatment>2 d after treatment>4 d after treatment. The photosynthetic inhibitory effect of *Chenopodium* was the strongest at 4 DAT. Compared with 12 hours after application, because of the self-protection mechanism of *Chenopodium*, Y(NPQ) will has a slower increasment, and then slowly decreased; Y(NO) showed that with the extension of the interval and the passage of the reaction time gradually increase. Compared with CK at 24 hours after the mesotrione, Y(NPQ) in all interval treatments decreased significantly; Y(NO) in all interval treatments was significantly lower than CK, T1 and T2 treatments would not show much differences.(Figure 3.C-D)).

2.3.3 The effect of 10 mm/h rain intensities on chlorophyll fluorescence parameters after spraying mesotrione

In addition, based on the display status of the graph, rainfall will increase the Fv/Fm and Y(II) values of *Chenopodium* (Figure 3). However, this situation will be weakened by the extension of the rainfall time, and this trend will gradually flatten out. Compared with the results under CK conditions, Fv/Fm show the great increment by 25.14%~43.93% for all interval treatments at 12 h and 4 days after treatment application. Ignoring the results from T4 treatment, others would demonstrate the better increment by 85.08% and 78.31%, 71.54%, which was much higher than CK, and T1, T2 and T3 treatment, Y(II) give a good growth from the observation, which means that the possible influence of mesotrione on *Chenopodium* become much weaker when it is raining. If the rain intensity arrives at 10 mm/h, T1, T2 and T3 treatment had a much stronger restrictive effect mesotrione (Figure 4.A-B). Y(NPQ) will fluctuate when it suffers from rain intensity of 2 mm/h and

6 mm/h. It might have a growth at 24 h after treatment compared with 12 h, and then slowly declined and minimized after 4 d, except for the T4 treatment. T1, T2 and T3 treatments is possible to have an impact on the mesotrione of *Chenopodium*. Compared with CK, Y(NPQ) gives a higher increment at 422.82%, 220.58%, and 199.11% (Figure 4.C).

3 Discussion

With the continuous acceleration of the process of agricultural modernization and the gradual transfer of rural labor to cities, chemical control has become the dominant method in the field of weed control due to its high efficiency and practicality [22-23]. However, as a means of intervention in production, herbicides put forward many requirements on natural conditions in practical applications. Temperature, humidity, light, wind, and precipitation will affect the effectiveness of the mesotrione [24-25]. Mesotrione is commonly used as a stem and leaf treatment agent to control broadleaf weeds in corn fields. The scouring effect of rain water reduces the effective components of the medicament attached to the leaf surface and reduces the herbicide effect [26].

Zhang *et al*.[27] reported that with the increase of the dose of mesotrione, the inhibitory effect on corn growth increased, mainly manifested as a significant decrease in plant height; Zhang *et al*. [15] and Wang *et al*. [16] reported spraying nitrate. After sulcotrione, the heart leaves of Amaranthus retroflexus were severely chlorosis and then the whole plant will died. The control efficiency of plant and fresh weight were high than common plants; Gao *et al*. [28] reported that the effect of mesotrione in the control of weeds increased with weed physiological metabolism intensified, the symptoms are more significant later. In this study, on condition that the rain intensity was 2 mm/h and the rainfall interval was T1, the plant height inhibition rate of *Chenopodium* was still close to that of no rainfall after application, indicating that a light rain after application would not affect the efficacy of mesotrione rain intensity is 6 mm/h. Under the treatment of 2 h interval, with the extension of the rain interval time, the control effect of mesotrione on *Chenopodium* gradually increases, and the control effect of plant and fresh weight is still above 90%. The efficacy of mesotrione was not significantly affected.

The mechanism of action of herbicides is mainly to cause weed death by interfering or inhibiting the physiological metabolism of plants, including photosynthesis, fatty acid synthesis, pigment synthesis, amino acid metabolism [29]. HPPD is the only target enzyme for mesotrione plants, and carotenoids are synthesized products of the target enzyme (HPPD) after the catalytic reaction. Therefore, the changes in the mass fraction of photosynthetic pigments in the leaves can

reflect the HPPD enzyme in *Chenopodium*. The level of activity is used as an important indicator of the effectiveness of mesotrione on weed control [30]. Zhang *et al*. [31] found that the carotenoid, chlorophyll a and chlorophyll b content in the leaves of *Chenopodium* gradually decreased with the time of the herbicide reaction after spraying mesotrione. Zhang *et al.* [32] thinks that the erosion resistance is better with the increase of retention on leaves. In this experiment, the control effect decreased after spraying mesotrione, may be due to rainfall washing the pesticide retention on the plant surface, which increased the photosynthetic pigment content of *Chenopodium* leaves, but as the reaction time increased, the content of photosynthetic pigments decreased step by step. However, when the rainfall intensity increased, the content of carotenoids in leaves of *Chenopodium* was significantly higher than that of the control when the rainfall intensity was 6 mm/h and the interval was within 1 h. If the rainfall intensity continued to add to 10 mm/h, the carotenoid content of leaves of *Chenopodium* was still significantly increased after 2 h interval times.

Chlorophyll fluorescence is a means of rapidly identifying injury to leaves in the absence of visible symptoms. This is a detailed analysis of causes of change in photosynthetic capacity. It has been widely used in laboratory studies in understanding the mechanism of photosynthesis and the mechanisms by which a range of external factors alter photosynthetic capacity. The measurement of chlorophyll fluorescence also has considerable potential for use in the field situation [33]. Fv/Fm represents the strength of photoinhibition after the plant is stressed, reflecting the maximum light energy conversion efficiency in the photosynthetic reaction center, and Y(II) refers to the actual light energy conversion efficiency of the plant photosynthesis process [34]. Y(NPQ) is one of the self-protection indicators for plants to convert excess light energy into heat and the degree of light damage to plants is represented by Y(NO) [35-36]. Yang *et al*. [37] found that 2,4-D treatment significantly reduced the Fv/Fm and Y(II) of Jinfen No.107 and Jingu No.29 millet seedling leaves. Guo *et al*. [38] reported that after spraying chipton, the Fv/Fm of millet has an increasing inhibitory effect as the increased of the spraying concentration. We found that Fv/Fm, Y(II), Y(NPQ) of *Chenopodium* leaf gradually decreased with the extension of the interval, Y(NO) showed an upward trend conversely. We had tested the Y(NPQ) of the *Chenopodium* leaf after spraying mesotrione for 24 h, it was higher than that of 12 h, indicating that the *Chenopodium* can relieve the caused by mesotrione to a certain extent by increasing heat dissipation after being slightly stressed. Photodamage, the photochemical reaction efficiency continues to decline due to the serious herbicide stress, and it shows a downward trend as the rain interval increases. Therefore, the efficacy of mesotrione has weakened, which is mainly due to the increase in leaf photosynthetic pigment content, and the increase of photosynthetic capacity of photosystem II, which affects the

control effect of mesotrione and the growth of *Chenopodium* and photosynthesis.

4 Conclusion

In conclusion, rainfall reduced the control effect of mesotrione on *Chenopodium*, one of the reasons was the increase of photosynthetic pigment content and the enhancement of photosystem II activity. The 2 mm/h raininess not significantly effected mesotrione the control effect of mesotrione; In the circumstance of 6mm/h of the rainfall intensity, the control effect of mesotrione was significantly reduced within 0.5 h and 1 h; and in the 10 mm/h of the rainfall intensity, the effect of mesotrione was significantly reduced by rainfall within 2 h.

Acknowledgments

We would express sincere appreciation to two anonymous reviewers for their thoughtful and valuable comments, which helped us to improve this manuscript. Thanks are also due to Professor X.Y. Yuan of Shanxi Agricultural University for suggestions on the manuscript. This paper was supported by the system of National Modern Agriculture Technology (CARS-06-13.5-A28), the program for the Top Young Innovative Talents of Shanxi Agricultural University (TYIT201406) and the Key research and development plan of Shanxi Province (201903D22103)..

Authors' contribution

The author would like to thank all authors for their help in the experience and manuscript. Mengmeng Sun was responsible for preparing the final version of manuscript. Xiangyang Yuan helped conceive the experiments and was the project director. Meijun Guo and Shuqi Dong were involved in review the language of the manuscript. Xie Song and helped analysis and interpretation of data. Shuai Guo and Yanfen Li were involved in literature collection.

Figure1: Effect of simulated rainfall on carotenoid content in leaves of *Chenopodium* treated by mesotrione after 3(A) and 6(B) DAT. X-axis represents different rainfall intensity; y-axis represents the carotenoid content of leaves. CK means that there was no rain after the application of mesotrione, T1, T2, T3, and T4 represent different rainfall interval times (0.5 h, 1 h, 2 h, and 4 h). Effect of simulated rainfall on carotenoid content in leaves of Chenopodium treated by mesotrione after 3 DAT (A) and 6 DAT (B). Lowercase letters in the same column indicate significant differences at the 0.05 level.

Figure2: Effect of 2 mm/h rain intensity on chlorophyll fluorescence parameters of

***Chenopodium* treated by mesotrione.** CK and T1, T2, T3, T4 on the x-axis represent no rain and different rainfall interval times (0.5 h, 1 h, 2 h, and 4 h) respectively; y-axis represents chlorophyll fluorescence parameters. Diamonds, squares, triangles and circles represent different times after treatment (12 h, 24 h, 2 d and 4 d, respectively). A)the maximum photochemical quantum efficiency (Fv/Fm); B)the actual photochemical quantum yield Y(II); C)the regulated energy dissipation quantum yield Y(NPQ); D)the non-regulated energy dissipation quantum yield Y(NO) of Chenopodium treated by mesotrione. Lowercase letters in the same column indicate significant differences at the 0.05 level.

Figure3: Effect of 6 mm/h rain intensity on chlorophyll fluorescence parameters of *Chenopodium* treated by mesotrione. CK and T1, T2, T3, T4 on the x-axis represent no rain and different rainfall interval times (0.5 h, 1 h, 2 h, and 4 h) respectively; y-axis represents chlorophyll fluorescence parameters. Diamonds, squares, triangles and circles represent different times after treatment (12 h, 24 h, 2 d and 4 d, respectively). A)the maximum photochemical quantum efficiency (Fv/Fm); B)the actual photochemical quantum yield Y(II); C)the regulated energy dissipation quantum yield Y(NPQ); D)the non-regulated energy dissipation quantum yield Y(NO) of Chenopodium treated by mesotrione. Lowercase letters in the same column indicate significant differences at the 0.05 level.

Figure4: Effect of 10 mm/h rain intensity on chlorophyll fluorescence parameters of *Chenopodium* treated by mesotrione. CK and T1, T2, T3, T4 on the x-axis represent no rain and different rainfall interval times (0.5 h, 1 h, 2 h, and 4 h) respectively; y-axis represents chlorophyll fluorescence parameters. Diamonds, squares, triangles and circles represent different times after treatment (12 h, 24 h, 2 d and 4 d, respectively). A)the maximum photochemical quantum efficiency (Fv/Fm); B)the actual photochemical quantum yield Y(II); C)the regulated energy dissipation quantum yield Y(NPQ); D)the non-regulated energy dissipation quantum yield Y(NO) of Chenopodium treated by mesotrione. Lowercase letters in the same column indicate significant differences at the 0.05 level.

References

[1] DAS T K, YADURAJU N T. The effects of combining modified sowing methods with herbicide mixtures on weed interference in wheat crops. International Journal of Pest Management, 2012, 58(4): 310-319.

[2] LI X J. Main problems and management strategies of weeds in agricultural fields in China in recent years. Plant Protection, 2018, 44(5): 77-84. (in Chinese)

[3] SINGH V, GUPTA S, SINGH H, RAGHUBANSHI AS. Ecophysiological characteristics of five weeds and a wheat crop in the Indo-Gangetic Plains, India. Weed Biology and Management, 2015, 15(3):102-112.

[4] YANG H H, WANG L Q, XIE Z J, YU Q, LIU G, TAN H R. The tyrosine degradation gene hppD is transcriptionally activated by HpdA and repressed by HpdR in Streptomyces coelicolor, while hpdA is negatively autoregulated and repressed by HpdR. Molecular Microbiology, 2007, 65(4): 1064-1077.

[5] GUO W L, FENG L, CUI Y, WU D D, ZHANG T J, ZHANG C, TIAN X S. Weed control effect of 30% nicosulfuro•atrazine•mesotrione oil dispersion and the safety to sweet corn in field. Journal of Maize Sciences, 2020, 28(4): 178-183. (in Chinese)

[6] CHEN H Y，DENG G Q, FU H Y, SHE Y B. Advances in detection of systemic pesticide residues in agricultural products. Chemical Reagents, 2020, 42(10): 1139-1147. (in Chinese)

[7] TAO B. Practical Techniques for Chemical Control of Weeds. Beijing: Chemical Industry Press, 2013: 255.

[8] Wu J Y, S.Mathiassen, P Kudsk. The influence of weed species, leaf stage and rainfastness on the performance of nicosulfuron. Chinese Journal of Pesticide Science, 2003(1): 77-81. (in Chinese)

[9] LUO Z R, FU Q, XIE Z B. Effect of simulated rainfall on the weeding effect of paraquat. Journal of Fruit Science, 1995(2): 104-106. (in Chinese)

[10] WANG C F, YOU Y, WUANG J,WU J Y, CHEN F,WANG Q H. Effect of turf-mowing and simulated rainfall on the efficiency of bensulfuron-methyl•metsulfuron-methyl. Journal of Fujian Agriculture and Forestry UniversitY(Natural Science Edition), 2009, 38(4): 351-355. (in Chinese)

[11] WANG Q H, WU J Y, JIAGN GQ. Effects of rainfall and adjuvants on phytotoxicity of imazethapyr. Chinese Journal of Pesticide Science, 2003(3): 55-58. (in Chinese)

[12] GAO S, ZHANG Z J, AN W L, LIN C F, YANG Y T, MA H J, LI M. Biological activity and safety of herbicide mesotrione in corn field. Agrochemicals, 2004(10): 469-471. (in Chinese)

[13] ZHANG Y, LV A Q, WU C X, LU X T, MA C. Controlling weeds with mesotrione 15% SC in bluegrass lawn. Journal of Weed Science, 2013, 31(4): 54-56. (in Chinese)

[14] WANG Z, YAO A Q. Mesotrione bioactivity and selectivity to corn. Journal of Weed Science, 2014, 32(2): 59-61. (in Chinese)

[15] Vranjes F, Vrbnicanin S , Nedeljkovic D , *et al*. The response of Chenopodium album L. and Abutilon theophrasti Medik. to reduced doses of mesotrione[J]. Journal of Environmental ence and Health Part B, 2019, 54(7):1-7.

[16] Dayan FE , Zaccaro M D M. Chlorophyll fluorescence as a marker for herbicide mechanisms of action[J]. Pesticide Biochemistry & Physiology, 2012, 102(3):189-197.

[17] HAO Z C, WANG H, DUNZHU J C, JU Q. Spatiotemporal variations of soil moisture across Shanxi province during 2003-2012, Acta Scientiarum Naturalium Universitatis Sunyatseni, 2014, 53(5):134-141. (in Chinese)

[18] LI D-K, ZOU Y X, MA X Z, ZHANG F Z, DONG P, LA Y P. Field efficacy and rain resistance of fungicides against potato late blight. China Plant Protection, 2012, 32(5): 50-52+59. (in Chinese)

[19] CUI H L, JIA F, QUAN Z H, CUI H L, LI X J. Effects of glyphosate on weed control, soybean safety and weed occurrence in transgenic herbicide-resistant soybean. Scientia Agricultura Sinica. 2020, 53(6): 1166-1177. (in Chinese)

[20] GAO J F. Plant Physiology Experiment Guide. Beijing: Higher Education Press, 2006.

[21] FORCE L, CRITCHLEY C, RENSEN J J S. New fluorescence parameters for monitoring photosynthesis in plants. Photosynthesis Research, 2003, 78(1): 17-33.

[22] PETERSON M A, COLLAVO A, OVEJERO R, SHIVRAIN V W, MICHAEL J. The challenge of herbicide resistance around the world: a current summary. Pest Management Science, 2018, 74(10):2246-2259.

[23] ZHAO Y N, ZI X J, WANG W, FENG Y, YANG P, WANG P K, GAO X L. Control effects of different herbicides on weeds as well as their effects on growth and development of broomcorn millet. Chinese Journal of Applied Ecology, 2020, 31(7): 2415-2421. (in Chinese)

[24] PRASHANT J, VIPAN K, JOSEFINA G, NICHOLAS R. Tank mixing pendimethalin with pyroxasulfone and chloroacetamide herbicides enhances in-season residual weed control in

corn. Weed Technology, 2015, 29(2):198-206.

[25] BRADLEY C J, BRYAN GY. Influence of temperature and relative humidity on the foliar activity of mesotrione. Weed Science, 2002, 50(2):157-161.

[26] HAWKES T R, LANGFORD M P, VINER R, BLAIN R E, CALLAGHAN F M, MACKAY E A, HOGG BRIDGET V, SINGH S, DALE R P. Characterization of 4-hydroxyphenylpyruvate dioxygenases, inhibition by herbicides and engineering for herbicide tolerance in crops. Pesticide Biochemistry and Physiology, 2019, 156: 9-28.

[27] ZHANG H-J, LIU X, NI H-W, LI X-J, CUI H L. Early diagnosis and mitigation methods of mesotrione on maize. Journal of Weed Science, 2009(4): 19-22. (in Chinese)

[28] GAO X X, LI M, FANG F, YU T C, LIU X T. Interaction of clopyralid and mesotrione in maize field. Journal of Maize Sciences, 2015, 23(3): 143-148. (in Chinese)

[29] GUO LEI, ZHANG B B, SHEN J H, HE X, SONG H F. Effects of glyphosate and paraquat on root morphology and aboveground growth of prunus persica seedlings. Chinese Journal of Applied Ecology, 2020, 31(2): 524-532. (in Chinese)

[30] MAMIKO S, TAKAHIRO H, YOSHIO S, HIRASE K, BANBA S. Action mechanism of bleaching herbicide cyclopyrimorate, a novel homogentisate solanesyltransferase inhibitor. Journal of Pesticide Science. 2018, 43(4):233-239.

[31] FENGWEN ZHANG, XIANGFENG YAO, SHIANG SUN, LIPENG WANG, WEITANG LIU, XINGYIN JIANG, JINXIN WANG. Effects of mesotrione on oxidative stress, subcellular structure, and membrane integrity in Chlorella vulgaris. Chemosphere, 2020(247), 125668.

[32] ZHANG P , JIANG X Y, TAN H L,SHANG D L, LI X D, ZHANG J W. Effects of four spray adjuvants on the rainfastness of azoxystrobin on maize leaf surfaces and their effect on maize safety. Chinese Journal of Pesticide Science, 2018, 20(2): 239-248. (in Chinese)

[33] SJOLLEMA S B, BEUSEKOM S A, GEEST H G, BOOIJ P, ZWART D, VETHAAK A D, ADMIRAAL W. Laboratory algal bioassays using PAM fluorometry: Effects of test conditions on the determination of herbicide and field sample toxicity. Environmental Toxicology and Chemistry, 2014, 33: 1017-1022.

[34] SHAO G Q, LI Z J, NING T Y, ZHENG Y H. Responses of photo-synthesis, chlorophyll fluorescence, and grain yield of maize to controlled release urea and irrigation after anthesis. Journal of Plant Nutrition and Soil Science, 2013, 176: 595-602.

[35] TSAI Y C , CHEN K C, CHENG T S, LEE C, LIN S H, TUNG C W. Chlorophyll fluorescence analysis in diverse rice varieties reveals the positive correlation between the seedlings salt tolerance and photosynthetic efficiency. BMC Plant Biology, 2019, 19(1):403.

[36] YANG H J, YUAN X Y, GUO P Y, DONG S Q, ZHANG L G, WEN Y Y, SONG X E, WANG H F. Effects of brassinolide on photosynthesis, chlorophyll fluorescence characteristics and carbohydrates metabolism in leaves of foxtail millet under Sigma broad stress. Scientia Agricultura Sinica, 2017, 50(13): 2508-2518. (in Chinese)

[37] YANG X F, SUN P, SUN D S, SONG X E, DONG S Q, YUAN X Y, GUO P Y. Safety evaluation of allelochemical derivative pyrone on different millet varieties. Chinese Journal of Applied Ecology, 2020, 1(7): 2236-2242. (in Chinese)

[38] GUO M J, BAI Y Q, GAO P, SHEN J, DONG S Q, YUAN X Y, GUO P Y. Effect of MCPA on leaf senescence and endogenous hormones content in leaves of foxtail millet seedlings. Scientia Agricultura Sinica, 2020, 53(3): 513-526. (in Chinese)

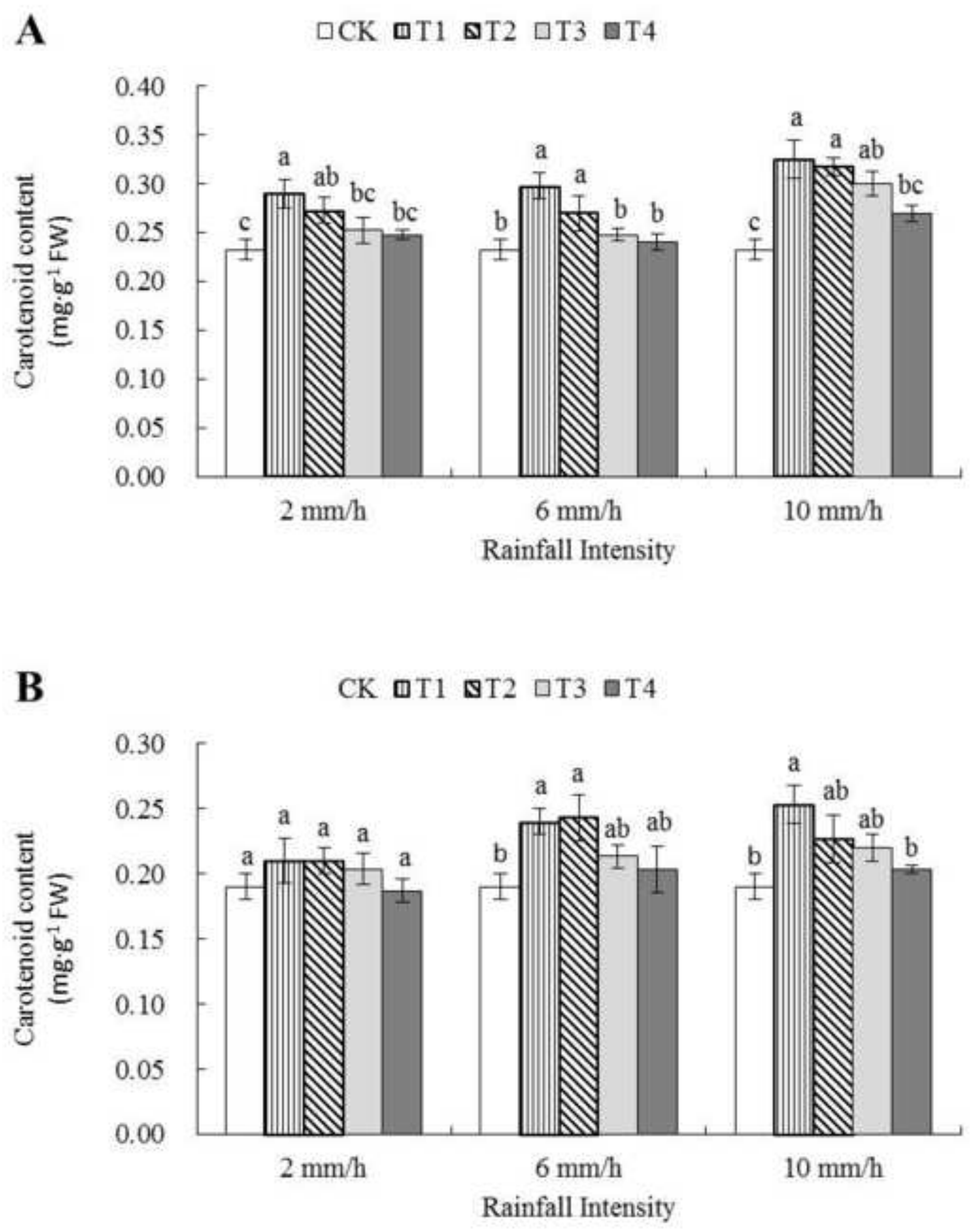
A
□CK ▥T1 ▧T2 □T3 ■T4
Carotenoid content (mg·g-1 FW)
0.40
0.35
0.30
0.25
0.20
0.15
0.10
0.05
0.00
c a ab bc bc
b a a b b
c a a ab bc
2 mm/h
6 mm/h
10 mm/h
Rainfall Intensity
B
CK ▥T1 ▧T2 □T3 ■T4
Carotenoid content (mg·g-1 FW)
0.30
0.25
0.20
0.15
0.10
0.05
0.00
a a a a a
b a a ab ab
b a ab ab b
2 mm/h
6 mm/h
10 mm/h
Rainfall Intensity

Figure 2

Click here to access/download;Figure;Figure 2.jpg

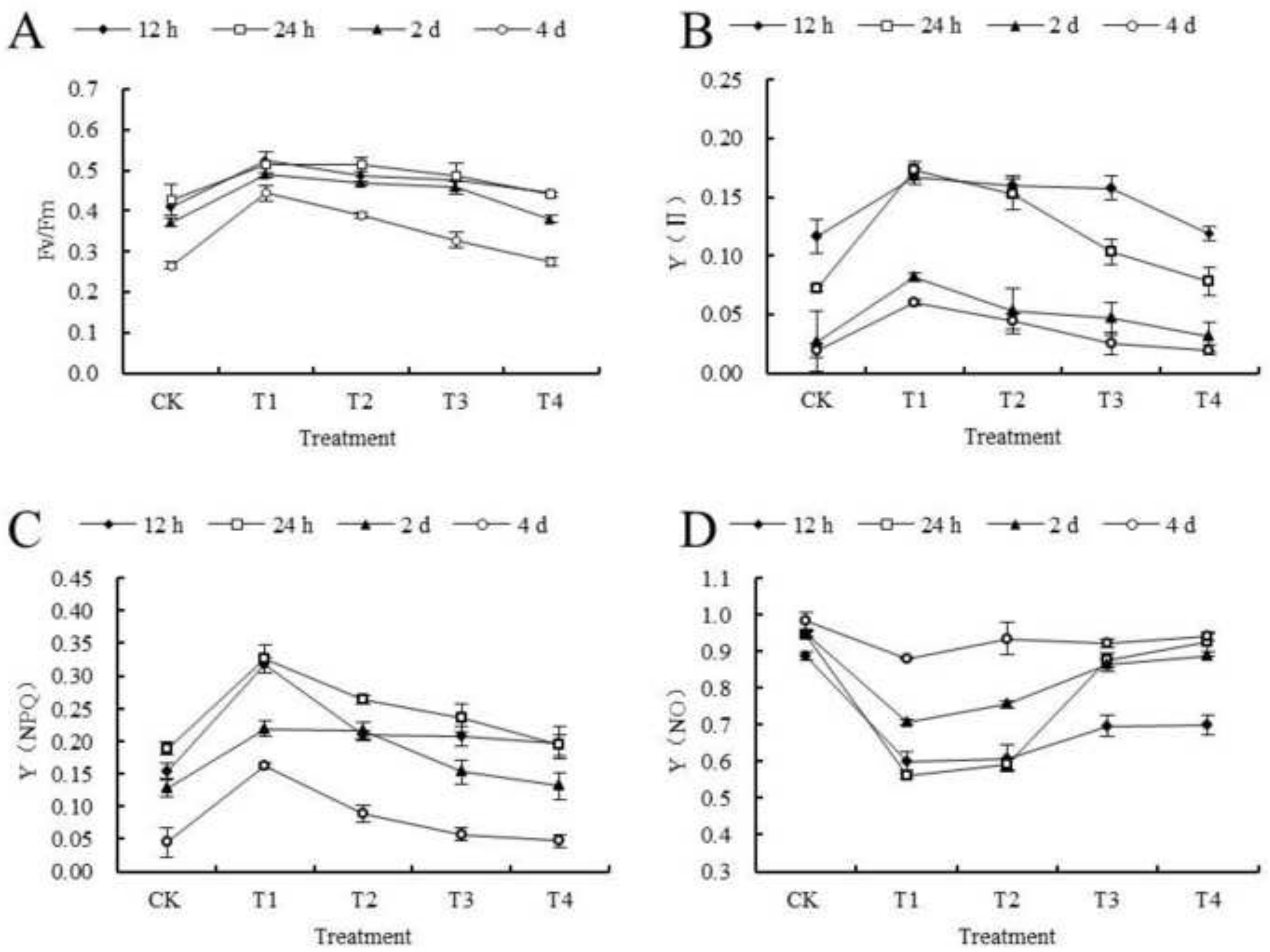

Figure 3

Click here to access/download;Figure;Figure 3.jpg

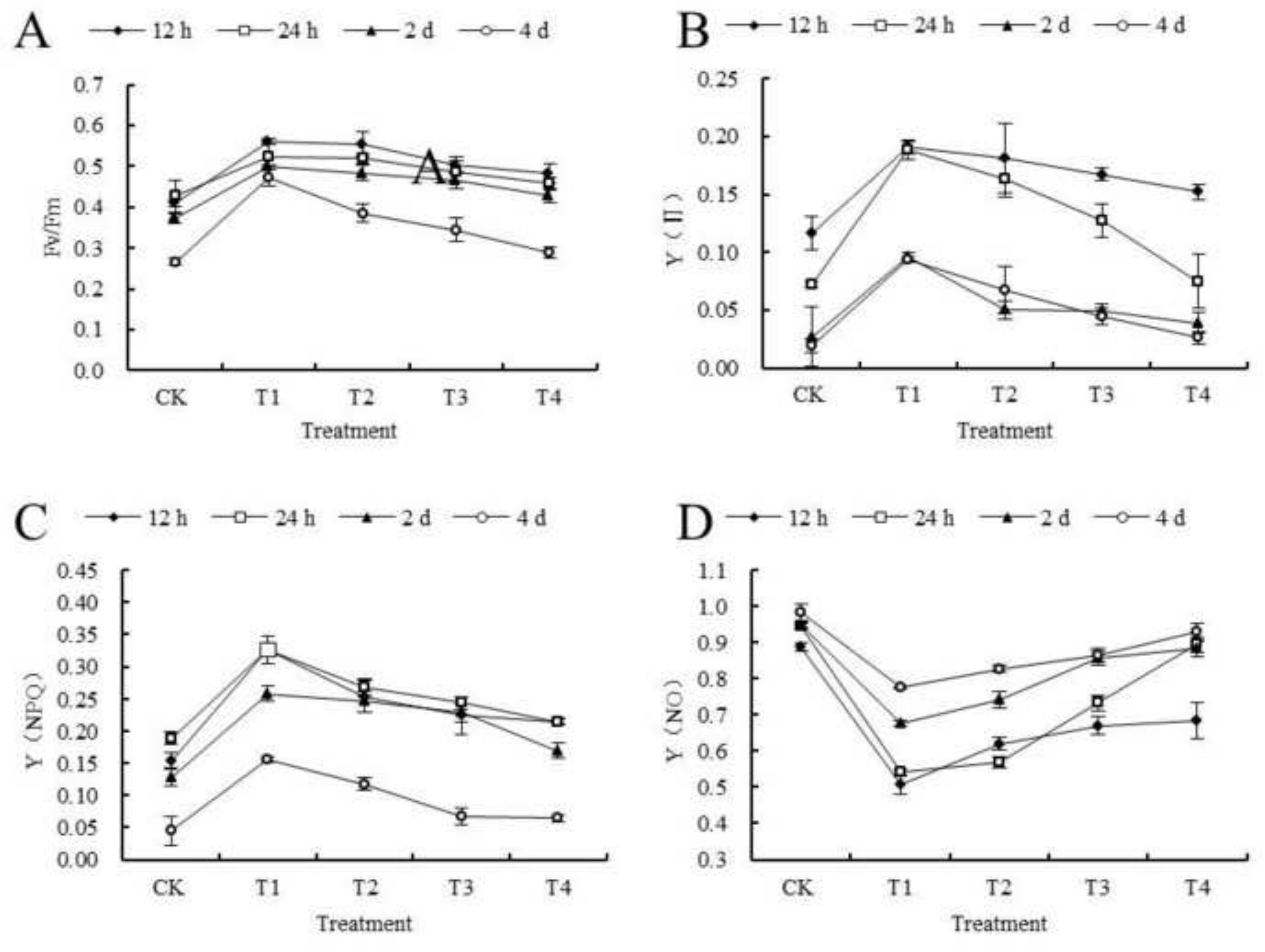

Figure 4

Click here to access/download;Figure;Figure 4.jpg

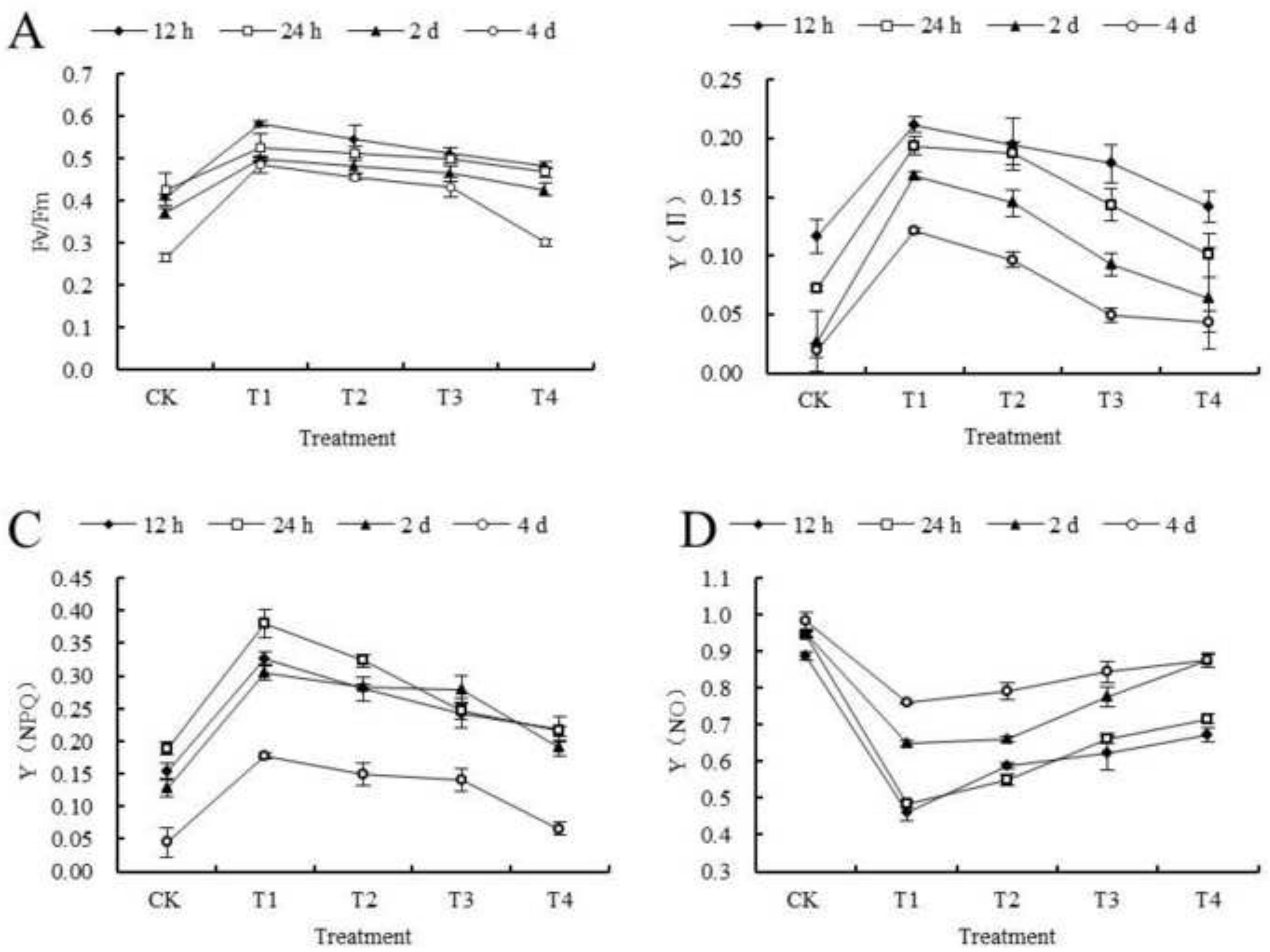

Table

Click here to access/download
Supporting Information
table.docx
