# Supplementary material for: Effects of mesotrione on the control efficiency and chlorophyll fluorescence parameters of Chenopodium album under simulated rainfall conditions
Source: PLoS One. 2022 Jun 3;17(6):e0267649. doi: 10.1371/journal.pone.0267649 (PMC9165882; doi:10.1371/journal.pone.0267649)
Supplement: Attachment — Submitted filename: PONE-D-21-13877_R1.pdf [file pone.0267649.s012.pdf]

PLOS ONE

Effects of mesotrione on the control efficiency and chlorophyll fluorescence parameters of Chenopodium album under simulated rainfall conditions

--Manuscript Draft--

**Manuscript Number:**	PONE-D-21-13877R1
**Article Type:**	Research Article
**Full Title:**	Effects of mesotrione on the control efficiency and chlorophyll fluorescence parameters of Chenopodium album under simulated rainfall conditions
**Short Title:**	Effects of mesotrione on Chenopodium under simulated rainfall condations
**Corresponding Author:**	Meijun Guo<br>Jinzhong University<br>Yuci, CHINA
**Keywords:**	Mesotrione; Chenopodium album; Rainfall; Weed Control effect; Chlorophyll fluorescence
**Abstract:**	This experiment was conducted to study the effects of mesotrione on the control efficiency and chlorophyll fluorescence parameters of Chenopodium album. Simulating three rainfall intensities of 2 mm/h (light rain), 6 mm/h (moderate rain) and 10 mm/h (heavy rain) at different interval times (0.5 h, 1 h, 2 h, 4 h[illegible]nalyze variable regulation of the control effect, the photosynthetic pigment content a[illegible]chlorophyll fluorescence parameters of C. album after spraying mesotrione. With the extension of rainfall time interval, the inhibition rate of plant height, plant control effect and fresh weight control effect of C. album were gradually increased, the inhibition effect of rainfall on the efficacy was gradually decreased, at the same time, the contents of chlorophyll a, chlorophyll b, carotenoids, the maximum photochemical quantum efficiency (Fv/Fm), the actual photochemical quantum yield (Y(II)) and quantum yield(Y(NO)) production of regular energy consumption of C. album were also increased, while the nonregulatory energy decreased gradually. The results showed that the contents of chlorophyll a and chlorophyll b in leaves of C. album increased significantly by 35.63% and 35.38% compared with the control under the condition of simulating 6 mm/h in interval 1 hour. The study suggested that simulating 10 mm/h rainfall intensity had the greatest effect on C. album, the photosynthetic pigment content, Fv/Fm and Y(II) of leaves were significantly higher than those in the control groups under 0.5 h, 1 h and 2 h interval treatments. The carotenoid content was the lowest and Y(NO) was the largest under the 4 h interval treatment. As is displayed that rainfall reduced the weed control effect in the aspect of controlling C. album on mesotrione, which is partly contributed to increase photosynthetic pigment content and enhance the PS II photochemical activity. In conclusion, the rain intensity of ≤2 mm/h did not affect the control effect of mesotrione on C. album. At 6 mm/h within 1 h after treatment, the control effect of fresh weight was significantly reduced by more than 7.14%, and at 10 mm/h within 2 h, the control effect was significantly reduced by more than 14.78%.
**Order of Authors:**	Meijun Guo
	Mengmeng Sun
	Shuai Guo
	Yanfen Li
	Shuqi Dong
	Xie Song
	Xiangyang Yuan
	Xiaoxin Shi
**Response to Reviewers:**	Thank you for your letter and for the reviewers' comments concerning our manuscript . Those precious comments are all valuable and very helpful for revising and improving our paper, as well as the important guiding significance to our researches. We have studied comments carefully and have made correction which we hope meet with

Powered by Editorial Manager® and ProduXion Manager® from Aries Systems Corporation

	approval.
**Additional Information:**	
**Question**	**Response**
**Financial Disclosure**<br><br>Enter a financial disclosure statement that describes the sources of funding for the work included in this submission. Review the submission guidelines for detailed requirements. View published research articles from *PLOS ONE* for specific examples.<br><br>This statement is required for submission and **will appear in the published article** if the submission is accepted. Please make sure it is accurate.<br><br>**Unfunded studies**<br>Enter: *The author(s) received no specific funding for this work.*<br><br>**Funded studies**<br>Enter a statement with the following details:<br>• Initials of the authors who received each award<br>• Grant numbers awarded to each author<br>• The full name of each funder<br>• URL of each funder website<br>• Did the sponsors or funders play any role in the study design, data collection and analysis, decision to publish, or preparation of the manuscript?<br>• **NO** - Include this sentence at the end of your statement: *The funders had no role in study design, data collection and analysis, decision to publish, or preparation of the manuscript.*<br>• **YES** - Specify the role(s) played.<br><br>* typeset	This paper was supported by the system of National Modern Agriculture Technology (CARS-06-13.5-A28), the program for the Top Young Innovative Talents of Shanxi Agricultural University (TYIT201406) and the Key research and development plan of Shanxi Province (201903D22103).
**Competing Interests**<br><br>Use the instructions below to enter a competing interest statement for this submission. On behalf of all authors, disclose any competing interests that could be perceived to bias this work—acknowledging all financial support and any other relevant financial or non-financial competing interests.	The authors have declared that no competing interests exist.

Powered by Editorial Manager® and ProduXion Manager® from Aries Systems Corporation

This statement is required for submission and **will appear in the published article** if the submission is accepted. Please make sure it is accurate and that any funding sources listed in your Funding Information later in the submission form are also declared in your Financial Disclosure statement.<br><br>View published research articles from *PLOS ONE* for specific examples.<br><br>**NO authors have competing interests**<br><br>Enter: *The authors have declared that no competing interests exist.*<br><br>**Authors with competing interests**<br><br>Enter competing interest details beginning with this statement:<br><br>*I have read the journal's policy and the authors of this manuscript have the following competing interests: [insert competing interests here]*<br><br>* typeset	
**Ethics Statement**<br><br>Enter an ethics statement for this submission. This statement is required if the study involved:<br><br>• Human participants<br>• Human specimens or tissue<br>• Vertebrate animals or cephalopods<br>• Vertebrate embryos or tissues<br>• Field research<br><br>Write "N/A" if the submission does not require an ethics statement.<br><br>General guidance is provided below. Consult the submission guidelines for detailed instructions. **Make sure that all**	No

**information entered here is included in the Methods section of the manuscript.**<br><br>**Format for specific study types**<br><br>**Human Subject Research (involving human participants and/or tissue)**<br>• Give the name of the institutional review board or ethics committee that approved the study<br>• Include the approval number and/or a statement indicating approval of this research<br>• Indicate the form of consent obtained (written/oral) or the reason that consent was not obtained (e.g. the data were analyzed anonymously)<br><br>**Animal Research (involving vertebrate animals, embryos or tissues)**<br>• Provide the name of the Institutional Animal Care and Use Committee (IACUC) or other relevant ethics board that reviewed the study protocol, and indicate whether they approved this research or granted a formal waiver of ethical approval<br>• Include an approval number if one was obtained<br>• If the study involved *non-human primates*, add *additional details* about animal welfare and steps taken to ameliorate suffering<br>• If anesthesia, euthanasia, or any kind of animal sacrifice is part of the study, include briefly which substances and/or methods were applied<br><br>**Field Research**<br><br>Include the following details if this study involves the collection of plant, animal, or other materials from a natural setting:<br>• Field permit number<br>• Name of the institution or relevant body that granted permission	
**Data Availability**<br><br>Authors are required to make all data underlying the findings described fully available, without restriction, and from the time of publication. PLOS allows rare exceptions to address legal and ethical	Yes - all data are fully available without restriction

concerns. See the PLOS Data Policy and FAQ for detailed information.<br><br>A Data Availability Statement describing where the data can be found is required at submission. Your answers to this question constitute the Data Availability Statement and **will be published in the article**, if accepted.<br><br>**Important:** Stating 'data available on request from the author' is not sufficient. If your data are only available upon request, select 'No' for the first question and explain your exceptional situation in the text box.<br><br>Do the authors confirm that all data underlying the findings described in their manuscript are fully available without restriction?	
**Describe where the data may be found in full sentences. If you are copying our sample text, replace any instances of XXX with the appropriate details.**<br><br>• If the data are **held or will be held in a public repository**, include URLs, accession numbers or DOIs. If this information will only be available after acceptance, indicate this by ticking the box below. For example: *All XXX files are available from the XXX database (accession number(s) XXX, XXX.*).<br>• If the data are all contained **within the manuscript and/or Supporting Information files**, enter the following: *All relevant data are within the manuscript and its Supporting Information files.*<br>• If neither of these applies but you are able to provide **details of access elsewhere**, with or without limitations, please do so. For example:<br><br>*Data cannot be shared publicly because of [XXX]. Data are available from the XXX Institutional Data Access / Ethics Committee (contact via XXX) for researchers who meet the criteria for access to confidential data.*	All relevant data are within the manuscript and its Supporting Information files.

The data underlying the results presented in the study are available from (include the name of the third party and contact information or URL).<br>• This text is appropriate if the data are owned by a third party and authors do not have permission to share the data.<br><br>* typeset	
Additional data availability information:	

Powered by Editorial Manager® and ProduXion Manager® from Aries Systems Corporation

Effects of mesotrione on the control efficiency and chlorophyll fluorescence parameters of *Chenopodium album* under simulated rainfall conditions

Mengmeng Sun[1¶], Meijun Guo[2¶], Shuai Guo[1], Yanfen Li[1], Shuqi Dong[1], Xie Song[1], Xiaoxin Shi[3], Xiangyang Yuan[1*]

[1]Laboratory of Crop Chemical Regulation and Chemical Weed Control, College of Agronomy, Shanxi Agricultural University, Taigu, Shanxi, China

[2]Department of biological science and technology, Jinzhong University, Jinzhong, Shanxi, China

[3] Taigu County Meteorological Bureau, Shanxi, China

[*]Corresponding author's E-mail: yuanxiangyang200@163.com(YXY)

[¶]These authors contributed equally to this work. SM and GM are co-first authors on this work.

Abstract:

This experiment was conducted to study the effects of mesotrione on the control efficiency and chlorophyll fluorescence parameters of *Chenopodium album*. Simulating three rainfall intensities of 2 mm/h (light rain), 6 mm/h (moderate rain) and 10 mm/h (heavy rain) at different interval times (0.5 h, 1 h, 2 h, 4 h)to analyze variable regulation of the control effect, the photosynthetic pigment content and chlorophyll fluorescence parameters of *C. album* after spraying mesotrione. With the extension of rainfall time interval, the inhibition rate of plant height, plant control effect and fresh weight control effect of *C. album* were gradually increased, the inhibition effect of rainfall on the efficacy was gradually decreased, at the same time, the contents of chlorophyll a, chlorophyll b, carotenoids, the maximum photochemical quantum efficiency (Fv/Fm), the actual photochemical quantum yield (Y(II)) and quantum yield(Y(NO)) production of regular energy consumption of *C. album* were also increased, while the nonregulatory energy decreased gradually. The results showed that the contents of chlorophyll a and chlorophyll b in leaves of *C. album* increased significantly by 35.63% and 35.38% compared with the control under the condition of simulating 6 mm/h in interval 1 hour. The study suggested that simulating 10 mm/h rainfall intensity had the greatest effect on *C. album*, the photosynthetic pigment content, Fv/Fm and Y(II) of leaves were significantly higher than those in the control groups under 0.5 h, 1 h and 2 h interval treatments. The carotenoid content was the lowest and Y(NO) was the largest under the 4 h interval treatment. As is displayed that rainfall reduced the weed control effect in the aspect of controlling *C. album* on mesotrione, which is partly contributed to increase photosynthetic pigment content and enhance the PS II photochemical activity. In conclusion, the rain intensity of ≤2 mm/h did not affect the control effect of mesotrione on *C. album*. At 6 mm/h within 1 h after treatment, the control effect of fresh weight was significantly reduced by more than 7.14%, and at 10 mm/h within 2 h, the control effect was significantly reduced by more than 14.78%.

Keywords: Mesotrione; *Chenopodium album*; Rainfall; Weed Control effect; Chlorophyll fluorescence

1 Introduction

Weed would reduce the yield and quality of the crops, hinder mechanized sowing and harvest and then slow down the process of agricultural modernization [1-2]. *Chenopodium album*. L is an erect annual herbaceous plant, that grows between 0.2 and 2 meters in height, striped green, red or purple stems. Leaves are rhomboid, deltoid to lanceolate, upper entire, lower toothed or irregularly lobed. The leaves are wax-coated, sometimes has mealy and unregular sawtooth on the edge of them, with a whitish coat on the underside. The length of its petiole is close to blade or half of the blade [3]. *C. album* is one of the common farmland malignant weed in northern China. It has strong environmental adaptability and a large root system, even grow on the Tibetan Plateau and in the lowlands of southwest China, so the *C. album* is recorded as a potherb in both plateaus and low lands [4]. It has obvious advantages when competing with crops for water and fertilizer, every single plant could yield 3000-20000 seeds (Data from CABI), which led to a sharp decline in crop yield [5-6]. Sugarbeet, barley, mustard, gram, maize and so many crops were defeated by C. album, it has been reported to reduce the soybean (Glycine max) yield by 61%, in the wheat (T[illegible]icum aestivum) field, the yield loss approximately 50-60%, which also was associated with interference of this weed [7-9]. When the density increases to 20 plants per m^2, the corn would not ear because of the shade effect of *C. album* [10].

Mesotrione is a herbicide of broad-spectrum, selective stem and leaf before bud and after seedling mainly used to effectively control broad-leaved weeds and some gramineous weeds by inhibiting the catalytic factors of plant photosynthetic process. Mesotrione is widely used in weed control of maize and winter wheat due to its high activity, low residue, strong compatibility and safety to the environment and subsequent crops [11-12]. Summer is the key period for crop growth

and weeding. However, local microclimate and severe convective weather occur frequently, and rainfall interfere the herbicide efficacy due to the scouring every year [13]. Therefore, it is attached importance to study the influence of rainfall on herbicides. There are great differences in the absorption rate and rain resistance among different stem and leaf treatment agents, so the interval time of affecting the efficacy is different, but most herbicides will not affect the weed when rainfall after 4-6 h [14]. Wu *et al*. [15] found that rainfall after the application had a significant inhibitory effect on the efficacy of nicosulfuron methyl; paraquat could effectively control *Alopecurus aequalis* after 0.5 h and 1 h rainfall [16]; there was no significant difference in bensulfuron efficacy between without rainfall and after 2 h rainfall [17]; Wang *et al*. [18] showed that the effect of imazethapyr was no longer affected under the 9 mm/h rainfall intensity. When the interval time of rainfall is 0.5 h, the effect of rainfall on barnyardgrass was significantly reduced, it's down to 19.42% [19]. At present, it is known that mesotrione has effects on the growth of weeds and crops, such as reducing the plant height of maize, making *Amaranthus retroflexus* heart leaves be yellow, reducing the photosynthetic pigment content, reducing the maximum photochemical quantum efficiency (Fv/Fm) and the actual photochemical quantum yield Y(II) [20-22]. The inhibition rate of plant height, plant control effect and fresh weight control effect can well reflect the control effect of herbicides on weeds; photosynthetic pigment content reflects the green extent of plants; chlorophyll fluorescence parameters as a probe of photosynthetic capacity of leaves can reflect the status of weeds under herbicide stress [23]. Most of the studies on mesotrione focused on its control effect and crop safety. There were no reports on the control effect and chlorophyll fluorescence parameters of mesotrione on broad-leaved weed *C. album* under rainfall conditions. In this experiment, artificial rainfall was used to simulate rainfall, and *C. album* is a common malignant weed in the field, was used to simulate different rain intensities at different intervals after spraying mesotrione.

In order to assess the possibility of mesotrione application and understand the related mechanism, we investigated the weed control, agronomic traits, photosynthetic pigment content and

the chlorophyll fluorescence parameters of *C. album* to mesotrione in this current study. The influence of rain intensity and interval time on the efficacy was determined, which provide a theoretical basis for the use of mesotrione.

2 Materials and methods

2.1 Experimental site

Field experiments were conducted at farming station of Shanxi Agricultural University during 2019, Jinzhong, China (37.43°N, 112.61°E). The area is located in the northeast of Jinzhong region and belongs to a warm temperate continental monsoon climatic with an altitude of approximately 800 meters, the annual average temperature was 9.9 ℃, and the frost-free period was about 159 days. Annual precipitation of the province was 400-650 mm, but the seasonal distribution was uneven, with more than 60% of precipitation concentrated in June to August [24]. Tables 1 and 2 present the meteorological conditions and soil characteristics of experimental site.

Table 1. Meteorological data of the experimental sites during May-September in 2019

Month	Precipitation(mm)	The max rainfall intensity(mm/h)	Temperature(℃)			≥20℃accumulated temperatur[illegible])	Sunshine hours(h)
			Average	Min	Max		
5	1	0.8	19.9	35.3	2.6	235.1	258.9
6	55.7	9.3	24.5	35.6	12.4	715	213.4
7	65.1	13.4	25.4	37.1	10.8	788.4	243.1
8	52	5.2	22.9	34.5	9.7	614.2	219.6
9	78.8	6	18.4	34.1	6.6	195.5	223.9

Table 2. The basic physical and chemical properties of soil

Depth of soil layer	pH	Available potassium (mg/kg)	Available phosphorus (mg/kg)	Available nitrogen (mg/kg)	organic matter (g/kg)
0-5 cm	8.21	484.21	43.70	64.20	8.52
5-10 cm	8.14	471.93	26.79	42.80	8.52
10-15 cm	8.17	319.60	21.28	49.93	7.99

2.2 Experimental design

Four rainfall intervals were set: 0.5 h (T1), 1 h (T2), 2 h (T3), 4 h (T4), and no rainfall after mesotrione application was taken as control (CK). The completely randomized design was conducted by the field experiment with three replicates in 4 m^2 (2 m × 2 m). The recommended dose (3 L/h[illegible]m^2) of 10% mesotrione emulsifiable concentrate (EC) (Shandong Shengbanglvye Chemical Limited Company) was sprayed by the watering can.

2.3 Rainfall designs

According to the standard of China Meteorological Administration(CMA): the hourly rainfall is 0.1～2.5 mm/h for light rain, 2.6～8 mm for moderate rain, and 8.1～15.9 mm for heavy rain. Simulating light rain 2 mm/h, moderate rain 6 mm/h and heavy rain 10 mm/h by power operated sprayers (Lufeng, 3 WBD-20 C, 20 L). According to the calculation method that 1 L of rainfall per square meter is 1 mm [25], artificial spraying was carried out by simulating natural rainfall, and the height of the spray is 0.5 meters. By adjusting the knob on the edge of the sprayer, 4 L, 12 L, and 20 L of water are sprayed out within half an hour to obtain three rain intensities of 2 mm/h, 6 mm/h and 10 mm/h. Before the experiment, several tests were carried out with the meteorological rain gauge. Firstly, we screened out one type of shower that sprayed the smallest water and most homogeneous when spraying, then the positions of different rain intensities on the sprayer knob

were accurately marked. The process was repeated until the desired rainfall amounts and intensities were achieved.

To prevent drift effects in field conditions, we put many baffles around the rainfall simulation areas of the neighborhood. The simulated rainfall is carried out under calm or breezy weather to reduce the error, and the test rain intensity is determined again every time the simulated rainfall is performed, the error of these results is within 0.2 mm/h.

2.4 Data collections

2.4.1 Determination of weed control effect

To calculate the reductions in weed number and biomass, the shoots of all *C. album* plants were cut from three 0.25 m^2 (0.5 m × 0.5 m) quadrats in each plot (2 m × 2 m) at 15 and 30 days after treatment (DAT) [26]. When mesotrione in soil was analyzed, *C. album* were collected carefully by taking out from the soil in the open field. The plant was weighed after washing, the biomass of each weed sample were measured. The efficacy and reduction was calculated by this equation:

$$Efficacy\ (\%) = [(C - B)/C] \times 100 \quad (1)$$

where C is the aboveground height of the nontreated control plot and B is the height of an treated plot.

$$Reduction\ (\%) = [(SCP - TP)/SCP] \times 100 \quad (2)$$

Where TP is the weed plant number or biomass in the treated plots and SCP is the sum of the plant number or biomass in the weedy controls.

2.4.2 Determination of photosynthetic pigment content

Photosynthetic data was collected after 3, 6 and 9 d, weight 0.05 g of mature and intact leaves with the same growth in the middle and upper part of the plant, cut them into pieces, and put them

into a stoppered test tube containing 5 ml of 96% ethanol, and soak in a dark place at room temperature for 48 hours until the leaves are milky white and oscillate for several hours during the extraction process. The absorbance of the extract is measured at wavelengths of 470 nm, 649 nm and 665 nm, and the content of chlorophyll a, chlorophyll b, and carotenoid are calculated [27]. The calculation formulas are in the following:

$$\mathrm{Ca} = 13.95A_{665} - 6.88\,A_{649} \quad (3)$$

$$\mathrm{Cb} = 24.96\,A_{649} - 7.32A_{665} \quad (4)$$

$$\mathrm{Cx.c} = (1000\,A_{470} - 2.05\,\mathrm{Ca} - 114.8\,\mathrm{Cb})/245 \quad (5)$$

2.4.3 Determination of chlorophyll fluorescence parameters

The chlorophyll fluorescence parameters were tested at 12 h, 24 h, 2 d and 4 d after application respectively. The middle and upper parts of the plant were selected to be fully stretched and mature leaves. The MINI-PAM-Ⅱ portable pulse modulated chlorophyll fluorometer (Walz, Germany), first use the leaf clip of the fluorometer to clamp the *C. album* for 30 min. Actual photochemical efficiency Y(II), maximum photochemical quantum yield Fv/Fm of leaves, regulated energy dissipation quantum yield Y(NPQ) and non-regulated energy dissipation quantum yield Y(NO) were examined [28].

2.5 Statistical analyses

DPS 6.5 Analysis System was used to conduct all statistical analyses. Data were presented as mean ± standard error of the mean. Duncan’s test was used to analyze significant differences among treatments in growth parameters, chlorophyll content and fluorescence parameters.

3 Results

3.1 The visual control effect

The reaction symptoms of *C. album* to the mesotrione become more serious with the time going of application. With the extension of rainfall interval, the growth of *C. album* was severely inhibited, the tip of leaves was more serious, and the heart leave was even completely withered and shed. The survey of weed injury level (Table 3) showed that rainfall reduced the herbicide injury of weed by 1~2 levels at 15 DAT. The weed damage decreased to grade 5 when the rain intensity was 10 mm/h and the interval was 0.5 h at 30 DAT, but other intervals is grade 6.

Table 3. Weed injury level at 15, 30 d after application of mesotrione and simulated rainfall

Rainfall intensity	Interval time	Level of weed injury	
		15 d	30 d
2 $mm \cdot h^{-1}$	CK	6	6
	T1	5	6
	T2	5	6
	T3	6	6
	T4	6	6
6 $mm \cdot h^{-1}$	CK	6	6
	T1	4	6
	T2	5	6
	T3	5	6
	T4	5	6
10 $mm \cdot h^{-1}$	CK	6	6
	T1	4	5
	T2	4	6
	T3	5	6
	T4	6	6

3.2 The effect of simulated rainfall on mesotrione

After spraying mesotrione, *C. album* exhibits disease like chlorosis and albino of stems and leaves. With applicating the herbicide, the rainfall causes the plant height inhibition rate to decrease, and enhanced gradually increases with the extension of the interval. It is represented in (Table 4) that when the rain intensity is 2 mm/h, at 15 DAT, compared with CK, T1,T2 and T3 cause a significant reductions in the inhibition rate of plant heigh[illegible](1[illegible].80%、10.87%、9.70%, respectively). At 30 DAT, there were no significant difference between treatments and CK. When the rain intensity was 6 mm/h, after 30 days, the inhibition rate of the treatment with only T1 interval significantly decreased by 5.57% compared with CK, while the other treatments had no significant difference compared with CK. In addition to, with the increase of rainfall intensity, there was significantly reduced by 7.53% and 4.91% at T1 and T2 in the inhibition rate of plant height in 10 mm/h, the reduced efficiency were 8.93% and 5.83%, respectively.

The control effect and fresh weight control effect of the plants are belong to the weed control effects, which is increased significantly at 30 DAT compared with 15 DAT. At 15 DAT, the weed control effects of only T1 and T2 were significantly lower than that of CK by 15.06%, 11.83%, 13.31%, and 11.38% in 2 mm/h, the reduced efficiency was more than 13 percent; the rain intensity was at 6 mm/h and 10 mm/h, the weed control effects were significantly lower than CK. At 30 DAT, when the rain intensity was 2 mm/h, the weed control effects were still more than 90% at all treatments. However, the fresh weight control effect of T1 and T2 treatments is significantly lower than CK by 16.34% and 6.90% in 6 mm/h, the reduced efficiency were 16.90% and 7.14%, respectively; compared with CK, T1, T2 and T3 were reduced in the fresh weight control (19.30%, 8.43%, 6.80%, respectively) when the rain intensity is 10 mm/h, and the reduced efficiency was above 7%.

Table 4. Effect of simulated rainfall on the weed control of mesotrione

Rainfall Intensity	Interval time	Plant height inhibition rate/%				Plant control efficacy/%				Fresh weight control efficacy/%			
		15 d	Reduced efficiency	30 d	Reduced efficiency	15 d	Reduced efficiency	30 d	Reduced efficiency	15 d	Reduced efficiency	30 d	Reduced efficiency
2 mm·h^{-1}	CK	65.29±1.81a	——	84.29±1.14a	——	83.09±1.78a	——	96.42±3.39a	——	81.91±2.71a	——	96.67±3.01a	——
	0.5 h	53.49±2.10b	18.07	80.79±2.11a	4.15	68.03±4.22b	18.12	91.20±5.12a	5.41	68.60±2.74b	16.25	93.89±2.83a	2.88
	1 h	54.42±1.00b	16.65	81.85±3.06a	2.89	71.26±3.73b	14.24	93.68±2.80a	2.54	70.53±5.82b	13.89	94.39±2.85a	2.36
	2 h	55.59±1.95b	14.86	82.37±2.24a	2.28	77.92±5.58ab	6.22	95.32±1.37a	1.14	76.45±3.67ab	6.67	95.28±3.54a	1.44
	4 h	62.12±2.68a	4.86	83.94±1.89a	0.42	81.84±3.38ab	1.50	96.08±7.82a	0.35	80.26±4.78a	2.01	95.74±2.53a	0.96
6 mm·h^{-1}	CK	65.29±1.81a	——	84.29±1.14a	——	83.09±1.78a	——	96.42±3.39a	——	81.91±2.71a	——	96.67±3.01a	——
	0.5 h	41.19±1.42c	36.91	78.72±2.67b	6.61	43.00±4.39c	48.25	76.98±4.01c	20.16	51.16±4.82c	37.54	80.33±3.20c	16.90
	1 h	43.27±2.06c	33.73	81.02±1.84ab	3.88	56.69±2.46b	31.77	85.21±7.30b	11.60	54.92±4.39c	32.95	89.77±6.00b	7.14
	2 h	50.71±1.72b	22.33	82.14±2.46a	2.55	63.30±3.94b	23.82	89.50±5.51b	7.18	64.42±3.43b	21.35	94.28±3.68a	2.47
	4 h	54.83±1.16b	16.02	83.44±1.81a	1.01	65.73±2.37b	20.89	90.41±4.72b	6.23	68.74±4.01b	16.07	96.28±2.15a	0.40
10 mm·h^{-1}	CK	65.29±1.81a	——	84.29±1.14a	——	83.09±1.78a	——	96.42±3.39a	——	81.91±2.71a	——	96.67±3.01a	——
	0.5 h	40.32±2.38c	38.24	76.76±1.98c	8.93	39.20±3.63c	52.82	68.96±3.63d	28.48	43.09±2.45c	47.39	77.37±2.12c	19.96
	1 h	42.86±1.61bc	34.35	79.38±2.93bc	5.83	42.96±1.92c	48.30	78.66±2.51c	18.42	55.12±5.23b	32.70	88.20±4.31b	8.76
	2 h	47.16±1.05bc	27.77	82.12±1.30ab	2.57	55.90±2.61bc	32.72	82.17±1.53c	14.78	57.16±4.23b	30.22	89.87±3.39b	7.03
	4 h	53.94±0.99b	17.38	82.36±1.28ab	2.29	65.46±1.17b	21.22	87.68±3.43b	9.06	62.08±3.62b	24.21	94.09±4.09a	2.67

Data are shown as mean ±SE with the different lowercase letters in the same column represent significant differences ($P<0.05$).

3.3 Effects of simulated rainfall on photosynthetic pigment content of *C. album* leaves after spraying mesotrione

3.3.1 The effect of simulated rainfall on the carotenoid content of *C. album* after spraying mesotrione

The data in Figure 1 showed that the corresponding rainfall intensity was 2 mm/h, compared with CK at 3 DAT, T1 and T2 treatments show a significant increase, 24.73% and 17.2%. Compared with all interval treatments, CK at 6 DAT did not see a big difference, which could indicate that the rainfall in this period is not capable of preventing the albino process of *C. album*.

When the rainfall intensity reached 6 mm/h, it could find that the corresponding carotenoid content of T1 and T2 treatments increased at 3 and 6 DAT, and the ranges were 21.85%, 13.89%, 20.00% and 21.91%, respectively. Except that the T4 interval treatment was a bit different, the carotenoid content involved in other treatments looked higher than the content contained in CK. And the growth rates are 39.78%, 36.56%, 29.03% and 33.32%, 19.32%, 15.79%.

Figure1: Effect of simulated rainfall on carotenoid content in leaves of *C. album* treated by mesotrione after 3(A) and 6(B) DAT. X-axis represents different rainfall intensity; y-axis represents the carotenoid content of leaves. CK means that there was no rain after the application of mesotrione, T1, T2, T3, and T4 represent different rainfall interval times (0.5 h, 1 h, 2 h, and 4 h). Effect of simulated rainfall on carotenoid content in leaves of *C. album* treated by mesotrione after 3 DAT (A) and 6 DAT (B). Lowercase letters in the same column indicate significant differences at the 0.05 level.

3.3.2 The effect of simulated rainfall on the chlorophyll content of *C. album* after spraying mesotrione

Like the carotenoid content, the content of chlorophyll a and chlorophyll b decreased gradually

and showed a decreasing trend with the extension of rainfall interval. As shown in Table 5, when the rain intensity was 2 mm/h, the chlorophyll a and chlorophyll b contents of all interval treatments were not significantly different than CK at 6 DAT. At the same time, chlorophyll a+b content under T1 treatment has an increase of 27.86% compared with CK, the difference was not very significant between the other treatments and CK.

Under the situation of 3 DAT, the rainfall intensity reached 6 mm/h. In this case, in addition to the different results produced by T4 processing, the chlorophyll a content corresponding to other treatments has a more significant increment compared to CK. In addition, when the T1 and T2 intervals were used for treatment, the chlorophyll b content would increase significantly, with an increase of 46.41% and 35.38%. On the other hand, all treatments at certain intervals show that the contents of chlorophyll a and chlorophyll b were much higher than that situation of CK at 6 DAT. It could be claimed that compared with carotenoids, 6 mm/h rainfall had a greater impact on chlorophyll a and b. Although the interval is only 4 hours, it has a higher content than CK. When the rainfall intensity under 3 DAT reached 10 mm/h, the chlorophyll a content corresponding to all treatment intervals was at a high level, exceeding the chlorophyll content of CK. In the meantime, there was no significant difference between T1, T2 and T3 treatment.

Table 5. Effect of simulated rainfall on photosynthetic pigment content in leaves of *C. album* treated by mesotrione

Rainfall Intensity	Interval time	Chlorophyll a conten[illegible]g·g^{-1} FW)		Chlorophyll b content (mg·g^{-1} FW)		Chlorophyll a+b conten[illegible]•g^{-1} FW)	
		3 d	6 d	3 d	6 d	3 d	6 d
2 mm·h^{-1}	CK	0.92±0.058b	0.79±0.101a	0.27±0.020c	0.26±0.027a	1.19±0.040c	1.05±0.075b
	T1	1.39±0.021a	1.00±0.078a	0.46±0.012a	0.35±0.062a	1.85±0.012a	1.35±0.019a
	T2	1.06±0.098b	0.90±0.049a	0.38±0.012b	0.31±0.030a	1.44±0.087b	1.21±0.020ab
	T3	1.09±0.074b	0.82±0.074a	0.34±0.023b	0.32±0.027a	1.43±0.054b	1.14±0.048b
	T4	0.95±0.040b	0.79±0.045a	0.35±0.0208b	0.28±0.025a	1.30±0.030bc	1.07±0.069b
6 mm·h^{-1}	CK	0.92±0.058b	0.79±0.101b	0.27±0.020c	0.26±0.027b	1.19±0.04c	1.05±0.075b
	T1	1.29±0.075a	1.18±0.078a	0.51±0.021a	0.47±0.009a	1.80±0.01a	1.65±0.086a
	T2	1.24±0.031a	1.14±0.067a	0.37±0.035b	0.46±0.012a	1.57±0.03b	1.60±0.055a
	T3	1.20±0.038a	1.14±0.127a	0.35±0.020bc	0.42±0.018a	1.59±0.06b	1.56±0.111a
	T4	0.98±0.050b	0.97±0.051ab	0.35±0.037bc	0.31±0.055b	1.34±0.01c	1.28±0.015b
10 mm·h^{-1}	CK	0.92±0.058c	0.79±0.101b	0.27±0.020c	0.26±0.027c	1.19±0.040c	1.05±0.075b
	T1	1.33±0.064a	1.21±0.079a	0.53±0.036a	0.51±0.031a	1.86±0.099a	1.72±0.071a
	T2	1.34±0.064ab	1.19±0.041a	0.40±0.003b	0.49±0.021a	1.75±0.067a	1.68±0.056a
	T3	1.14±0.020ab	1.16±0.113a	0.38±0.022b	0.44±0.026b	1.51±0.041b	1.57±0.139a
	T4	1.18±0.083b	1.15±0.050a	0.36±0.035b	0.37±0.019b	1.54±0.050b	1.53±0.037a

Data are shown as mean ±SE with the different lowercase letters in the same column represent significant differences ($P<0.05$).

3.4 Effects of simulated rainfall on chlorophyll fluorescence parameters of *C. album* after spraying mesotrione

3.4.1 The effect of 2 mm/h rain intensities on chlorophyll fluorescence parameters after spraying mesotrione

These two types of indicators Fv/Fm and Y(II) in the leaves showed a certain decline after using mesotrione. At the same time, all processing intervals were showing an upward trend. However, if the time between rains continued to extend, there was a possibility that there might be an overall decline between the two (Figure 2). The max value is T1 after the mesotrione, and the minimum will be T4. After taking relevant treatments, there is no significant difference in Fv/Fm indicators of other treatments compared with CK. But this does not include T1 treatment, T1 treatment caused a significant increase in Fv/Fm, with an amplitude of 27.65%.

In addition, it can be seen that there is no big difference between T4 interval treatment Y(II) and CK, and the remaining treatment Y(II) has a greatly increasment , whose amplitude are 43.85%, 36.97% and 35.60%, respectively. On the 4th day, if T1 treatment is not considered, the Fv/Fm of other treatments all show a certain increase. Compared with CK, the corresponding incidence rates are 66.54%, 46.62% and 23.42%, respectively; In the figure, the Y(II) treatment at T1 and T2 interval both increased to a certain extent, which increased by 210.88% and 127.98% respectively (Figure 2.A-B). In addition, after the use of mesotrione, the entire graph trend showed that Y (NPQ) would decrease with the extension of the rain interval, but the value of Y (NO) would continue to increase. If T1 was not considered at 4 DAT, the Y (NPQ) of T2 interval treatment would increase significantly, and there might not be much difference between other treatments and CK Y (NPQ); in addition, the Y (NO) index after T1 interval treatment had a certain drop of 10.38%. In contrast, there were not many differences between other treatments and CK (Figure 2.C-D).

Figure2: Effect of 2 mm/h rain intensity on chlorophyll fluorescence parameters of *C. album*

treated by mesotrione. CK and T1, T2, T3, T4 on the x-axis represent no rain and different rainfall interval times (0.5 h, 1 h, 2 h, and 4 h) respectively; y-axis represents chlorophyll fluorescence parameters. Diamonds, squares, triangles and circles represent different times after treatment (12 h, 24 h, 2 d and 4 d, respectively). A)the maximum photochemical quantum efficiency (Fv/Fm); B)the actual photochemical quantum yield Y(II); C)the regulated energy dissipation quantum yield Y(NPQ); D)the non-regulated energy dissipation quantum yield Y(NO) of *C. album* treated by mesotrione. Lowercase letters in the same column indicate significant differences at the 0.05 level.

3.4.2 Effect of 6 mm/h rain intensities on chlorophyll fluorescence parameters after spraying mesotrione

Based on the situation shown in Figure 2, if the rain interval continued to extend, the indicators Fv/Fm and Y(II) would both show a downward trend. In particular, if the application exceeds 12 hours, the effect of mesotrione will be greatly reduced due to rainfall. In addition, the Fv/Fm index values are much higher than in the CK case in all treatment intervals, and the corresponding growth rates are 42.47%, 33.27%, 24.97% and 17.96%, respectively. In addition, on the 4th day, the condition of the patients treated with the T4 interval will be relieved, and the Fv/Fm index will no longer be significantly different from that in the case of CK. It should also be noted that compared with the case of CK, the Fv/Fm of other treatments will be greatly improved, and the corresponding increases are 82.59%, 72.07% and 62.67% respectively (Figure 3.A).

Compared to the situation of CK, Y(II) of *C. album* had a increasment by 528.50% and 398.96% at 4 DAT for T1, and T2 treatments. And it is noted that it is almost the same as T3, T4 treatments and CK (Figure 3. B), stating that if the coppresponding rain intensity reached 6 mm/h, the rainfall within 1 hour could be give a great influence at the efficacy of mesotrione.

The Y(NPQ) value of *C. album* was measured at different periods, and its magnitude was as follows: 24 hours after treatment>12 hours after treatment>2 d after treatment>4 d after treatment.

The photosynthetic inhibitory effect of *C. album* was the strongest at 4 DAT. Compared with 12 hours after application, because of the self-protection mechanism of *C. album*, Y(NPQ) will has a slower increasment, and then slowly decreased; Y(NO) showed that with the extension of the interval and the passage of the reaction time gradually increase. Compared with CK at 24 hours after the mesotrione, Y(NPQ) in all interval treatments decreased significantly; Y(NO) in all interval treatments was significantly lower than CK, T1 and T2 treatments would not show much differences.(Figure 3.C-D)).

Figure3: Effect of 6 mm/h rain intensity on chlorophyll fluorescence parameters of *C. album* treated by mesotrione. CK and T1, T2, T3, T4 on the x-axis represent no rain and different rainfall interval times (0.5 h, 1 h, 2 h, and 4 h) respectively; y-axis represents chlorophyll fluorescence parameters. Diamonds, squares, triangles and circles represent different times after treatment (12 h, 24 h, 2 d and 4 d, respectively). A)the maximum photochemical quantum efficiency (Fv/Fm); B)the actual photochemical quantum yield Y(II); C)the regulated energy dissipation quantum yield Y(NPQ); D)the non-regulated energy dissipation quantum yield Y(NO) of *C. album* treated by mesotrione. Lowercase letters in the same column indicate significant differences at the 0.05 level.

3.4.3 The effect of 10 mm/h rain intensities on chlorophyll fluorescence parameters after spraying mesotrione

In addition, based on the display status of the graph, rainfall will increase the Fv/Fm and Y(II) values of *C. album* (Figure 3). However, this situation will be weakened by the extension of the rainfall time, and this trend will gradually flatten out. Compared with the results under CK conditions, Fv/Fm show the great increment by 25.14%~43.93% for all intervals at 12 h and 4 days after treatment. Ignoring the results from T4 treatment, others would demonstrate the better increment by 85.08% and 78.31%, 71.54%, which was much higher than CK, and T1, T2 and T3

treatment, Y(II) give a good growth from the observation, which means that the possible influence of mesotrione on *C. album* become much weaker when it is raining. If the rain intensity arrives at 10 mm/h, T1, T2 and T3 treatment had a much stronger restrictive effect mesotrione (Figure 4.A-B). Y(NPQ) will fluctuate when it suffers from rain intensity of 2 mm/h and 6 mm/h. It might have a growth at 24 h after treatment compared with 12 h, and then slowly declined and minimized after 4 d, except for the T4 treatment. T1, T2 and T3 treatments is possible to have an impact on the mesotrione of *C. album*. Compared with CK, Y(NPQ) gives a higher increment at 422.82%, 220.58%, and 199.11% (Figure 4.C).

Figure4: Effect of 10 mm/h rain intensity on chlorophyll fluorescence parameters of *C. album* treated by mesotrione. CK and T1, T2, T3, T4 on the x-axis represent no rain and different rainfall interval times (0.5 h, 1 h, 2 h, and 4 h) respectively; y-axis represents chlorophyll fluorescence parameters. Diamonds, squares, triangles and circles represent different times after treatment (12 h, 24 h, 2 d and 4 d, respectively). A)the maximum photochemical quantum efficiency (Fv/Fm); B)the actual photochemical quantum yield Y(II); C)the regulated energy dissipation quantum yield Y(NPQ); D)the non-regulated energy dissipation quantum yield Y(NO) of *C. album* treated by mesotrione. Lowercase letters in the same column indicate significant differences at the 0.05 level.

4 Discussion

With the continuous acceleration of the process of agricultural modernization and the gradual transfer of rural labor to cities, chemical control has become the dominant method in the field of weed control due to its high efficiency and practicality [29-30]. Temperature, humidity, light, wind, and precipitation will affect the effectiveness of the mesotrione [31-32]. Global warming causes the rainfall increases and the rainfall mainly is the short-duration heavy rainfall in summer, heavy monsoon rains would increase the risk of crop failure within regions or globally [33-34]. The slow-moving cold front delays the occurrence of peak rainfall but enhances the rainfall intensity, the

maximum hourly summer rainfall intensity has increased by about 11.2% on average [35]. The scouring effect of rain water reduces the effective components of the medicament attached to the leaf surface and reduces the herbicide effect [36].

Zhang *et al*.[37] reported that with the increase of the dose of mesotrione, the inhibitory effect on corn growth increased, mainly manifested as a significant decrease in plant height; Zhang *et al*. [22] and Wang *et al*. [23] reported spraying nitrate. After sulcotrione, the heart leaves of Amaranthus retroflexus were severely chlorosis and then the whole plant will died. The control efficiency of plant and fresh weight were high than common plants; Gao *et al*. [38] reported that the effect of mesotrione in the control of weeds increased with weed physiological metabolism intensified, the symptoms are more significant later. Spillman [39] think the rainfall would dilute or wash the medicament on weed's surface, and then the reduced efficacy couldn't have a thorough effect on the weed. In this study, on condition that the rain intensity was 2 mm/h and the rainfall interval was T1, the plant height inhibition rate of *C. album* was still close to that of no rainfall after application, indicating that a light rain after application would not affect the efficacy of mesotrione. When the rain intensity is 6 mm/h, under the treatment of 2 h interval, with the extension of the rain interval time, the control effect of mesotrione on *C. album* gradually increases, and the control effect of plant and fresh weight is still above 90%, it only decreased by 0.4%-2.47% compared with CK, the efficacy of mesotrione was not significantly affected.

The mechanism of action of herbicides is mainly to cause weed death by interfering or inhibiting the physiological metabolism of plants, including photosynthesis, fatty acid synthesis, pigment synthesis, amino acid metabolism [40]. HPPD is the only target enzyme for mesotrione plants, and carotenoids are synthesized products of the target enzyme (HPPD) after the catalytic reaction. Therefore, the changes in the mass fraction of photosynthetic pigments in the leaves can reflect the HPPD enzyme in *C. album*. The level of activity is used as an important indicator of the weed control efficiency of mesotrione on weed control [41]. Rainfall not only straightly affected the

efficacy of herbicide by changing the absorbing and conduction capabality inside the plants, but also by changing the growth and physiological property indirectly. Zhang *et al*. [42] found that the carotenoid, chlorophyll a and chlorophyll b content in the leaves of *C. album* gradually decreased with the time of the herbicide reaction after spraying mesotrione. Zhang *et al.* [43] thinks that the erosion resistance is better with the increase of retention on leaves. In this experiment, the control effect decreased after spraying mesotrione, may be due to rainfall washing the pesticide retention on the plant surface, which increased the photosynthetic pigment content of *C. album* leaves, but as the reaction time increased, the content of photosynthetic pigments decreased step by step. However, when the rainfall intensity increased, the content of carotenoids in leaves of *C. album* was significantly higher than that of the control when the rainfall intensity was 6 mm/h and the interval was within 1 h. If the rainfall intensity continued to add to 10 mm/h, the carotenoid content of leaves of *C. album* was still significantly increased after 2 h interval times, significant difference disappeared under the 4 h rainfall disposal. It is consistent with the conclusion which was the rainfall after 4 h didn't have effect on the efficacy of the new-style herbiside —Y11049 [44].

Chlorophyll fluorescence is a means of rapidly identifying injury to leaves in the absence of visible symptoms. This is a detailed analysis of causes of change in photosynthetic capacity. It has been widely used in laboratory studies in understanding the mechanism of photosynthesis and the mechanisms by which a range of external factors alter photosynthetic capacity. The measurement of chlorophyll fluorescence also has considerable potential for use in the field situation [45]. Fv/Fm represents the strength of photoinhibition after the plant is stressed, reflecting the maximum light energy conversion efficiency in the photosynthetic reaction center, and Y(II) refers to the actual light energy conversion efficiency of the plant photosynthesis process [46]. Y(NPQ) is one of the self-protection indicators for plants to convert excess light energy into heat and the degree of light damage to plants is represented by Y(NO) [47-48]. Yang *et al*. [49] found that 2,4-D treatment significantly reduced the Fv/Fm and Y(II) of Jinfen No.107 and Jingu No.29 millet seedling leaves.

Guo *et al*. [50] reported that after spraying chipton, the Fv/Fm of millet has an increasing inhibitory effect as the increased of the spraying concentration. We found that Fv/Fm, Y(II), Y(NPQ) of *C. album* leaf gradually decreased with the extension of the interval, Y(NO) showed an upward trend conversely. We had tested the Y(NPQ) of the *C. album* leaf after spraying mesotrione for 24 h, it was higher than that of 12 h, indicating that the *C. album* can relieve the caused by mesotrione to a certain extent by increasing heat dissipation after being slightly stressed. Photodamage, the photochemical reaction efficiency continues to decline due to the serious herbicide stress, and it shows a downward trend as the rain interval increases. Therefore, the efficacy of mesotrione has weakened, which is mainly due to the increase in leaf photosynthetic pigment content, and the increase of photosynthetic capacity of photosystem II, which affects the control effect of mesotrione and the growth of *C. album* and photosynthesis. *C. album*'s growth difference under different disposal is directly connected with the absorption and conduction of mesotrione, it needs further discussion about the loss of the efficacy and the residual on the surface of leaves.

When the rainfall intensity was 6 mm/h within 1 h after mesotrione spraying, or the rainfall intensity was 10 mm/h within 2 h, the loss of plant control effect was above 11%. Oils adjuvants could reduce the surface tension and contact angle of the herbicide liquid, then the herbicide would have better wettability, adhesiveness, penetrating quality and resistance to rain washing capability and ultimately the efficacy would be increased [51]. Tao *et.al* [52] researched that adjuvants could increase the prevention and control effect if the rainfall appears in 4 h after mesotrione was applied. Terrible environment would reduce herbicide's efficacy, but effective ingredient in the adjuvants could increase herbicide's prevention and control effect by counteracting the influence. And how to choose adjuvants needs to be further studied.

5 Conclusion

In conclusion, rainfall reduced the control effect of mesotrione on *C. album*, one of the reasons

was the increase of photosynthetic pigment content and the enhancement of photosystem II activity. The 2 mm/h not significantly effected the control effect of mesotrione; In the circumstance of 6 mm/h of the rainfall intensity, the control effect of mesotrione was significantly reduced within 0.5 h and 1 h, the fresh weight control efficacy decreased by 16.90% and 7.14%, respectively; and in the 10 mm/h of the rainfall intensity, the plant control efficacy was reduced by 28.48% and 18.42%, respectively, even the interval of rainfall was 2 h, it still showed a reduction rate of 14.78 percent.

Acknowledgments

We would express sincere appreciation to two anonymous reviewers for their thoughtful and valuable comments, which helped us to improve this manuscript. Thanks are also due to Professor X.Y. Yuan of Shanxi Agricultural University for suggestions on the manuscript.

Authors' contribution

The author would like to thank all authors for their help in the experience and manuscript. Mengmeng Sun was responsible for preparing the final version of manuscript. Xiangyang Yuan helped conceive the experiments and was the project director. Meijun Guo and Shuqi Dong were involved in review the language of the manuscript. Xie Song and helped analysis and interpretation of data. Shuai Guo and Yanfen Li were involved in literature collection. Xiaoxin Shi was involved the meteorological datas collection and calculation.

References

[1] Das TK, Yaduraju NT. The effects of combining modified sowing methods with herbicide mixtures on weed interference in wheat crops. International Journal of Pest Management, 2012; 58(4): 310-319. doi: 10.1080/09670874.2012.680933

[2] Li XJ. Main problems and management strategies of weeds in agricultural fields in China in recent years. Plant Protection, 2018; 44(5): 77-84. doi: 0.16688/j.zwbh.2018322 (in Chinese)

[3] Poonia, A, Upadhayay, A. *Chenopodium album* Linn: review of nutritive value and biological properties. Journal of Food Science and Technology volume, 2015; 52(7): 3977–3985. doi: 10.1007/s13197-014-1553-x

[4] Gao YY. Research on *Chenopodium* in ancient southwest China. IOP Conference Series Earth and Environmental Science, 2021; 621: 012124. doi: 10.1088/1755-1315/621/1/012124

[5] Manila B, Surendra S, Saurabh P, Bhumesh K. A pharmacological comprehensive review on *Chenopodium album* L. International Journal of Current Research, 2020; 12(12): 15360-15368. doi: 10.24941/ijcr.40425.12.2020

[6] Vartika S, Sweta G, Hema S, Akhilesh SR. Ecophysiological characteristics of five weeds and a wheat crop in the Indo-Gangetic Plains, India. Weed Biology and Management, 2015; 15(3):102-112. doi: 10.1111/wbm.12073

[7] Bajwa AA, Zulfiqar U, Sadia S, Bhowmik P, Chauhan BS. A global perspective on the biology, impact and management of *Chenopodium album* and *Chenopodium murale*: two troublesome agricultural and environmental weeds. Environmental Science and Pollution Research, 2019; 26: 5357–5371. doi: 10.1007/s11356-018-04104-y

[8] Conley SP, Stoltenberg DE, Boerboom CM, Larry KB. Predicting soybean yield loss in giant foxtail (Setaria faberi) and common lambsquarters (*Chenopodium album*) communities. Weed Science, 2003; 51(3): 402-407. doi: 10.1614/0043-1745(2003)051[0402:PSYLIG]2.0.CO;2

[9] Tehmina A, Rukhsana B. Competition Losses Caused by *Rumex dentatus* L. and *Chenopodium album* L. in Wheat (*Triticum aestivum* L.). The Philippine Agricultural Scientist, 2010; 93(3):

[10] Sarabi V, Mahallati MN, Nezami A, Mohassel MHR. Effects of the relative time of emergence and the density of common lambsquarters (*Chenopodium album*) on corn (*Zea mays*) yield. Weed Biology and Management, 2011; 11(3):127-136. doi: 10.1111/j.1445-6664.2011.00414.x

[11] Yang HH, Wang LQ, Xie ZJ, Yu Q, Liu G, Tan HR. The tyrosine degradation gene hppD is transcriptionally activated by HpdA and repressed by HpdR in Streptomyces coelicolor, while

hpdA is negatively autoregulated and repressed by HpdR. Molecular Microbiology, 2007; 65(4): 1064-1077. doi: 10.1111/j.1365-2958.2007.05848.x
[12] Guo WL, Feng L, CuiY, Wu DD, Zhang TJ, Zhang C, et al. Weed control effect of 30% nicosulfuro•atrazine•mesotrione oil dispersion and the safety to sweet corn in field. Journal of Maize Sciences, 2020; 28(4): 178-183. doi: 10.13597/j.cnki.maize.science.20200426 (in Chinese)
[13] Chen HY, Deng GQ, Fu HY, She YB. Advances in detection of systemic pesticide residues in agricultural products. Chemical Reagents, 2020; 42(10): 1139-1147. doi: 10.13822/j.cnki.hxsj.2020007659 (in Chinese)
[14] Tao B. Practical techniques for chemical control of weeds. 1st ed. Beijing: Chemical Industry Press, 2013; 255.
[15] Wu JY, Mathiassen S, Kudsk P. The influence of weed species, leaf stage and rainfastness on the performance of nicosulfuron. Chinese Journal of Pesticide Science, 2003(1): 77-81. (in Chinese)
[16] Luo ZR, Fu Q, Xie ZB. Effect of simulated rainfall on the weeding effect of paraquat. Journal of Fruit Science, 1995(2): 104-106. doi: 10.13925/j.cnki.gsxb.1995.02.011 (in Chinese)
[17] Wang CF, You Y, Wang J, Wu JY, Chen F, Wang QH. Effect of turf-mowing and simulated rainfall on the efficiency of bensulfuron-methyl•metsulfuron-methyl. Journal of Fujian Agriculture and Forestry University(Natural Science Edition), 2009; 38(4): 351-355. (in Chinese)
[18] Wang QH, Wu JY, Jiang GQ. Effects of rainfall and adjuvants on phytotoxicity of imazethapyr. Chinese Journal of Pesticide Science, 2003(3): 55-58. (in Chinese)
[19] Gao S, Zhang ZJ, An WL, Lin CF, Yang YT, Ma HJ, et al. Biological activity and safety of herbicide mesotrione in corn field. Agrochemicals, 2004(10): 469-471. doi: 10.16820/j.cnki.1006-0413.2004.10.015 (in Chinese)
[20] Zhang Y, Lv AQ, Wu CX, Lu XT, Ma C. Controlling weeds with mesotrione 15% SC in bluegrass lawn. Journal of Weed Science, 2013; 31(4): 54-56. doi:

0.19588/j.issn.1003-935x.2013.04.018 (in Chinese)

[21] Wang Z, Yao AQ. Mesotrione bioactivity and selectivity to corn. Journal of Weed Science, 2014; 32(2): 59-61. doi: 10.19588/j.issn.1003-935x.2014.02.017 (in Chinese)

[22] Vranjes F, Vrbnicanin S, Nedeljkovic D, Savic A, Bozic D. The response of Chenopodium album L. and Abutilon theophrasti Medik. to reduced doses of mesotrione. Journal of Environmental ence and Health Part B, 2019; 54(7):1-7. doi: 10.1080/03601234.2019.1616980

[23] Dayan FE, Zaccaro MDM. Chlorophyll fluorescence as a marker for herbicide mechanisms of action. Pesticide Biochemistry and Physiology, 2012; 102(3):189-197. doi: 10.1016/j.pestbp.2012.01.005

[24] Hao ZC, Wang H, Dunzhu JC, Ju Q. Spatiotemporal variations of soil moisture across Shanxi province during 2003-2012. Acta Scientiarum Naturalium Universitatis Sunyatseni, 2014; 53(5):134-141. doi: 10.13471/j.cnki.acta.snus.2014.05.047 (in Chinese)

[25] Li DK, Zou YX, Ma XZ, Zhang FZ, Dong P, La YP. Field efficacy and rain resistance of fungicides against potato late blight. China Plant Protection, 2012; 32(5): 50-52+59. (in Chinese)

[26] Cui HL, Jia F, Quan ZH, Cui HL, Li XJ. Effects of glyphosate on weed control, soybean safety and weed occurrence in transgenic herbicide-resistant soybean. Scientia Agricultura Sinica. 2020; 53(6): 1166-1177. (in Chinese)

[27] Gao JF. Plant Physiology Experiment Guide. 1st ed. Beijing: Higher Education Press, 2006.

[28] Lesleigh F, Christa C, Jack JS. New fluorescence parameters for monitoring photosynthesis in plants. Photosynthesis Research, 2003; 78(1): 17-33. doi: 10.1023/A:1026012116709

[29] Mark AP, Alberto C, Ramiro O, Vinod S, Michael JW. The challenge of herbicide resistance around the world: a current summary. Pest Management Science, 2018; 74(10):2246-2259. doi: 10.1002/ps.4821

[30] Zhao YN, Zi XJ, Wang W, Feng Y, Yang P, Wang PK, Gao XL. Control effects of different herbicides on weeds as well as their effects on growth and development of broomcorn millet.

Chinese Journal of Applied Ecology, 2020; 31(7): 2415-2421. doi: 10.13287/j.1001-9332.202007.027 (in Chinese)

[31] Prashant J, Vipan K, Josefina G, Nicholas R. Tank mixing pendimethalin with pyroxasulfone and chloroacetamide herbicides enhances in-season residual weed control in corn. Weed Technology, 2015; 29(2):198-206. doi: 10.1614/WT-D-14-00095.1

[32] Bradley CJ, Bryan G. Influence of temperature and relative humidity on the foliar activity of mesotrione. Weed Science, 2002; 50(2): 157-161. doi: 10.1614/0043-1745(2002)050[0157:IOTARH]2.0.CO;2

[33] Yang P, Ren GY, Hou W, Liu WD. Spatial and diurnal characteristics of summer rainfall over Beijing Municipality based on a high-density AWS dataset. International Journal of Climatology, 2013; 33(13): 2769-2780. doi: 10.1002/joc.3622

[34] Beillouin D, Schauberger B, Bastos A, Ciais P, Makowski D. Impact of extreme weather conditions on European crop production in 2018. Philosophical Transactions of the Royal Society B: Biological Sciences, 2020; 375:20190510. doi: 10.1098/rstb.2019.0510

[35] Xiao C, Wu PL, Zhang LX, Song LC. Robust increase in extreme summer rainfall intensity during the past four decades observed in China. Scientific Reports, 2016; 6: 38506. doi: 10.1038/srep38506

[36] Tim RH, Michael P L, Russell V, Rachael EB, Fiona MC, Elaine AM, et al. Characterization of 4-hydroxyphenylpyruvate dioxygenases, inhibition by herbicides and engineering for herbicide tolerance in crops. Pesticide Biochemistry and Physiology, 2019; 156: 9-28. doi: 0.1016/j.pestbp.2019.01.006

[37] Zhang HJ, Liu X, Ni HW, Li XJ, Cui HL. Early diagnosis and mitigation methods of mesotrione on maize. Journal of Weed Science, 2009(4): 19-22. doi: 10.19588/j.issn.1003-935x.2009.04.005 (in Chinese)

[38] Gao XX, Li M, Fang F, Yu TC, Liu XT. Interaction of clopyralid and mesotrione in maize field.

Journal of Maize Sciences, 2015; 23(3): 143-148. doi: 10.13597/j.cnki.maize.science.20150324 (in Chinese)

[39] Spillman JJ. Spray impaction, retention and adhesion: An introduction to basic characteristics. Pest Management Science, 1984: 15(2): 97-106. doi: 10.1002/ps.2780150202

[40] Guo L, Zhang BB, Shen JH, He X, Song HF. Effects of glyphosate and paraquat on root morphology and aboveground growth of prunus persica seedlings. Chinese Journal of Applied Ecology, 2020; 31(2): 524-532. doi: 10.13287/j.1001-9332.202002.036 (in Chinese)

[41] Mamiko S, Takahiro H, Yoshio S, Kangetsu H, Shinichi B. Action mechanism of bleaching herbicide cyclopyrimorate, a novel homogentisate solanesyltransferase inhibitor. Journal of Pesticide Science. 2018; 43(4):233-239. doi: 10.1584/jpestics.D18-008

[42] Zhang FW, Yao XF, Sun SA, Wang LP, Liu WT, Jiang XY, et al. Effects of mesotrione on oxidative stress, subcellular structure, and membrane integrity in Chlorella vulgaris. Chemosphere, 2020(247): 125668. doi: 10.1016/j.chemosphere.2019.125668

[43] Zhang P, Jiang XY, Tan HL, Shang DL, Li XD, Zhang JW. Effects of four spray adjuvants on the rainfastness of azoxystrobin on maize leaf surfaces and their effect on maize safety. Chinese Journal of Pesticide Science, 2018; 20(2): 239-248. doi: 10.16801/j.issn.1008-7303.2018.0032

[44] Lin FR, Huang XH, Chang H, Chen J. Herbicidal characteristics of a novel protoporphyrinogen oxidase inhibitor-Y11049. Chinese Journal of Pesticide Science, 2021; Forthcoming. doi: 10.16801/j.issn.1008-7303.2021.0117 (in Chinese)

[45] Sascha BS, Sebastiaan AM, Harm GG, Petra B, Dick Z, Dick V, et al. Laboratory algal bioassays using PAM fluorometry: Effects of test conditions on the determination of herbicide and field sample toxicity. Environmental Toxicology and Chemistry, 2014; 33: 1017-1022. doi: 10.1002/etc.2537

[46] Shao GQ, Li ZJ, Ning TY, Zheng YH. Responses of photo-synthesis, chlorophyll fluorescence,

and grain yield of maize to controlled release urea and irrigation after anthesis. Journal of Plant Nutrition and Soil Science, 2013; 176: 595-602. doi: 10.1002/jpln.201100185

[47] Tsai YC, Chen KC, Cheng TS, Lee C, Lin SH, Tung CW. Chlorophyll fluorescence analysis in diverse rice varieties reveals the positive correlation between the seedlings salt tolerance and photosynthetic efficiency. BMC Plant Biology, 2019; 19(1):403. doi: 10.1186/s12870-019-1983-8

[48] Yang HJ, Yuan XY, Guo PY, Dong SQ, Zhang LG, Wen YY, et al. Effects of brassinolide on photosynthesis, chlorophyll fluorescence characteristics and carbohydrates metabolism in leaves of foxtail millet under Sigma broad stress. Scientia Agricultura Sinica, 2017, 50(13): 2508-2518. (in Chinese)

[49] Yang XF, Sun P, Sun DS, Song XE, Dong SQ, Yuan XY, et al. Safety evaluation of allelochemical derivative pyrone on different millet varieties. Chinese Journal of Applied Ecology, 2020, 1(7): 2236-2242. doi: 10.13287/j.1001-9332.202007.005 (in Chinese)

[50] Guo M J, Bai YQ, Gao P, Shen J, Dong SQ, Yuan XY, et al. Effect of MCPA on leaf senescence and endogenous hormones content in leaves of foxtail millet seedlings. Scientia Agricultura Sinica, 2020, 53(3): 513-526. (in Chinese)

[51] Li JJ, Zhang YH, Wang BR, Wang LC, Tao B. Synergistic Effect of Different Adjuvants on Atrazine in Maize Field. Journal of Maize Sciences, 2019; 27(6): 167-174. doi: 10.13597/j.cnki.maize.science.20190626 (in Chinese)

[52] Tao B, Zhang YH, Tian LJ, Zheng CY, Li SY, Kong LW. Effects of adjuvants on spray droplet distribution of herbicides sprayed by plant protection UAV. Plant Protection, 2021; 47(1): 108-116. doi: 10.16688/j.zwbh.2019558 (in Chinese)

Figure1: Effect of simulated rainfall on carotenoid content in leaves of C. album treated by mesotrione after 3(A) and 6(B) DAT.

Click here to access/download;Figure;Figure 1.jpg

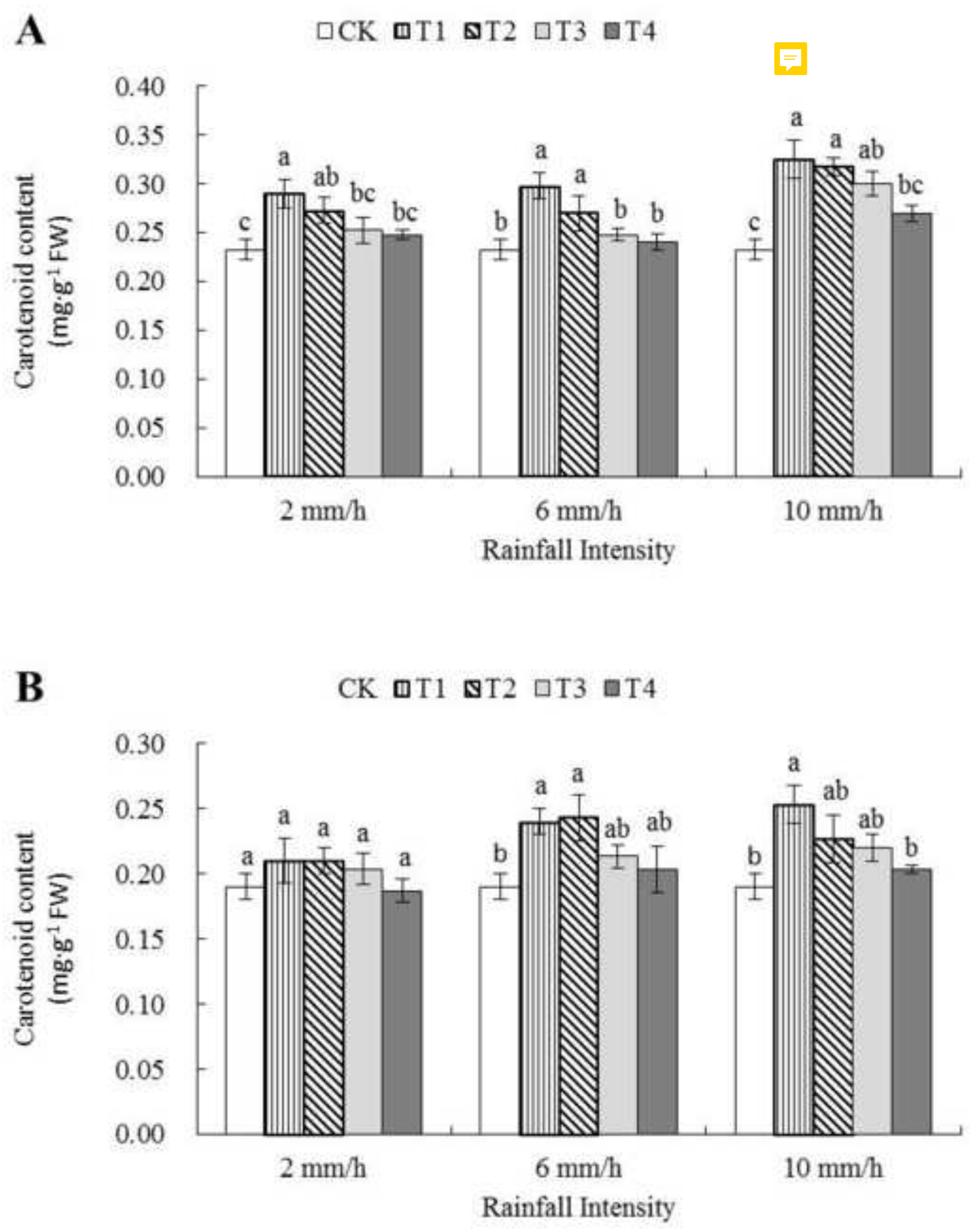

Figure2: Effect of 2 mm/h rain intensity on chlorophyll fluorescence parameters of C. album treated by mesotrione.

Click here to access/download;Figure;Figure 2.jpg

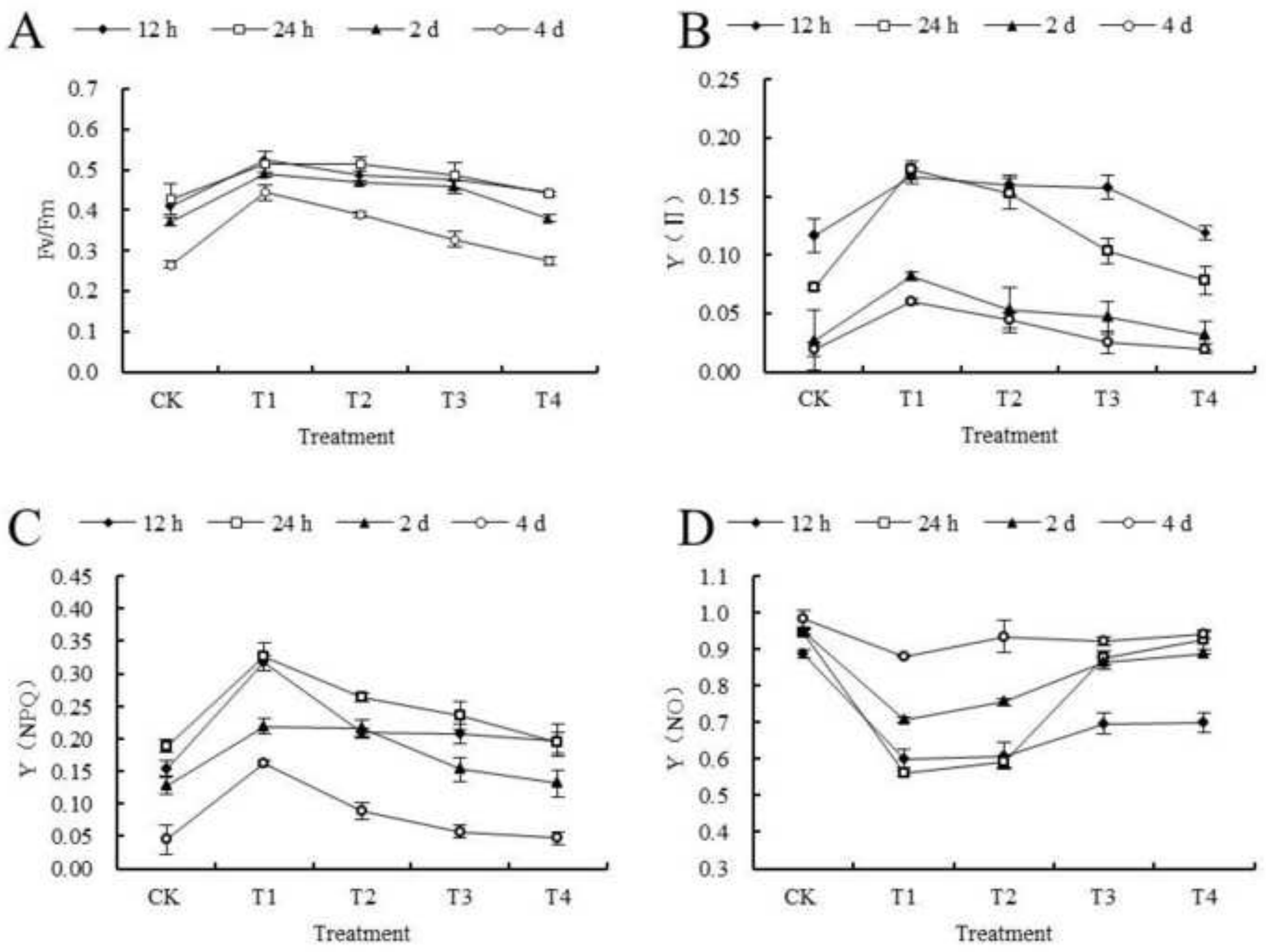

Effect of 6 mm/h rain intensity on chlorophyll fluorescence parameters of C. album treated by mesotrione.

Click here to access/download;Figure;Figure 3.jpg

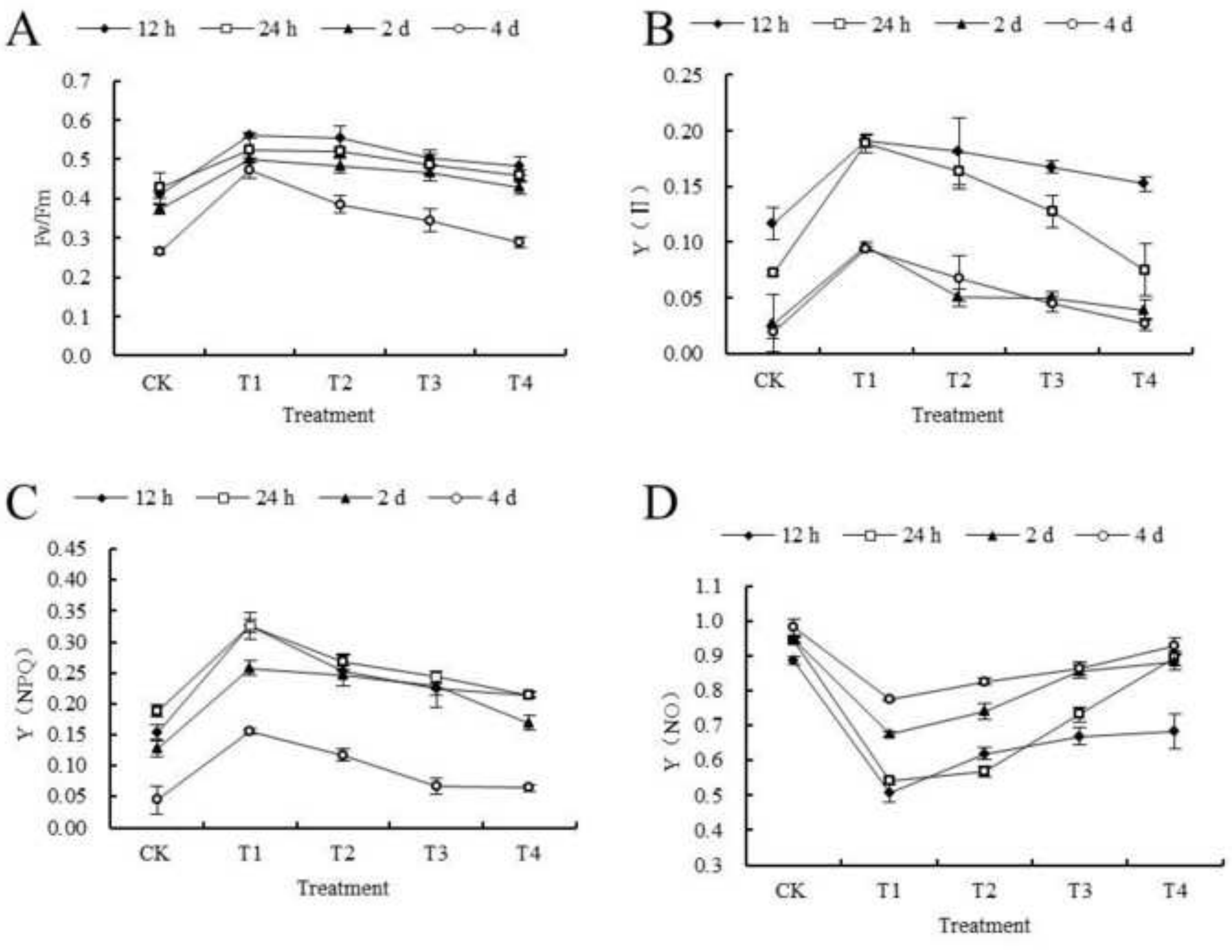

Effect of 10 mm/h rain intensity on chlorophyll fluorescence parameters of C. album treated by mesotrione.

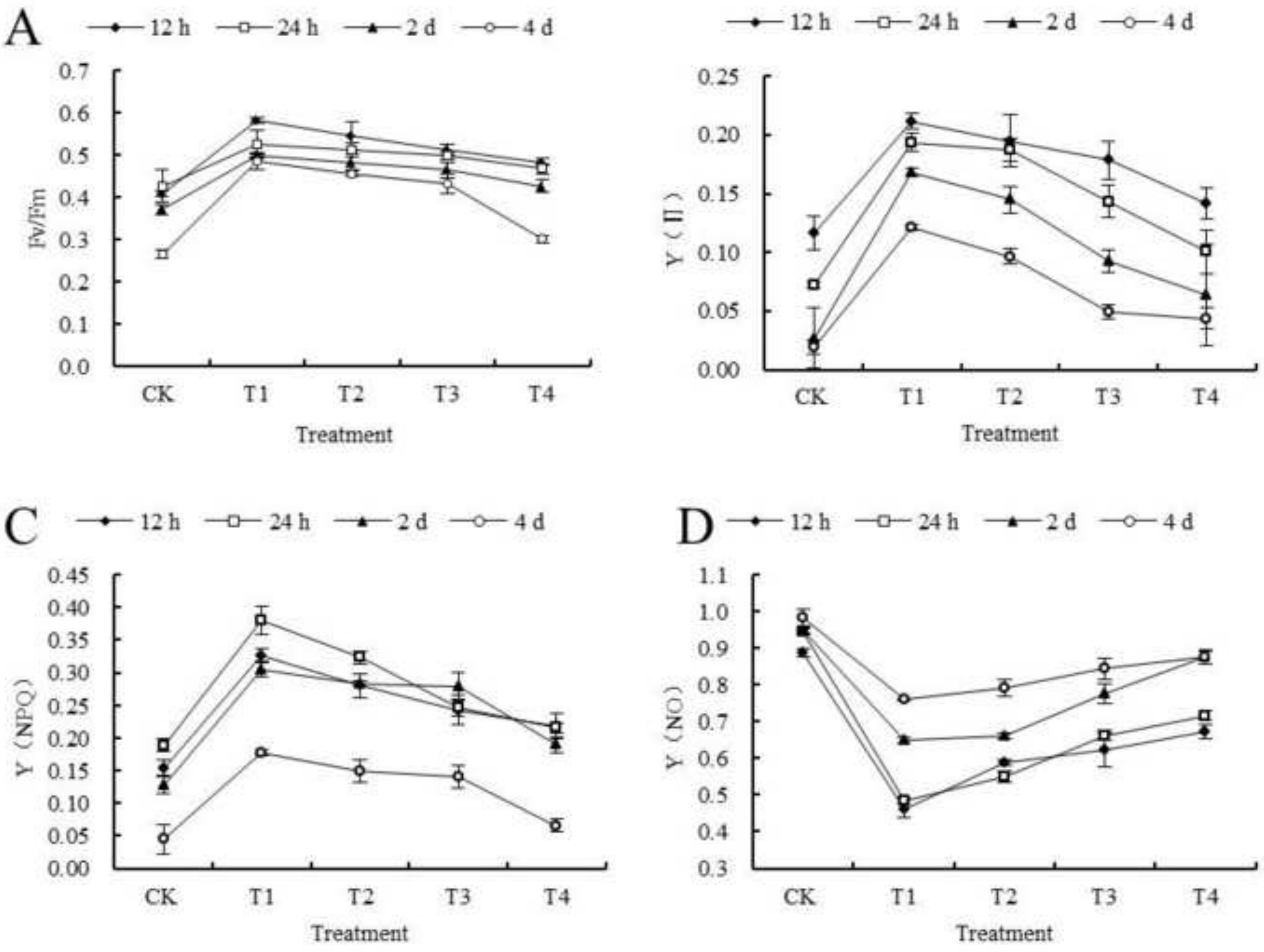

Supporting Information files

Click here to access/download
Supporting Information
Figure 1.xlsx

Revised Manuscript with Track Changes

Effects of mesotrione on the control efficiency and chlorophyll fluorescence parameters of *Chenopodium album* under simulated rainfall conditions

Mengmeng Sun[1#], Meijun Guo[2#], Shuai Guo[1], Yanfen Li[1], Shuqi Dong[1], Xie Song[1], Xiaoxin Shi[3], Xiangyang Yuan[1*]

[1] Laboratory of Crop Chemical Regulation and Chemical Weed Control, College of Agronomy, Shanxi Agricultural University, Taigu, Shanxi, China

[2] Department of biological science and technology, Jinzhong University, Jinzhong, Shanxi, China

[3] Taigu County Meteorological Bureau, Shanxi, China

*Corresponding author's E-mail: yuanxiangyang200@163.com(YXY)

[#]These authors contributed equally to this work. SM and GM are co-first authors on this work.

Style Definition: Heading 1: Line spacing: Double

Formatted: Line spacing: Double

Formatted: Justified, Line spacing: Double

Formatted: Line spacing: Double

Formatted: Superscript

Formatted: Justified, Line spacing: Double

Formatted: Superscript

Formatted: Superscript

Formatted: Line spacing: Double

Formatted: Default Paragraph Font, Font: (Default) +Body (Calibri), 10.5 pt

Abstract:

This experiment was conducted to study the effects of mesotrione on the control efficiency and chlorophyll fluorescence parameters of *Chenopodium album*. Simulating three rainfall intensities of 2 mm/h (light rain), 6 mm/h (moderate rain) and 10 mm/h (heavy rain) at different interval times (0.5 h, 1 h, 2 h, 4 h)to analyze variable regulation of the control effect, the photosynthetic pigment content and chlorophyll fluorescence~~nce~~ parameters of ~~*Chenopodium*~~ *C. album* after spraying mesotrione. ~~The control efficiency of mesotrione on *Chenopodium* was decreased after rainfall, but the effect of different interval treatments on *Chenopodium* was different under different rainfall intensities.~~ With the extension of rainfall time interval~~, T~~, the inhibition rate of plant height, plant control effect and fresh weight control effect of *C. album*~~*Chenopodium*~~ were gradually increased, the inhibition effect of rainfall on the efficacy was gradually decreased, at the same time, the contents of chlorophyll a, chlorophyll b, carotenoids, the maximum photochemical quantum efficiency (Fv/Fm), the actual photochemical quantum yield (Y(II)) and quantum yield(Y(NO)) production of regular energy consumption of ~~*Chenopodium*~~*C. album* were also increased, while the nonregulatory energy decreased gradually. The results showed that the contents of chlorophyll a and chlorophyll b in leaves of ~~*Chenopodium*~~*C. album* increased significantly by 35.63% and 35.38% compared with the control under the condition of simulating 6 mm/h in interval 1 hour. The study suggested that simulating 10 mm/h rainfall intensity had the greatest effect on ~~*Chenopodium*~~*C. album*, the photosynthetic pigment content, Fv/Fm and Y(II) of leaves were significantly higher than those in the control groups under 0.5 h, 1 h and 2 h interval treatments. The carotenoid content was the lowest and Y(NO) was the largest under the 4 h interval treatment. ~~At this time, the control effect of mesotrione on *Chenopodium* was for maximum benefits.~~ As is displayed that rainfall reduced the weed~~ing~~ control effect in the aspect of controlling ~~*Chenopodium*~~*C. album* ~~with~~ on mesotrione, which is partly contributed to increase photosynthetic pigment content and enhance the

Formatted: Font: 18 pt, Font color: Auto

Formatted: Font: 18 pt, Not Bold, Font color: Auto

Formatted: Heading 1, Line spacing: single

Formatted: Font: 18 pt, Font color: Auto

Formatted: Indent: First line: 2 ch, Line spacing: Double

PS II photochemical activity. In conclusion, the rain intensity of ≤2 mm/h did not affect the control effect of mesotrione on ~~Chenopodium~~*C. album*. At 6 mm/h within 1 h after treatment, the control effect of fresh weight was significantly reduced by more than 7.14%, and at 10 mm/h within 2 h, the control effect was significantly reduced by more than 14.78%.

Keywords: Mesotrione; ~~Chenopodium~~*Chenopodium album*; Rainfall; Weed Control effect; Chlorophyll fluorescence

1 Introduction

~~As we all know,~~ Weed would reduce the yield and quality of the crops, hinder mechanized sowing and harvest and then slow down the process of agricultural modernization ~~weed will reduce the yield and quality of crops, hinder mechanized sowing and harvesting, which slow down the pace of China's agricultural modernization~~ [1-2]. *Chenopodium album*. L is an erect annual herbaceous plant, that grows between 0.2 and 2 meters in height, striped green, red or purple stems. Leaves are rhomboid, deltoid to lanceolate, upper entire, lower toothed or irregularly lobed. The leaves are wax-coated, sometimes has mealy and unregular sawtooth on the edge of them, with a whitish coat on the underside. The length of its petiole is close to blade or half of the blade [3]. ~~Chenopodium~~*C. album* is one of the common farmland malignant weed in northern China. It has strong environmental adaptability and a large root system, even grow on the Tibetan Plateau and in the lowlands of southwest China, so the *C. album* is recorded as a potherb in both plateaus and low lands [4]. It has obvious advantages when competing with crops for water and fertilizer, every single plant could yield 3000-20000 seeds (Data from CABI), which led to a sharp decline in crop yield [5-6~~3~~]. Sugarbeet, barley, mustard, gram, maize and so many crops were defeated by C. album, it has been reported to reduce the soybean (Glycine max) yield by 61%, in the wheat (Triticum aestivum) field, the yield loss approximately 50-60%, which also was associated with interference of this weed [7-9]. When the density increases to 20 plants per m^2, the corn would not ear because

Formatted: Font: (Default) Times New Roman

Formatted: Line spacing: Double

Formatted: Heading 1, Line spacing: single

Formatted: Line spacing: Double

Formatted: Font: Not Italic

Formatted: Font: 12 pt

Formatted: Superscript

of the shade effect of *C. album* [10].

Mesotrione ~~–~~is a herbicide of broad-spectrum, selective stem and leaf before bud and after seedling mainly used to effectively control broad-leaved weeds and some gramineous weeds by inhibiting the catalytic factors of plant photosynthetic process. Mesotrione is widely used in weed control of maize and winter wheat due to its high activity, low residue, strong compatibility and safety to the environment and subsequent crops [~~4~~11-~~5~~12]. Summer is the key period for crop growth and weeding. However, local microclimate and severe convective weather occur frequently, and rainfall interfere the herbicide efficacy due to the scouring every year [~~6~~13]. Therefore, it is attached importance to study the influence of rainfall on herbicides. There are great differences in the absorption rate and rain resistance among different stem and leaf treatment agents, ~~and~~ so the interval time of affecting the efficacy is different, but most herbicides will not affect the weed when rainfall after 4-6 h [~~7~~14]. Wu *et al*. [~~8~~15] found that rainfall after the application had a significant inhibitory effect on the efficacy of nicosulfuron methyl; paraquat could effectively control *Alopecurus aequalis* after 0.5 h and 1 h rainfall ~~interval treatments~~[16]; there was no significant difference in bensulfuron efficacy between without rainfall and after 2 h rainfall [~~10~~17]; Wang *et al*. [~~11~~18] showed that the effect of imazethapyr was no longer affected under the 9 mm/h rainfall intensity. When the interval time of rainfall is 0.5 h, the effect of rainfall on barnyardgrass was significantly reduced, it’s down to 19.42% [~~12~~19]. At present, it is known that mesotrione has effects on the growth of weeds and crops, such as reducing the plant height of maize, making *Amaranthus retroflexus* heart leaves be yellow, reducing the photosynthetic pigment content, reducing the maximum photochemical quantum efficiency (Fv/Fm) and the actual photochemical quantum yield Y(II) [~~13~~20-~~15~~22]. The inhibition rate of plant height, plant control effect and fresh weight control effect can well reflect the control effect of herbicides on weeds; photosynthetic pigment content reflects the green extent of plants; chlorophyll fluorescence parameters as a probe of photosynthetic capacity of leaves can reflect the status of weeds under herbicide stress [~~16~~23].

Most of the studies on mesotrione focused on its control effect and crop safety. There were no reports on the control effect and chlorophyll fluorescence parameters of mesotrione on broad-leaved weed ~~*Chenopodium*~~*C. album* under rainfall conditions. In this experiment, artificial rainfall was used to simulate rainfall, and ~~*Chenopodium*~~*C. album* is a common malignant weed in the field, was used to simulate different rain intensities at different intervals after spraying mesotrione.

In order to assess the possibility of mesotrione application and understand the related mechanism, we investigated the weed control, agronomic traits, photosynthetic pigment content and the chlorophyll fluorescence parameters of ~~*Chenopodium*~~*C. album* to mesotrione in this current study. The influence of rain intensity and interval time on the efficacy was determined, which provide a theoretical basis for the use of mesotrione.

~~1~~ 2 Materials and methods

~~1~~2.1 Experimental site

Field experiments were conducted at farming station of Shanxi Agricultural University during ~~July to August~~ 2019~~,~~ , Jinzhong, China (37.43°N, 112.61°E). The area is located in the northeast of Jinzhong region and belongs to a warm temperate continental monsoon climatic with an altitude of approximately 800 meters, the annual average temperature was 9.9 ℃, and the frost-free period was about 159 days. Annual precipitation of the province was 400-650 mm, but the seasonal distribution was uneven, with more than 60% of precipitation concentrated in June to August [~~17~~24].

Tables 1 and 2 present the meteorological conditions and soil characteristics of experimental site.

Formatted: Line spacing: Double

Formatted: Heading 1, Line spacing: single

Formatted: Font: Not Bold

Formatted: Font: (Default) Times New Roman

Formatted: Justified, Indent: First line: 2 ch, Line spacing: Double

Formatted: Font: 12 pt, Not Bold

Table 1. Meteorological data of the experimental sites during May-September in 2019

Month	Precipitation(mm)	The max rainfall intensity(mm/h)	Temperature(℃)			≥20℃accumulated temperature(℃)	Sunshine hours(h)
			Average	Min	Max		
5	1	0.8	19.9	35.3	2.6	235.1	258.9
6	55.7	9.3	24.5	35.6	12.4	715	213.4
7	65.1	13.4	25.4	37.1	10.8	788.4	243.1
8	52	5.2	22.9	34.5	9.7	614.2	219.6
9	78.8	6	18.4	34.1	6.6	195.5	223.9

Formatted: Justified

Formatted: Font: Bold

Formatted: Font: Bold

Table 2. The basic physical and chemical properties of soil

Depth of soil layer	pH	Available potassium(mg/kg)	Available phosphorus(mg/kg)	Available nitrogen(mg/kg)	organic matter(g/kg)
0-5 cm	8.21	484.21	43.70	64.20	8.52
5-10 cm	8.14	471.93	26.79	42.80	8.52
10-15 cm	8.17	319.60	21.28	49.93	7.99

~~1~~2.2 Experimental design

Four rainfall intervals were set: 0.5 h (T1), 1 h (T2), 2 h (T3), 4 h (T4), and no rainfall after mesotrione application was taken as control (CK). The completely randomized design was conducted by the field experiment with three replicates in 4 m^2 (2 m × 2 m)~~-~~. The recommended dose (~~200 ml/667 m^2~~ 3 L/ha hm^2) of 10% mesotrione emulsifiable concentrate (EC) (Shandong Shengbanglvye Chemical Limited Company) was sprayed by the ~~power operated sprayer.~~watering can.

~~1~~2.3 Rainfall designs

According to the standard of China Meteorological Administration(CMA): the hourly rainfall is 0.1～2.5 mm/h for light rain, 2.6～8 mm for moderate rain, and 8.1～15.9 mm for heavy rain. Simulating light rain 2 mm/h, moderate rain 6 mm/h and heavy rain 10 mm/h by power operated~~electric~~ sprayers (Lufeng, 3 WBD-20 C, 20 L). ~~Artificial spraying was carried out by simulating natural rainfall,~~ According to the calculation method that 1 L of rainfall per square meter is 1 mm [~~18~~25], artificial spraying was carried out by simulating natural rainfall, and the height of the spray is 0.5 meters. ~~by~~ By adjusting the knob on the edge of the ~~electric~~ sprayer, 4 L, 12 L, and 20 L of water are sprayed out within half an hour to obtain~~ 2 mm/h~~ three rain intensities of ~~-~~2 mm/h, 6 mm/h and 10 mm/h. Before the experiment, several tests were carried out with the

Formatted: Justified

Formatted: Font: Bold

Formatted: Font: Bold

Formatted: Indent: First line: 0 ch, Line spacing: Double

Formatted: Font: (Default) Times New Roman

Formatted: Heading 2, Line spacing: single

Formatted: Line spacing: Double

Formatted: Font: (Default) Times New Roman

Formatted: Heading 2, Line spacing: single

meteorological rain gauge~~,~~. Firstly, we screened out one type of shower that sprayed the smallest water and most homogeneous when spraying, ~~and~~ then the positions of different rain intensities on the sprayer knob were accurately marked. The process was repeated until the desired rainfall amounts and intensities were achieved.

To prevent drift effects in field conditions, we put~~ed~~ many baffles around the rainfall simulation areas of the neighborhood. The simulated rainfall is carried out under calm or breezy weather to reduce the error, and the test rain intensity is determined again every time the simulated rainfall is performed, ~~and~~ the error of these results is within 0.2 mm/h.

Formatted: Line spacing: Double

~~1~~2.4 Data collections

Formatted: Font: (Default) Times New Roman

Formatted: Heading 2, Line spacing: single

~~1~~2.4.1 Determination of weed control effect

To calculate the reductions in weed number and biomass, the shoots of all ~~*Chenopodium*~~*C. album*~~weed~~ plants were cut from three 0.25 m^2 (0.5 m×0.5 m) quadrats in each plot (2 m × 2 m) at 15 and 30 days after treatment (DAT) [~~19~~26]. When mesotrione in soil was analyzed, ~~*Chenopodium*~~*C. album* were collected carefully by taking out from the soil in the open field.~~,~~ ~~*Chenopodium* is the main research object of our experiment, so we didn't collect other weed species.~~ The plant was weighed after washing, the biomass of each weed sample were measured. The efficacy and reduction was calculated by this equation:

Formatted: Line spacing: Double

$$Efficacy\ (\%) = [(C - B)/C] \times 100 \quad (1)$$

where C is the aboveground height of the nontreated control plot and B is the height of an treated plot.

$$Reduction\ (\%) = [(SCP - TP)/SCP] \times 100 \quad (2)$$

Where TP is the weed plant number or biomass in the treated plots and SCP is the sum of the plant number or biomass in the weedy controls.

~~1~~2.4.2 Determination of photosynthetic pigment content

Formatted: Font: (Default) Times New Roman

Formatted: Heading 3, Line spacing: single

Photosynthetic data was collected after 3, 6 and 9 ~~-~~d, weight 0.05 g of mature and intact leaves with the same growth in the middle and upper part of the plant, cut them into pieces, and put them into a stoppered test tube containing 5 ml of 96% ethanol, and soak in a dark place at room temperature for 48 hours until the leaves are milky white and oscillate for several hours during the extraction process. The absorbance of the extract is measured at wavelengths of 470 nm, 649 nm and 665 nm, and the content of chlorophyll a, chlorophyll b, and carotenoid are calculated [~~20~~27]. The calculation formulas are in the following:

$$\mathrm{Ca} = 13.95A_{665} - 6.88\,A_{649} \qquad (3)$$

Formatted: Font: (Default) Times New Roman

Formatted: Indent: First line: 0 ch, Line spacing: Double

$$\mathrm{Cb} = 24.96\,A_{649} - 7.32A_{665} \qquad (4)$$

Formatted: Font: (Default) Times New Roman

$$\mathrm{Cx.c} = (1000\,A_{470} - 2.05\,\mathrm{Ca} - 114.8\,\mathrm{Cb})/245 \qquad (5)$$

Formatted: Font:

~~1~~2.4.3 Determination of chlorophyll fluorescence parameters

Formatted: Font: (Default) Times New Roman

Formatted: Heading 3, Line spacing: single

The chlorophyll fluorescence parameters were tested at 12 h, 24 h, 2 d and 4 d after application respectively. The middle and upper parts of the plant were selected to be fully stretched and mature leaves. The MINI-PAM-Ⅱ portable pulse modulated chlorophyll fluorometer (Walz, Germany), first use the leaf clip of the fluorometer to clamp the ~~*Chenopodium*~~*C. album* for 30 min. Actual photochemical efficiency Y(II), maximum photochemical quantum yield Fv/Fm of leaves, regulated energy dissipation quantum yield Y(NPQ) and non-regulated energy dissipation quantum yield Y(NO) were examined ~~.~~[2~~1~~8].

Formatted: Line spacing: Double

~~1~~2.5 Statistical analyses

Formatted: Font: (Default) Times New Roman

Formatted: Heading 2, Line spacing: single

DPS 6.5 Analysis System was used to conduct all statistical analyses. Data were presented as mean ± standard error of the mean. Duncan’s test was used to analyze significant differences among treatments in growth parameters, chlorophyll content and fluorescence parameters.

Formatted: Line spacing: Double

~~2~~ 3 Results ~~and Discussions~~

3.1 The visual control effect

The reaction symptoms of the *C. album* to the mesotrione become more serious with the time going of application. With the extension of rainfall interval, the growth of *C. album* was severely inhibited, the tip of leaves was more serious, and the heart leave was even completely withered and shed. The survey of weed injury level (Table 3) showed that rainfall reduced the herbicide injury of weed by 1~2 levels at 15 DAT. The weed damage decreased to grade 5 when the rain intensity was 10 mm/h and the interval was 0.5 h at 30 DAT, but other intervals is grade 6.

Table 3. Weed injury level at 15, 30 d after application of mesotrione and simulated rainfall

Rainfall intensity	Interval time	Level of weed injury	
		15 d	30 d
2 $mm \cdot h^{-1}$	CK	6	6
	T1	5	6
	T2	5	6
	T3	6	6
	T4	6	6
6 $mm \cdot h^{-1}$	CK	6	6
	T1	4	6
	T2	5	6
	T3	5	6
	T4	5	6
10 $mm \cdot h^{-1}$	CK	6	6
	T1	4	5
	T2	4	6
	T3	5	6
	T4	6	6

Formatted: Font: Not Bold

Formatted: Heading 1, Line spacing: single

Formatted: Justified, Indent: First line: 2 ch, Line spacing: Double

Formatted: Font color: Auto

Formatted: Font: Italic, Font color: Auto

Formatted: Indent: First line: 0 ch

~~2~~3.2~~1~~ The ~~weed~~ effect of simulated rainfall on mesotrione

After spraying mesotrione, ~~*Chenopodium*~~*C. album* exhibits disease ~~symptoms such as~~like chlorosis and albino of stems and leaves. With applicating the herbicide, the rainfall causes the plant height inhibition rate to decrease, and enhanced gradually increases with the extension of the interval. It is represented in (Table ~~2~~4) that when the rain intensity is 2 mm/h, at 15 DAT, compared with CK, T1,T2 and T3 cause a significant reductions in the inhibition rate of plant height(11.80%、10.87%、9.70%~~11.70%~~, respectively). At 30 DAT, there were no significant difference between treatments and CK. When the rain intensity was 6 mm/h, after 30 days, the inhibition rate of the treatment with only T1 interval significantly decreased by 5.57% compared with CK, while the other treatments had no significant difference compared with CK. In addition to, with the increase of rainfall intensity, there was significantly reduced by 7.53% and 4.91% at T1 and T2 in the inhibition rate of plant height in 10 mm/h, the reduced efficiency were 8.93% and 5.83%, respectively.

The control effect and fresh weight control effect of the plants are belong to the weed control effects, which is increased significantly at 30 DAT compared with 15 DAT. At 15 DAT, the weed control effects of only T1 and T2 were significantly lower than that of CK by 15.06%, 11.83%, 13.31%, and 11.38% in 2 mm/h, the reduced efficiency was more than 13 percent; the rain intensity was at 6 mm/h and 10 mm/h, the weed control effects were significantly lower than CK. At 30 DAT, when the rain intensity was 2 mm/h, the weed control effects were still more than 90% at all treatments. However, the fresh weight control effect of T1 and T2 treatments is significantly lower than CK by 16.34% and 6.90% in 6 mm/h, the reduced efficiency were 16.90% and 7.14%, respectively; compared with CK, T1, T2 and T3 were reduced in the fresh weight control (19.30%, 8.43%, 6.80%, respectively) when the rain intensity is 10 mm/h, and the reduced efficiency was above 7%.

Formatted: Font: (Default) Times New Roman

Formatted: Font: (Default) Times New Roman

Formatted: Heading 2, Line spacing: single

Formatted: Line spacing: Double

Formatted: Default Paragraph Font, Font: (Default) +Body (Calibri), 10.5 pt

Formatted: Default Paragraph Font, Font: (Default) +Body (Calibri), 10.5 pt

Formatted: Default Paragraph Font, Font: (Default) +Body (Calibri), 10.5 pt

Formatted: Default Paragraph Font, Font: (Default) +Body (Calibri), 10.5 pt

Formatted: Default Paragraph Font, Font: (Default) +Body (Calibri), 10.5 pt

Formatted: Default Paragraph Font, Font: (Default) +Body (Calibri), 10.5 pt

Formatted: Default Paragraph Font, Font: (Default) +Body (Calibri), 10.5 pt

Formatted: Default Paragraph Font, Font: (Default) +Body (Calibri), 10.5 pt

Formatted: Default Paragraph Font, Font: (Default) +Body (Calibri), 10.5 pt

Formatted: Default Paragraph Font, Font: (Default) +Body (Calibri), 10.5 pt

Formatted: Default Paragraph Font, Font: (Default) +Body (Calibri), 10.5 pt

Formatted: Default Paragraph Font, Font: (Default) +Body (Calibri), 10.5 pt

Formatted: Default Paragraph Font, Font: (Default) +Body (Calibri), 10.5 pt

Formatted: Default Paragraph Font, Font: (Default) +Body (Calibri), 10.5 pt

Table 4. Effect of simulated rainfall on the weed control of mesotrione

Rainfall intensity	Interval time	Plant height inhibition rate/%				Plant control efficacy/%				Fresh weight control efficacy/%			
		15 d	Reduced efficiency	30 d	Reduced efficiency	15 d	Reduced efficiency	30 d	Reduced efficiency	15 d	Reduced efficiency	30 d	Reduced efficiency
2 mm·h^{-1}	CK	65.29±1.81a	——	84.29±1.14a	——	83.09±1.78a	——	96.42±3.39a	——	81.91±2.71a	——	96.67±3.01a	——
	0.5 h	53.49±2.10b	18.07	80.79±2.11a	4.15	68.03±4.22b	18.12	91.20±5.12a	5.41	68.60±2.74b	16.25	93.89±2.83a	2.88
	1 h	54.42±1.00b	16.65	81.85±3.06a	2.89	71.26±3.73b	14.24	93.68±2.80a	2.54	70.53±5.82b	13.89	94.39±2.85a	2.36
	2 h	55.59±1.95b	14.86	82.37±2.24a	2.28	77.92±5.58ab	6.22	95.32±1.37a	1.14	76.45±3.67ab	6.67	95.28±3.54a	1.44
	4 h	62.12±2.68a	4.86	83.94±1.89a	0.42	81.84±3.38ab	1.50	96.08±7.82a	0.35	80.26±4.78a	2.01	95.74±2.53a	0.96
6 mm·h^{-1}	CK	65.29±1.81a	——	84.29±1.14a	——	83.09±1.78a	——	96.42±3.39a	——	81.91±2.71a	——	96.67±3.01a	——
	0.5 h	41.19±1.42c	36.91	78.72±2.67b	6.61	43.00±4.39c	48.25	76.98±4.01c	20.16	51.16±4.82c	37.54	80.33±3.20c	16.90
	1 h	43.27±2.06c	33.73	81.02±1.84ab	3.88	56.69±2.46b	31.77	85.21±7.30b	11.60	54.92±4.39c	32.95	89.77±6.00b	7.14
	2 h	50.71±1.72b	22.33	82.14±2.46a	2.55	63.30±3.94b	23.82	89.50±5.51b	7.18	64.42±3.43b	21.35	94.28±3.68a	2.47
	4 h	54.83±1.16b	16.02	83.44±1.81a	1.01	65.73±2.37b	20.89	90.41±4.72b	6.23	68.74±4.01b	16.07	96.28±2.15a	0.40
10 mm·h^{-1}	CK	65.29±1.81a	——	84.29±1.14a	——	83.09±1.78a	——	96.42±3.39a	——	81.91±2.71a	——	96.67±3.01a	——
	0.5 h	40.32±2.38c	38.24	76.76±1.98c	8.93	39.20±3.63c	52.82	68.96±3.63d	28.48	43.09±2.45c	47.39	77.37±2.12c	19.96
	1 h	42.86±1.61bc	34.35	79.38±2.93bc	5.83	42.96±1.92c	48.30	78.66±2.51c	18.42	55.12±5.23b	32.70	88.20±4.31b	8.76
	2 h	47.16±1.05bc	27.77	82.12±1.30ab	2.57	55.90±2.61bc	32.72	82.17±1.53c	14.78	57.16±4.23b	30.22	89.87±3.39b	7.03
	4 h	53.94±0.99b	17.38	82.36±1.28ab	2.29	65.46±1.17b	21.22	87.68±3.43b	9.06	62.08±3.62b	24.21	94.09±4.09a	2.67

Data are shown as mean ±SE with the different lowercase letters in the same column represent significant differences ($P<0.05$).

Formatted: Font: Bold

Formatted: Font: Bold

Formatted: Indent: First line: 0 ch

~~2~~3.~~2~~ 3 Effects of simulated rainfall on photosynthetic pigment content of ~~*Chenopodium*~~*C. album* leaves after spraying mesotrione

~~2~~3.~~2~~3.1 The effect of simulated rainfall on the carotenoid content of ~~*Chenopodium*~~*C. album* after spraying mesotrione

The data in Figure 1 showed that the corresponding rainfall intensity was 2 mm/h~~.~~, ~~Compared~~ compared with CK at 3 DAT, T1 and T2 treatments show a significant increase, ~~the increase have been~~ 24.73% and 17.2%. Compared with all interval treatments, CK at 6 DAT did not see a big difference, which could indicate that the rainfall in this period is not capable of preventing the albino process of ~~*Chenopodium*~~*C. album*.

When the rainfall intensity reached 6 mm/h, it could find that the corresponding carotenoid content of T1 and T2 treatments increased at 3 and 6 DAT, and the ranges were 21.85%, 13.89%, 20.00% and 21.91%, respectively. Except that the T4 interval treatment was a bit different, the carotenoid content involved in other treatments looked higher than the content contained in CK. And the growth rates are 39.78%, 36.56%, 29.03% and 33.32%, 19.32%, 15.79%.

Figure1: Effect of simulated rainfall on carotenoid content in leaves of *C. album* treated by mesotrione after 3(A) and 6(B) DAT. X-axis represents different rainfall intensity; y-axis represents the carotenoid content of leaves. CK means that there was no rain after the application of mesotrione, T1, T2, T3, and T4 represent different rainfall interval times (0.5 h, 1 h, 2 h, and 4 h). Effect of simulated rainfall on carotenoid content in leaves of *C. album* treated by mesotrione after 3 DAT (A) and 6 DAT (B). Lowercase letters in the same column indicate significant differences at the 0.05 level.

Formatted: Font: 9 pt, Underline color: Custom Color(RGB(250,80,80)), Font color: Text 1

Formatted: Indent: First line: 0 ch

Formatted: Font: (Default) Times New Roman

Formatted: Heading 2, Line spacing: single

Formatted: Font: (Default) Times New Roman

Formatted: Font: (Default) Times New Roman

Formatted: Font: (Default) Times New Roman

Formatted: Font: (Default) Times New Roman

Formatted: Line spacing: Double

Formatted: Indent: First line: 0 ch, Line spacing: Double

~~2~~3.~~2~~3.2 The effect of simulated rainfall on the chlorophyll content of ~~*Chenopodium*~~*C. album* after spraying mesotrione

Like the carotenoid content, the content of chlorophyll a and chlorophyll b decreased gradually and showed a decreasing trend with the extension of rainfall interval. As shown in Table ~~2~~5, when the rain intensity was 2 mm/h, the chlorophyll a and chlorophyll b contents of all interval treatments were not significantly different than CK at 6 DAT. At the same time, chlorophyll a+b content under T1 treatment has an increase of 27.86% compared with CK, the difference was not very significant between the other treatments and CK.

Under the situation of 3 DAT, the rainfall intensity reached 6 mm/h. In this case, in addition to the different results produced by T4 processing, the chlorophyll a content corresponding to other treatments has a more significant increment compared to CK~~,~~.~~;~~ In addition, when the T1 and T2 intervals were used for treatment, the chlorophyll b content would increase significantly, with an increase of 46.41% and 35.38%. On the other hand, ~~when DAT was 6 DAT,~~ all treatments at certain intervals show that the contents of chlorophyll a and chlorophyll b were much higher than that situation of CK at 6 DAT. It could be claimed that compared with carotenoids, 6 mm/h rainfall had a greater impact on chlorophyll a and b. Although the interval is only 4 hours, it has a higher content than CK. When the rainfall intensity under 3 DAT reached 10 mm/h, the chlorophyll a content corresponding to all treatment intervals was at a high level, exceeding the chlorophyll content of CK. In the meantime, there was no significant difference between T1, T2 and T3 treatment.~~. intervals.~~

Formatted: Font: (Default) Times New Roman

Formatted: Heading 3, Line spacing: single

Formatted: Font: (Default) Times New Roman

Formatted: Font: (Default) Times New Roman

Formatted: Line spacing: Double

Table 5. Effect of simulated rainfall on photosynthetic pigment content in leaves of *C. album* treated by mesotrione

Rainfall intensity	Interval time	Chlorophyll a content(mg·g^{-1} FW)		Chlorophyll b content (mg·g^{-1} FW)		Chlorophyll a+b content(mg•g^{-1} FW)	
		3 d	6 d	3 d	6 d	3 d	6 d
2 mm·h^{-1}	CK	0.92±0.058b	0.79±0.101a	0.27±0.020c	0.26±0.027a	1.19±0.040c	1.05±0.075b
	T1	1.39±0.021a	1.00±0.078a	0.46±0.012a	0.35±0.062a	1.85±0.012a	1.35±0.019a
	T2	1.06±0.098b	0.90±0.049a	0.38±0.012b	0.31±0.030a	1.44±0.087b	1.21±0.020ab
	T3	1.09±0.074b	0.82±0.074a	0.34±0.023b	0.32±0.027a	1.43±0.054b	1.14±0.048b
	T4	0.95±0.040b	0.79±0.045a	0.35±0.0208b	0.28±0.025a	1.30±0.030bc	1.07±0.069b
6 mm·h^{-1}	CK	0.92±0.058b	0.79±0.101b	0.27±0.020c	0.26±0.027b	1.19±0.040c	1.05±0.075b
	T1	1.29±0.075a	1.18±0.078a	0.51±0.021a	0.47±0.009a	1.80±0.010a	1.65±0.086a
	T2	1.24±0.031a	1.14±0.067a	0.37±0.035b	0.46±0.012a	1.57±0.030b	1.60±0.055a
	T3	1.20±0.038a	1.14±0.127a	0.35±0.020bc	0.42±0.018a	1.59±0.06b	1.56±0.111a
	T4	0.98±0.050b	0.97±0.051ab	0.35±0.037bc	0.31±0.055b	1.34±0.010c	1.28±0.015b
10 mm·h^{-1}	CK	0.92±0.058c	0.79±0.101b	0.27±0.020c	0.26±0.027c	1.19±0.040c	1.05±0.075b
	T1	1.33±0.064a	1.21±0.079a	0.53±0.036a	0.51±0.031a	1.86±0.099a	1.72±0.071a
	T2	1.34±0.064ab	1.19±0.041a	0.40±0.003b	0.49±0.021a	1.75±0.067a	1.68±0.056a
	T3	1.14±0.020ab	1.16±0.113a	0.38±0.022b	0.44±0.026b	1.51±0.041b	1.57±0.139a
	T4	1.18±0.083b	1.15±0.050a	0.36±0.035b	0.37±0.019b	1.54±0.050b	1.53±0.037a

Data are shown as mean ±SE with the different lowercase letters in the same column represent significant differences ($P<0.05$).

Formatted: Font: Italic

Formatted: First line: 0 ch, Line spacing: Double

Formatted: Font: Not Bold

~~2~~3.~~3~~ 4 Effects of simulated rainfall on chlorophyll fluorescence parameters of ~~*Chenopodium*~~*C. album* after spraying mesotrione

~~2~~3.~~3~~4.1 The effect of 2 mm/h rain intensities on chlorophyll fluorescence parameters after spraying mesotrione

These two types of indicators Fv/Fm and Y(II) in the leaves showed a certain decline after using mesotrione. At the same time, all processing intervals were showing an upward trend. However, if the time between rains continued to extend, there was a possibility that there might be an overall decline between the two (Figure 2). The max value is T1 after the mesotrione, and the minimum will be T4. After taking relevant treatments, there is no significant difference in Fv/Fm indicators of other treatments compared with CK. But this does not include T1 treatment, T1 treatment caused a significant increase in Fv/Fm, with an amplitude of 27.65%.~~;~~

In addition, it can be seen that there is no big difference between T4 interval treatment Y(II) and CK, and the remaining treatment Y(II) has a greatly increasment , whose amplitude are 43.85%, 36.97%~~,~~ and 35.60%, respectively. On the 4th day, if T1 treatment is not considered, the Fv/Fm of other treatments all show a certain increase. Compared with CK, the corresponding incidence rates are 66.54%, 46.62% and 23.42%, respectively; In the figure, the Y(II) treatment at T1 and T2 interval both increased to a certain extent, which increased by 210.88% and 127.98% respectively (Figure 2.A-B). In addition, after the use of mesotrione, the entire graph trend showed that Y (NPQ) would decrease with the extension of the rain interval, but the value of Y (NO) would continue to increase. If T1 was not considered at 4 DAT, the Y (NPQ) of T2 interval treatment would increase significantly, and there might not be much difference between other treatments and CK Y (NPQ); in addition, the Y (NO) index after T1 interval treatment had a certain drop of 10.38%. In contrast, there were not many differences between other treatments and CK (Figure 2.C-D).

Figure2: Effect of 2 mm/h rain intensity on chlorophyll fluorescence parameters of *C. album*

Formatted: Font: (Default) Times New Roman
Formatted: Heading 2, Line spacing: single
Formatted: Font: (Default) Times New Roman
Formatted: Font: (Default) Times New Roman
Formatted: Font: (Default) Times New Roman
Formatted: Line spacing: Double
Formatted: Font: 10.5 pt
Formatted: Indent: First line: 2 ch, Line spacing: Double
Formatted: Font: 10.5 pt
Formatted: Font: 10.5 pt
Formatted: Superscript
Formatted: Indent: First line: 0 ch, Line spacing: Double

treated by mesotrione. CK and T1, T2, T3, T4 on the x-axis represent no rain and different rainfall interval times (0.5 h, 1 h, 2 h, and 4 h) respectively; y-axis represents chlorophyll fluorescence parameters. Diamonds, squares, triangles and circles represent different times after treatment (12 h, 24 h, 2 d and 4 d, respectively). A)the maximum photochemical quantum efficiency (Fv/Fm); B)the actual photochemical quantum yield Y(II); C)the regulated energy dissipation quantum yield Y(NPQ); D)the non-regulated energy dissipation quantum yield Y(NO) of *C. album* treated by mesotrione. Lowercase letters in the same column indicate significant differences at the 0.05 level.

~~2~~3.~~3~~4.2 Effect of 6 mm/h rain intensities on chlorophyll fluorescence parameters after spraying mesotrione

Based on the situation shown in Figure 2, if the rain interval continued to extend, the indicators Fv/Fm and Y(II) would both show a downward trend. In particular, if the application exceeds 12 hours, the effect of mesotrione will be greatly reduced due to rainfall. In addition, the Fv/Fm index values are much higher than in the CK case in all treatment intervals, and the corresponding growth rates are 42.47%, 33.27%, 24.97% and 17.96%, respectively. In addition, on the 4th day, the condition of the patients treated with the T4 interval will be relieved, and the Fv/Fm index will no longer be significantly different from that in the case of CK. It should also be noted that compared with the case of CK, the Fv/Fm of other treatments will be greatly improved, and the corresponding increases are 82.59%, 72.07% and 62.67% respectively (Figure 3.A).

Compared to the situation of CK, Y(II) of ~~*Chenopodium*~~*C. album* had a increasment by 528.50% and 398.96% at 4 DAT for T1, and T2 treatments~~,~~. And it is noted that it is almost the same as T3, T4 treatments and CK (Figure 3. B), stating that if the coppresponding rain intensity reached 6 mm/h, the rainfall within 1 hour could be give a great influence at the efficacy of mesotrione.

The Y(NPQ) value of ~~*Chenopodium*~~*C. album* was measured at different periods, and its magnitude was as follows: 24 hours after treatment>12 hours after treatment>2 d after treatment>4

Formatted: Font:

Formatted: Font: (Default) Times New Roman

Formatted: Font: (Default) Times New Roman

Formatted: Heading 3, Line spacing: single

Formatted: Line spacing: Double

Formatted: Superscript

Formatted: Font: Not Bold

Formatted: Default Paragraph Font, Font: (Default) +Body (Calibri), 10.5 pt

Formatted: Indent: First line: 2 ch, Line spacing: Double

Formatted: Default Paragraph Font, Font: (Default) +Body (Calibri), 10.5 pt

Formatted: Default Paragraph Font, Font: (Default) +Body (Calibri), 10.5 pt

Formatted: Line spacing: Double

d after treatment. The photosynthetic inhibitory effect of ~~*Chenopodium*~~*C. album* was the strongest at 4 DAT. Compared with 12 hours after application, because of the self-protection mechanism of ~~*Chenopodium*~~*C. album*, Y(NPQ) will has a slower increasment, and then slowly decreased; Y(NO) showed that with the extension of the interval and the passage of the reaction time gradually increase. Compared with CK at 24 hours after the mesotrione, Y(NPQ) in all interval treatments decreased significantly; Y(NO) in all interval treatments was significantly lower than CK, T1 and T2 treatments would not show much differences.(Figure 3.C-D)).

Figure3: Effect of 6 mm/h rain intensity on chlorophyll fluorescence parameters of *C. album* treated by mesotrione. CK and T1, T2, T3, T4 on the x-axis represent no rain and different rainfall interval times (0.5 h, 1 h, 2 h, and 4 h) respectively; y-axis represents chlorophyll fluorescence parameters. Diamonds, squares, triangles and circles represent different times after treatment (12 h, 24 h, 2 d and 4 d, respectively). A)the maximum photochemical quantum efficiency (Fv/Fm); B)the actual photochemical quantum yield Y(II); C)the regulated energy dissipation quantum yield Y(NPQ); D)the non-regulated energy dissipation quantum yield Y(NO) of *C. album* treated by mesotrione. Lowercase letters in the same column indicate significant differences at the 0.05 level.

~~2~~3.~~3~~4.3 The effect of 10 mm/h rain intensities on chlorophyll fluorescence parameters after spraying mesotrione

In addition, based on the display status of the graph, rainfall will increase the Fv/Fm and Y(II) values of ~~*Chenopodium*~~*C. album* (Figure 3). However, this situation will be weakened by the extension of the rainfall time, and this trend will gradually flatten out. Compared with the results under CK conditions, Fv/Fm show the great increment by 25.14%~43.93% for all interval ~~treatment~~s at 12 h and 4 days after treatment~~ application~~. Ignoring the results from T4 treatment, others would demonstrate the better increment by 85.08% and 78.31%, 71.54%, which was much higher than CK, and T1, T2 and T3 treatment, Y(II) give a good growth from the

Formatted: Font: (Default) Times New Roman, 12 pt, Bold
Formatted: Font: (Default) Times New Roman, 12 pt
Formatted: Font: 10.5 pt
Formatted: Indent: First line: 0 ch, Line spacing: Double
Formatted: Font: (Default) Times New Roman
Formatted: Heading 3, Line spacing: single
Formatted: Font: (Default) Times New Roman
Formatted: Line spacing: Double
Formatted: Default Paragraph Font, Font: (Default) +Body (Calibri), 10.5 pt
Formatted: Default Paragraph Font, Font: (Default) +Body (Calibri), 10.5 pt
Formatted: Default Paragraph Font, Font: (Default) +Body (Calibri), 10.5 pt

observation, which means that the possible influence of mesotrione on *Chenopodium**C. album* become much weaker when it is raining. If the rain intensity arrives at 10 mm/h, T1, T2 and T3 treatment had a much stronger restrictive effect mesotrione (Figure 4.A-B). Y(NPQ) will fluctuate when it suffers from rain intensity of 2 mm/h and 6 mm/h. It might have a growth at 24 h after treatment compared with 12 h, and then slowly declined and minimized after 4 d, except for the T4 treatment. T1, T2 and T3 treatments is possible to have an impact on the mesotrione of *Chenopodium**C. album*. Compared with CK, Y(NPQ) gives a higher increment at 422.82%, 220.58%, and 199.11% (Figure 4.C).

Figure4: Effect of 10 mm/h rain intensity on chlorophyll fluorescence parameters of *C. album* treated by mesotrione. CK and T1, T2, T3, T4 on the x-axis represent no rain and different rainfall interval times (0.5 h, 1 h, 2 h, and 4 h) respectively; y-axis represents chlorophyll fluorescence parameters. Diamonds, squares, triangles and circles represent different times after treatment (12 h, 24 h, 2 d and 4 d, respectively). A)the maximum photochemical quantum efficiency (Fv/Fm); B)the actual photochemical quantum yield Y(II); C)the regulated energy dissipation quantum yield Y(NPQ); D)the non-regulated energy dissipation quantum yield Y(NO) of *C. album* treated by mesotrione. Lowercase letters in the same column indicate significant differences at the 0.05 level.

3 4 Discussion

With the continuous acceleration of the process of agricultural modernization and the gradual transfer of rural labor to cities, chemical control has become the dominant method in the field of weed control due to its high efficiency and practicality [2229-2330]. Temperature, humidity, light, wind, and precipitation will affect the effectiveness of the mesotrione [31-32]. Global warming causes the rainfall increases and the rainfall mainly is the short-duration heavy rainfall in summer, heavy monsoon rains would increase the risk of crop failure within regions or globally [33-34]. The slow-moving cold front delays the occurrence of peak rainfall but enhances the rainfall intensity, the

Formatted: Default Paragraph Font, Font: (Default) +Body (Calibri), 10.5 pt

Formatted: Default Paragraph Font, Font: (Default) +Body (Calibri), 10.5 pt

Formatted: Default Paragraph Font, Font: (Default) +Body (Calibri), 10.5 pt

Formatted: Default Paragraph Font, Font: (Default) +Body (Calibri), 10.5 pt

Formatted: Indent: First line: 0 ch, Line spacing: Double

Formatted: Font:

Formatted: Font: Not Bold

Formatted: Heading 1, Line spacing: single

Formatted: Line spacing: Double

maximum hourly summer rainfall intensity has increased by about 11.2% on average [35]. ~~However, as a means of intervention in production, herbicides put forward many requirements on natural conditions in practical applications. Temperature, humidity, light, wind, and precipitation will affect the effectiveness of the mesotrione [24-25]. Mesotrione is commonly used as a stem and leaf treatment agent to control broadleaf weeds in corn fields.~~ The scouring effect of rain water reduces the effective components of the medicament attached to the leaf surface and reduces the herbicide effect [~~26~~36].

Zhang *et al*.[~~27~~37] reported that with the increase of the dose of mesotrione, the inhibitory effect on corn growth increased, mainly manifested as a significant decrease in plant height; Zhang *et al*. [~~15~~22] and Wang *et al*. [~~16~~23] reported spraying nitrate. After sulcotrione, the heart leaves of Amaranthus retroflexus were severely chlorosis and then the whole plant will died. The control efficiency of plant and fresh weight were high than common plants; Gao *et al*. [~~28~~38] reported that the effect of mesotrione in the control of weeds increased with weed physiological metabolism intensified, the symptoms are more significant later. Spillman [39] think the rainfall would dilute or wash the medicament on weed's surface, and then the reduced efficacy couldn't have a thorough effect on the weed. In this study, on condition that the rain intensity was 2 mm/h and the rainfall interval was T1, the plant height inhibition rate of ~~*Chenopodium*~~*C. album* was still close to that of no rainfall after application, indicating that a light rain after application would not affect the efficacy of mesotrione. When the rain intensity is 6 mm/h~~. U~~, under the treatment of 2 h interval, with the extension of the rain interval time, the control effect of mesotrione on ~~*Chenopodium*~~*C. album* gradually increases, and the control effect of plant and fresh weight is still above 90%, it only decreased by 0.4%-2.47% compared with CK~~.~~ , ~~The~~ the efficacy of mesotrione was not significantly affected.

The mechanism of action of herbicides is mainly to cause weed death by interfering or inhibiting the physiological metabolism of plants, including photosynthesis, fatty acid synthesis,

pigment synthesis, amino acid metabolism [2940]. HPPD is the only target enzyme for mesotrione plants, and carotenoids are synthesized products of the target enzyme (HPPD) after the catalytic reaction. Therefore, the changes in the mass fraction of photosynthetic pigments in the leaves can reflect the HPPD enzyme in *ChenopodiumC. album*. The level of activity is used as an important indicator of the weed control efficiencyeffectiveness of mesotrione on weed control [3041]. Rainfall not only straightly affected the efficacy of herbicide by changing the absorbing and conduction capabality inside the plants, but also by changing the growth and physiological property indirectly. Zhang *et al*. [3142] found that the carotenoid, chlorophyll a and chlorophyll b content in the leaves of *ChenopodiumC. album* gradually decreased with the time of the herbicide reaction after spraying mesotrione. Zhang *et al.* [3243] thinks that the erosion resistance is better with the increase of retention on leaves. In this experiment, the control effect decreased after spraying mesotrione, may be due to rainfall washing the pesticide retention on the plant surface, which increased the photosynthetic pigment content of *ChenopodiumC. album* leaves, but as the reaction time increased, the content of photosynthetic pigments decreased step by step. However, when the rainfall intensity increased, the content of carotenoids in leaves of *ChenopodiumC. album* was significantly higher than that of the control when the rainfall intensity was 6 mm/h and the interval was within 1 h. If the rainfall intensity continued to add to 10 mm/h, the carotenoid content of leaves of *ChenopodiumC. album* was still significantly increased after 2 h interval times., significant difference disappeared under the 4 h rainfall disposal. It is consistent with the conclusion which was the rainfall after 4 h didn't have effect on the efficacy of the new-style herbiside —Y11049 [44].

Chlorophyll fluorescence is a means of rapidly identifying injury to leaves in the absence of visible symptoms. This is a detailed analysis of causes of change in photosynthetic capacity. It has been widely used in laboratory studies in understanding the mechanism of photosynthesis and the mechanisms by which a range of external factors alter photosynthetic capacity. The measurement of chlorophyll fluorescence also has considerable potential for use in the field situation [3345]. Fv/Fm

represents the strength of photoinhibition after the plant is stressed, reflecting the maximum light energy conversion efficiency in the photosynthetic reaction center, and Y(II) refers to the actual light energy conversion efficiency of the plant photosynthesis process [~~34~~46]. Y(NPQ) is one of the self-protection indicators for plants to convert excess light energy into heat and the degree of light damage to plants is represented by Y(NO) [~~35~~47-48~~36~~]. Yang *et al*. [~~37~~49] found that 2,4-D treatment significantly reduced the Fv/Fm and Y(II) of Jinfen No.107 and Jingu No.29 millet seedling leaves. Guo *et al*. [~~38~~50] reported that after spraying chipton, the Fv/Fm of millet has an increasing inhibitory effect as the increased of the spraying concentration. We found that Fv/Fm, Y(II), Y(NPQ) of ~~*Chenopodium*~~*C. album* leaf gradually decreased with the extension of the interval, Y(NO) showed an upward trend conversely. We had tested the Y(NPQ) of the ~~*Chenopodium*~~*C. album* leaf after spraying mesotrione for 24 h, it was higher than that of 12 h, indicating that the ~~*Chenopodium*~~*C. album* can relieve the caused by mesotrione to a certain extent by increasing heat dissipation after being slightly stressed. Photodamage, the photochemical reaction efficiency continues to decline due to the serious herbicide stress, and it shows a downward trend as the rain interval increases. Therefore, the efficacy of mesotrione has weakened, which is mainly due to the increase in leaf photosynthetic pigment content, and the increase of photosynthetic capacity of photosystem II, which affects the control effect of mesotrione and the growth of ~~*Chenopodium*~~*C. album* and photosynthesis. *C. album*'s growth difference under different disposal is directly connected with the absorption and conduction of mesotrione, it needs further discussion about the loss of the efficacy and the residual on the surface of leaves.

When the rainfall intensity was 6 mm/h within 1 h after mesotrione spraying, or the rainfall intensity was 10 mm/h within 2 h, the loss of plant control effect was above 11%. Oils adjuvants could reduce the surface tension and contact angle of the herbicide liquid, then the herbicide would have better wettability, adhesiveness, penetrating quality and resistance to rain washing capability and ultimately the efficacy would be increased [51]. Tao *et.al* [52] researched that adjuvants could

Formatted: Font: 12 pt

Formatted: Indent: First line: 2 ch, Line spacing: Double

Formatted: Font: 12 pt

Formatted: Font: 12 pt, Italic

Formatted: Font: 12 pt

Formatted: Font: 12 pt

increase the prevention and control effect if the rainfall appears in 4 h after mesotrione was applied. Terrible environment would reduce herbicide's efficacy, but effective ingredient in the adjuvants could increase herbicide's prevention and control effect by counteracting the influence. And how to choose adjuvants needs to be further studied ~~further~~.

~~4~~ 5 Conclusion

Formatted: Font: Not Bold

Formatted: Heading 1, Line spacing: single

In conclusion, rainfall reduced the control effect of mesotrione on ~~*Chenopodium*~~*C. album*, one of the reasons was the increase of photosynthetic pigment content and the enhancement of photosystem II activity. The 2 mm/h ~~raininess~~ not significantly effected ~~mesotrione~~ the control effect of mesotrione; In the circumstance of 6 mm/h of the rainfall intensity, the control effect of mesotrione was significantly reduced within 0.5 h and 1 h, the fresh weight control efficacy decreased by 16.90% and 7.14%, respectively; and in the 10 mm/h of the rainfall intensity, the plant control efficacy was reduced by 28.48% and 18.42%, respectively, even the interval of rainfall was 2 h, it still showed a reduction rate of 14.78 percent.

Formatted: Line spacing: Double

~~the effect of mesotrione was significantly reduced by rainfall within 2 h.~~

Formatted: Indent: First line: 0 ch, Line spacing: Double

Acknowledgments

We would express sincere appreciation to two anonymous reviewers for their thoughtful and valuable comments, which helped us to improve this manuscript. Thanks are also due to Professor X.Y. Yuan of Shanxi Agricultural University for suggestions on the manuscript. ~~This paper was supported by the system of National Modern Agriculture Technology (CARS-06-13.5-A28), the program for the Top Young Innovative Talents of Shanxi Agricultural University (TYIT201406) and the Key research and development plan of Shanxi Province (201903D22103)..~~

Formatted: Line spacing: Double

Authors' contribution

The author would like to thank all authors for their help in the experience and manuscript. Mengmeng Sun was responsible for preparing the final version of manuscript. Xiangyang Yuan

Formatted: Line spacing: Double

helped conceive the experiments and was the project director. Meijun Guo and Shuqi Dong were involved in review the language of the manuscript. Xie Song and helped analysis and interpretation of data. Shuai Guo and Yanfen Li were involved in literature collection. Xiaoxin Shi was involved the meteorological datas collection and calculation.

Formatted: Line spacing: Double

Figure1: Effect of simulated rainfall on carotenoid content in leaves of *Chenopodium* treated by mesotrione after 3(A) and 6(B) DAT. X axis represents different rainfall intensity; y axis represents the carotenoid content of leaves. CK means that there was no rain after the application of mesotrione, T1, T2, T3, and T4 represent different rainfall interval times (0.5 h, 1 h, 2 h, and 4 h). Effect of simulated rainfall on carotenoid content in leaves of Chenopodium treated by mesotrione after 3 DAT (A) and 6 DAT (B). Lowercase letters in the same column indicate significant differences at the 0.05 level.

Figure2: Effect of 2 mm/h rain intensity on chlorophyll fluorescence parameters of *Chenopodium* treated by mesotrione. CK and T1, T2, T3, T4 on the x-axis represent no rain and different rainfall interval times (0.5 h, 1 h, 2 h, and 4 h) respectively; y-axis represents chlorophyll fluorescence parameters. Diamonds, squares, triangles and circles represent different times after treatment (12 h, 24 h, 2 d and 4 d, respectively). A)the maximum photochemical quantum efficiency (Fv/Fm); B)the actual photochemical quantum yield Y(II); C)the regulated energy dissipation quantum yield Y(NPQ); D)the non-regulated energy dissipation quantum yield Y(NO) of Chenopodium treated by mesotrione. Lowercase letters in the same column indicate significant differences at the 0.05 level.

Figure3: Effect of 6 mm/h rain intensity on chlorophyll fluorescence parameters of *Chenopodium* treated by mesotrione. CK and T1, T2, T3, T4 on the x-axis represent no rain and different rainfall interval times (0.5 h, 1 h, 2 h, and 4 h) respectively; y-axis represents chlorophyll fluorescence parameters. Diamonds, squares, triangles and circles represent different times after

~~treatment (12 h, 24 h, 2 d and 4 d, respectively). A)the maximum photochemical quantum efficiency (Fv/Fm); B)the actual photochemical quantum yield Y(II); C)the regulated energy dissipation quantum yield Y(NPQ); D)the non-regulated energy dissipation quantum yield Y(NO) of Chenopodium treated by mesotrione. Lowercase letters in the same column indicate significant differences at the 0.05 level.~~

~~**Figure4: Effect of 10 mm/h rain intensity on chlorophyll fluorescence parameters of *Chenopodium* treated by mesotrione.** CK and T1, T2, T3, T4 on the x-axis represent no rain and different rainfall interval times (0.5 h, 1 h, 2 h, and 4 h) respectively; y-axis represents chlorophyll fluorescence parameters. Diamonds, squares, triangles and circles represent different times after treatment (12 h, 24 h, 2 d and 4 d, respectively). A)the maximum photochemical quantum efficiency (Fv/Fm); B)the actual photochemical quantum yield Y(II); C)the regulated energy dissipation quantum yield Y(NPQ); D)the non-regulated energy dissipation quantum yield Y(NO) of Chenopodium treated by mesotrione. Lowercase letters in the same column indicate significant differences at the 0.05 level.~~

References

[1] ~~DAS~~ Das T-K, YadurajuADURAJU N-T. The effects of combining modified sowing methods with herbicide mixtures on weed interference in wheat crops. International Journal of Pest Management, 2012;, 58(4): 310-319. doi: 10.1080/09670874.2012.680933

[2] ~~LI~~ Li X-J. Main problems and management strategies of weeds in agricultural fields in China in recent years. Plant Protection, 2018;, 44(5): 77-84. doi: 0.16688/j.zwbh.2018322 (in Chinese)

[3] Poonia, A, Upadhayay, A. *Chenopodium album* Linn: review of nutritive value and biological properties. Journal of Food Science and Technology volume, 2015; 52(7): 3977–3985. doi: 10.1007/s13197-014-1553-x

Formatted: Indent: First line: 0 ch, Line spacing: Double

Formatted: Font:

Formatted: Font: Not Bold

Formatted: Heading 1, Line spacing: single

Formatted: Line spacing: Double

Formatted: Font: (Default) Times New Roman

Formatted: Font: Italic

[4] Gao YY. Research on *Chenopodium* in ancient southwest China. IOP Conference Series Earth and Environmental Science, 2021; 621: 012124. doi: 10.1088/1755-1315/621/1/012124

[5] Manila B, Surendra S, Saurabh P, Bhumesh K. A pharmacological comprehensive review on *Chenopodium album* L. International Journal of Current Research, 2020; 12(12): 15360-15368. doi: 10.24941/ijcr.40425.12.2020

[36] Vartika SSINGH V, Sweta GGUPTA S, Hema SSINGH H, Akhilesh SRRAGHUBANSHI AS. Ecophysiological characteristics of five weeds and a wheat crop in the Indo-Gangetic Plains, India. Weed Biology and Management, 2015; ,15(3):102-112. doi: 10.1111/wbm.12073

[7] Bajwa AA, Zulfiqar U, Sadia S, Bhowmik P, Chauhan BS. A global perspective on the biology, impact and management of *Chenopodium album* and *Chenopodium murale*: two troublesome agricultural and environmental weeds. Environmental Science and Pollution Research, 2019; 26: 5357–5371. doi: 10.1007/s11356-018-04104-y

[8] Conley SP, Stoltenberg DE, Boerboom CM, Larry KB. Predicting soybean yield loss in giant foxtail (Setaria faberi) and common lambsquarters (*Chenopodium album*) communities. Weed Science, 2003; 51(3): 402-407. doi: 10.1614/0043-1745(2003)051[0402:PSYLIG]2.0.CO;2

[9] Tehmina A, Rukhsana B. Competition Losses Caused by *Rumex dentatus* L. and *Chenopodium album* L. in Wheat (*Triticum aestivum* L.). The Philippine Agricultural Scientist, 2010; 93(3):

[10] Sarabi V, Mahallati MN, Nezami A, Mohassel MHR. Effects of the relative time of emergence and the density of common lambsquarters (*Chenopodium album*) on corn (*Zea mays*) yield. Weed Biology and Management, 2011; 11(3):127-136. doi: 10.1111/j.1445-6664.2011.00414.x

[411] YANG Yang H-H, WANG Wang L-Q, XIE Xie Z-J, YU Yu Q, LIU Liu G, TAN Tan H-R. The tyrosine degradation gene hppD is transcriptionally activated by HpdA and repressed by HpdR in Streptomyces coelicolor, while hpdA is negatively autoregulated and repressed by HpdR. Molecular Microbiology, 2007;, 65(4): 1064-1077. doi: 10.1111/j.1365-2958.2007.05848.x

[512] GUO Guo W-L, FENG Feng L, CUI CuiY, WU Wu D-D, ZHANG Zhang T-J, ZHANG

Formatted: Font: Italic
Formatted: Font: (Default) Times New Roman
Formatted: Font: (Default) Times New Roman, 12 pt
Formatted: Font: Italic
Formatted: Font: Italic
Formatted: Font: Italic
Formatted: Font: (Default) Times New Roman, 12 pt
Formatted: Font: Italic
Formatted: Font: Italic
Formatted: Font: Italic
Formatted: Font: Italic
Formatted: Font: Italic
Formatted: Font: (Default) Times New Roman

Zhang C, ~~TIAN~~ et al~~X S~~. Weed control effect of 30% nicosulfuro•atrazine•mesotrione oil dispersion and the safety to sweet corn in field. Journal of Maize Sciences, 2020;~~,~~ 28(4): 178-183. doi: 10.13597/j.cnki.maize.science.20200426 (in Chinese)

[~~6~~13] ~~CHEN~~ Chen H-Y~~, ~~, ~~DENG~~ Deng G-Q, ~~FU~~ Fu H-Y, ~~SHE~~ She Y-B. Advances in detection of systemic pesticide residues in agricultural products. Chemical Reagents, 2020;~~,~~ 42(10): 1139-1147. doi: 10.13822/j.cnki.hxsj.2020007659 (in Chinese)

[~~7~~14] ~~TAO~~ Tao B. Practical ~~Techniques~~ techniques for ~~Chemical~~ chemical ~~Control~~ control of ~~Weeds~~weeds. 1st ed. Beijing: Chemical Industry Press, 2013~~:~~; 255.

[~~8~~15] Wu J–Y, ~~S.~~Mathiassen S,~~ P~~ Kudsk P. The influence of weed species, leaf stage and rainfastness on the performance of nicosulfuron. Chinese Journal of Pesticide Science, 2003(1): 77-81. (in Chinese)

[~~9~~16] ~~LUO~~ Luo Z-R, ~~FU~~ Fu Q, ~~XIE~~ Xie Z-B. Effect of simulated rainfall on the weeding effect of paraquat. Journal of Fruit Science, 1995(2): 104-106. doi: 10.13925/j.cnki.gsxb.1995.02.011 (in Chinese)

[~~10~~17] ~~WANG~~ Wang C-F, ~~YOU~~ You Y, Wang~~UANG~~ J, ~~WU~~ Wu J-Y, ~~CHEN~~ Chen F, ~~WANG~~ Wang Q—H. Effect of turf-mowing and simulated rainfall on the efficiency of bensulfuron-methyl•metsulfuron-methyl. Journal of Fujian Agriculture and Forestry ~~UniversitY~~University(Natural Science Edition), 2009~~,~~; 38(4): 351-355. (in Chinese)

[~~11~~18] ~~WANG~~ Wang Q-H, ~~WU~~ Wu J-Y, ~~JIAGN~~ Jiang GQ. Effects of rainfall and adjuvants on phytotoxicity of imazethapyr. Chinese Journal of Pesticide Science, 2003(3): 55-58. (in Chinese)

[~~12~~19] ~~GAO~~ Gao S, ~~ZHANG~~ Zhang Z-J, An~~N~~ W-L, Lin~~IN~~ C-F, ~~YANG~~ Yang Y-T, ~~MA~~ Ma H-J, ~~LI~~ ~~M~~et al. Biological activity and safety of herbicide mesotrione in corn field. Agrochemicals, 2004(10): 469-471. doi: 10.16820/j.cnki.1006-0413.2004.10.015 (in Chinese)

[~~13~~20] ~~ZHANG~~ Zhang Y, Lv~~V~~ A-Q, ~~WU~~ Wu C-X, ~~LU~~ Lu X-T, ~~MA~~ Ma C. Controlling weeds with mesotrione 15% SC in bluegrass lawn. Journal of Weed Science, 2013~~,~~; 31(4): 54-56. doi: 0.19588/j.issn.1003-935x.2013.04.018 (in Chinese)

Formatted: Line spacing: Double

[1421] WANG Wang Z, YAO Yao A-Q. Mesotrione bioactivity and selectivity to corn. Journal of Weed Science, 2014, ; 32(2): 59-61. doi: 10.19588/j.issn.1003-935x.2014.02.017 (in Chinese)

[1522] Vranjes F, Vrbnicanin S–, Nedeljkovic D–, Savic A, Bozic Det al. The response of Chenopodium Chenopodium –album L. and Abutilon theophrasti Medik. to reduced doses of mesotrione[J]. Journal of Environmental ence and Health Part B, 2019, ; 54(7):1-7. doi: 10.1080/03601234.2019.1616980

[1623] Dayan FE—, Zaccaro M–D–M. Chlorophyll fluorescence as a marker for herbicide mechanisms of action[J]. Pesticide Biochemistry & and Physiology, 2012, ; 102(3):189-197. doi: 10.1016/j.pestbp.2012.01.005

[1724] HAO Hao Z-C, WANG Wang H, DUNZHU Dunzhu J-C, JU Ju Q. Spatiotemporal variations of soil moisture across Shanxi province during 2003-2012, . Acta Scientiarum Naturalium Universitatis Sunyatseni, 2014, ; 53(5):134-141. doi: 10.13471/j.cnki.acta.snus.2014.05.047 (in Chinese)

[1825] LI Li D-K, ZOU Zou Y-X, MA Ma X-Z, ZHANG Zhang F-Z, DongONG P, LaA Y-P. Field efficacy and rain resistance of fungicides against potato late blight. China Plant Protection, 2012, ; 32(5): 50-52+59. (in Chinese)

[1926] CUI Cui H-L, JiaIA F, QuanUAN Z-H, CuiUI H-L, LiI X-J. Effects of glyphosate on weed control, soybean safety and weed occurrence in transgenic herbicide-resistant soybean. Scientia Agricultura Sinica. 2020, ; 53(6): 1166-1177. (in Chinese)

[2027] GAO Gao J-F. Plant Physiology Experiment Guide. 1st ed. Beijing: Higher Education Press, 2006.

[2128] Lesleigh FFORCE L, Christa C, Jack JS CRITCHLEY C, RENSEN J J S. New fluorescence parameters for monitoring photosynthesis in plants. Photosynthesis Research, 2003, ; 78(1): 17-33. doi: 10.1023/A:1026012116709

[2229] Mark AP, Alberto C, Ramiro O, Vinod S, Michael JW. The challenge of herbicide resistance around the world: a current summary. Pest Management Science, 2018, ; 74(10):2246-2259.

Formatted: Line spacing: Double

Formatted: Font: (Default) Times New Roman

Formatted: Font: (Default) Times New Roman

Formatted: Font: (Default) Times New Roman

Formatted: Line spacing: Double

Formatted: Font: (Default) Times New Roman

doi: 10.1002/ps.4821

[~~23~~30] ~~ZHAO~~ Zhao Y~~-~~N, ~~ZI~~ Zi X~~-~~J, Wang~~ANG~~ W, Feng~~ENG~~ Y, Yang~~ANG~~ P, ~~WANG~~ Wang P~~-~~K, ~~GAO~~ Gao X~~-~~L. Control effects of different herbicides on weeds as well as their effects on growth and development of broomcorn millet. Chinese Journal of Applied Ecology, 2020~~,~~; 31(7): 2415-2421. doi: 10.13287/j.1001-9332.202007.027 (in Chinese)

[~~24~~31] Prashant J, Vipan K, Josefina G, Nicholas R. Tank mixing pendimethalin with pyroxasulfone and chloroacetamide herbicides enhances in-season residual weed control in corn. Weed Technology, 2015~~,~~; 29(2):198-206. doi: 10.1614/WT-D-14-00095.1

[~~25~~32] Bradley CJ, Bryan G. Influence of temperature and relative humidity on the foliar activity of mesotrione. Weed Science, 2002~~,~~; 50(2): 157-161. doi: 10.1614/0043-1745(2002)050[0157:IOTARH]2.0.CO;2

[33] Yang P, Ren GY, Hou W, Liu WD. Spatial and diurnal characteristics of summer rainfall over Beijing Municipality based on a high-density AWS dataset. International Journal of Climatology, 2013; 33(13): 2769-2780. doi: 10.1002/joc.3622

[34] Beillouin D, Schauberger B, Bastos A, Ciais P, Makowski D. Impact of extreme weather conditions on European crop production in 2018. Philosophical Transactions of the Royal Society B: Biological Sciences, 2020; 375:20190510. doi: 10.1098/rstb.2019.0510

[35] Xiao C, Wu PL, Zhang LX, Song LC. Robust increase in extreme summer rainfall intensity during the past four decades observed in China. Scientific Reports, 2016; 6: 38506. doi: 10.1038/srep38506

[~~26~~36] Tim RH, Michael P L, Russell V, Rachael EB, Fiona MC, Elaine AM, et al. Characterization of 4-hydroxyphenylpyruvate dioxygenases, inhibition by herbicides and engineering for herbicide tolerance in crops. Pesticide Biochemistry and Physiology, 2019~~,~~; 156: 9-28. doi: 0.1016/j.pestbp.2019.01.006

[~~27~~37] ~~ZHANG~~ Zhang H~~-~~J, Liu~~IU~~ X, Ni~~I~~ H~~-~~W, Li~~I~~ X~~-~~J, Cui~~UI~~ H~~-~~L. Early diagnosis and mitigation

Formatted: Font: (Default) Times New Roman

Formatted: Font: (Default) Times New Roman

Formatted: Line spacing: Double

Formatted: Font: (Default) Times New Roman

methods of mesotrione on maize. Journal of Weed Science, 2009(4): 19-22. doi: 10.19588/j.issn.1003-935x.2009.04.005 (in Chinese)

[2838] GAO Gao X-X, LiI M, FangANG F, YuU T-C, LiuIU X-T. Interaction of clopyralid and mesotrione in maize field. Journal of Maize Sciences, 2015, ; 23(3): 143-148. doi: 10.13597/j.cnki.maize.science.20150324 (in Chinese)

[39] Spillman JJ. Spray impaction, retention and adhesion: An introduction to basic characteristics. Pest Management Science, 1984: 15(2): 97-106. doi: 10.1002/ps.2780150202

[2940] GUO Guo LEI, ZhangHANG B-B, ShenHEN J-H, HeE X, SONG Song H-F. Effects of glyphosate and paraquat on root morphology and aboveground growth of prunus persica seedlings. Chinese Journal of Applied Ecology, 2020, ; 31(2): 524-532. doi: 10.13287/j.1001-9332.202002.036 (in Chinese)

[3041] Mamiko S, Takahiro H, Yoshio S, Kangetsu H, Shinichi B. Action mechanism of bleaching herbicide cyclopyrimorate, a novel homogentisate solanesyltransferase inhibitor. Journal of Pesticide Science. 2018, ; 43(4):233-239. doi: 10.1584/jpestics.D18-008

[4231] FENGWEN ZHANGhang FW, Yao XIANGFENG YAO, SHIANG SUNun SA, LIPENG WANGang LP, Liu WEITANGWT LIU, XINGYIN JIANGiang XY, JINXIN WANGet al. Effects of mesotrione on oxidative stress, subcellular structure, and membrane integrity in Chlorella vulgaris. Chemosphere, 2020(247),): 125668. doi: 10.1016/j.chemosphere.2019.125668

[4332] ZHANG Zhang P-, JIANG Jiang X-Y, TanAN H-L, ShangHANG D-L, LiI X-D, ZHANG Zhang J-W. Effects of four spray adjuvants on the rainfastness of azoxystrobin on maize leaf surfaces and their effect on maize safety. Chinese Journal of Pesticide Science, 2018, ; 20(2): 239-248. doi: 10.16801/j.issn.1008-7303.2018.0032

[44] Lin FR, Huang XH, Chang H, Chen J. Herbicidal characteristics of a novel protoporphyrinogen oxidase inhibitor-Y11049. Chinese Journal of Pesticide Science, 2021; Forthcoming. doi:

Formatted: Line spacing: Double

Formatted: Font: (Default) Times New Roman

Formatted: Line spacing: Double

10.16801/j.issn.1008-7303.2021.0117 (in Chinese)

[3345] Sascha BS, Sebastiaan AM, Harm GG, Petra B, Dick Z, Dick V, et al. Laboratory algal bioassays using PAM fluorometry: Effects of test conditions on the determination of herbicide and field sample toxicity. Environmental Toxicology and Chemistry, 2014, ; 33: 1017-1022. doi: 10.1002/etc.2537

[3446] SHAO Shao G-Q, LiI Z-J, NingING T-Y, ZhengHENG Y-H. Responses of photo-synthesis, chlorophyll fluorescence, and grain yield of maize to controlled release urea and irrigation after anthesis. Journal of Plant Nutrition and Soil Science, 2013, ; 176: 595-602. doi: 10.1002/jpln.201100185

[3547] TSAI Tsai Y-C-, CHEN Chen K-C, ChengHENG T-S, LeeEE C, LinIN S-H, TungUNG C-W. Chlorophyll fluorescence analysis in diverse rice varieties reveals the positive correlation between the seedlings salt tolerance and photosynthetic efficiency. BMC Plant Biology, 2019, ; 19(1):403. doi: 10.1186/s12870-019-1983-8

[3648] YANG Yang H-J, YUAN Yuan X-Y, GuoUO P-Y, DongONG S-Q, ZHANhangG L-G, WEN Wen Y-Y, SONG et al.X E, WANG H F. Effects of brassinolide on photosynthesis, chlorophyll fluorescence characteristics and carbohydrates metabolism in leaves of foxtail millet under Sigma broad stress. Scientia Agricultura Sinica, 2017, 50(13): 2508-2518. (in Chinese)

[3749] YANG Yang X-F, SUN Sun P, SUN Sun D-S, SongONG X-E, DongONG S-Q, YUuanAN X Y, GUO P Yet al. Safety evaluation of allelochemical derivative pyrone on different millet varieties. Chinese Journal of Applied Ecology, 2020, 1(7): 2236-2242. doi: 10.13287/j.1001-9332.202007.005 (in Chinese)

[3850] GUO Guo M J, BAaiI Y-Q, GAO Gao P, ShenHEN J, DongONG S-Q, YuanUAN X-Y, GUO P Yet al. Effect of MCPA on leaf senescence and endogenous hormones content in leaves of foxtail millet seedlings. Scientia Agricultura Sinica, 2020, 53(3): 513-526. (in Chinese)

[51] Li JJ, Zhang YH, Wang BR, Wang LC, Tao B. Synergistic Effect of Different Adjuvants on

Formatted: Font: (Default) Times New Roman

Formatted: Font: (Default) Times New Roman

Atrazine in Maize Field. Journal of Maize Sciences, 2019; 27(6): 167-174. doi: 10.13597/j.cnki.maize.science.20190626 (in Chinese)

[52] Tao B, Zhang YH, Tian LJ, Zheng CY, Li SY, Kong LW. Effects of adjuvants on spray droplet distribution of herbicides sprayed by plant protection UAV. Plant Protection, 2021; 47(1): 108-116. doi: 10.16688/j.zwbh.2019558 (in Chinese)

Dear Editors and Reviewers:

Thank you for your letter and for the reviewers' comments concerning our manuscript entitled "**Effects of mesotrione on the control efficiency and chlorophyll fluorescence parameters of *Chenopodium album* under simulated rainfall conditions**". Those precious comments are all valuable and very helpful for revising and improving our paper, as well as the important guiding significance to our researches. We have studied comments carefully and have made correction which we hope meet with approval. Revised portion are marked in red in the paper. The main corrections in the paper and the responds to the reviewer's comments are as flowing:

Responds to the reviewer's comments:

Reviewer #1:

1. Response to comment: The idea is good and seems novel but study was conducted only one year (4 months) on weed only

Response: The experiment was mainly conducted in the field, and we tested it again in the greenhouse. Ultimately, the conclusion we drew in the field and the greenhouse were almost consistent. These are some of the photos from our greenhouse experiments. The following figures show the influence of different intervals under the rain intensity of 2, 6 and 10 mm/h, with 0.5 h, 1 h, 2 h and 4 h intervals from top to bottom.

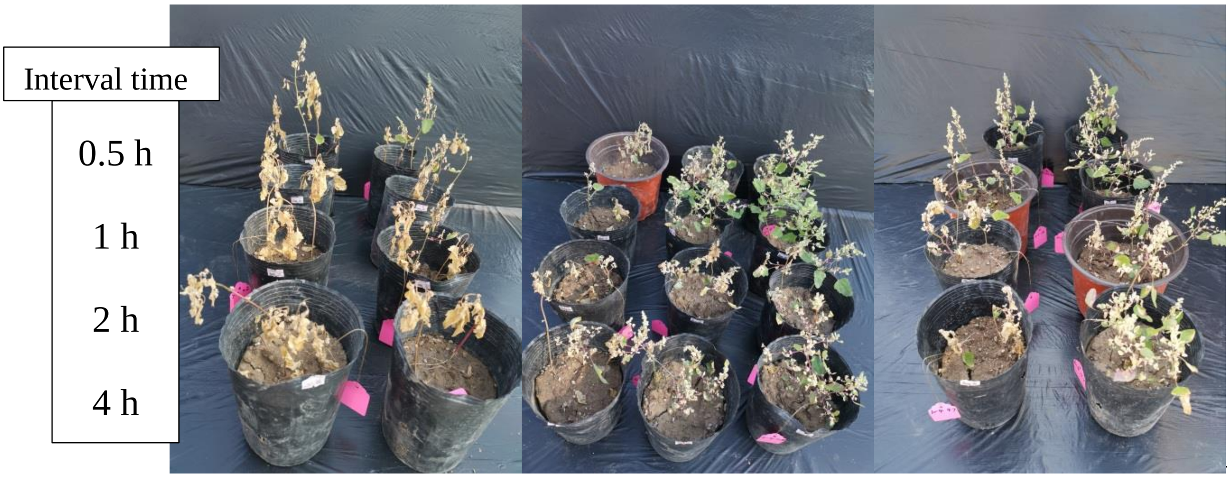

2. Response to comment: Whereas, the situation in entirely different under field condition due to both crop and weeds.

Response: This question is very good. As for the effect of mesotrione on safety of crops in normal weather condition, our laboratory has conducted research in the previous stage and made sure that mesotrione can be used in foxtail millet field. And we analyzed those

experimental results by combine agronomic traits and physiological indexes and found the safe dosage of mesotrione in foxtail millet field is 75-300 g/hm^2. Other studies have shown that mesotrione has the best control effect on *Chenopodium* in broad-leaved weeds, so we chose *Chenopodium* as the research object. Combined with the background of frequent extreme weather, this experiment mainly studied the influence of rainfall intensity and time interval on the effect of mesotrione on *Chenopodium*. This study can help people to understand how rainfall affect the weed control efficiency of the herbicide. Due to space and content limitations, we only present the part results in this paper. As for the effect of mesotrione on the other weeds and the crop, we will present the results in another paper or make a further study.

Reference：

[1] Shi Guodong, Foliar application of mesotrione to different millet varieties safety study. Taigu: Shanxi Agricultural University. 2019.

[2] Wang JP, Liu XM, Xu X, *et al*. Control Effects of Seven Herbicides on Weed in Summer Maize Fields and Their Safety. Journal of Hebei Agricultural Sciences, 2018, 22(1): 50-53. (in Chinese)

3. Response to comment: Introduction needs improvement: information on *Chenopodium* yield loss, and morphological behaviour is required.

Response: As for the reviewer’s concern, we have added information on *Chenopodium* yield loss, and morphological behavior in the introduction. Please check and review the part of Introduction.

4. Response to comment: Some of the important observation may be included in the results as I commented.

Response: We are appreciative of the reviewer’s suggestion. According to the previous experimental photos and datas, we have made a careful observation again and graded the harmful symptoms of *Chenopodium*. Some of the important observation have been added in the “3.1 The visual control effect ” of Results, please review again.

Another comments on mailbox:

1. **Response to comment:** Title: Please correct spelling of “conditions”

   Response: I have modified this word in our title.

2. **Response to comment:** The authors mentioned the rainfall intensities and intervals

investigated without any context of how do these treatments hold relevance in the local climate systems.

Response: Thank you for your comments. In this article, we added the rainfall intensity of five months during the experiment, and found that light, medium and heavy rainfall intensity will occur in the summer rainfall. Summer is the key period for crop growth and weeding, but the herbicide would reduce the efficacy due to the rainfall scouring. What rainfall intensity has a great impact on the efficacy of mesotrione, and whether to be treated again. The influence of rain intensity and interval time on the efficacy was determined, which provide a theoretical basis for the use of mesotrione.

Special thanks to you for your good comments, these are very helpful to our article.

Reviewer #2:

1. Response to comment: Line no. 14 space

Response: We are very sorry for our negligence, we have made correction according to the Reviewer's comments.

2. Response to comment: Kindly mention control efficiency reduced how much percentage?

Response: The percentage of control efficiency reduction has been supplemented in the Summary and Conclusion. "At 6 mm/h within 1 h after treatment, the control effect of fresh weight was significantly reduced by more than 7.14%, and at 10 mm/h within 2 h, the control effect was significantly reduced by more than 14.78%". Please review. Thanks so much for your useful comments.

3. Response to comment: Line no. 37 rewrite the sentence.

Response: As for the reviewer's concern, we have re-written this sentence in line 57-58. The new sentence is "Weed would reduce the yield and quality of the crops, hinder mechanized sowing and harvest and then slow down the process of agricultural modernization"

4. Response to comment: Give at-least one example how much yield loss? What is the importance of *Chenopodium* weed, its biology, and morphological behavior could better explain the results.

Response: We are very sorry for our unclear report in this manuscript, we have made additions and changes in the Introduction, please review.

5. Response to comment: Mention its chemical content and concentration

Response: We choose 10% mesotrione emulsifiable concentrate (EC) as our herbicide, we added this part in Materials and methods .

6. Response to comment: Line no. 73 materials and methods the number may be given 2

Response: We have made correction according to the Reviewer's comments, the full text has been modified in this way.

7. Response to comment: How you controlled the rainfall intensity, duration under field condition. Also drift effect is common under field condition.

Response: Because the weather is changeable in summer, so the different intensity of the short interval rainfall like light, moderate, heavy rainfall will all appear. According to the standard of China Meteorological Administration and the rainfall circumstance of TaiGu, we simulated the rainfall intensities were light rain 2 mm/h, moderate rain 6 mm/h and heavy rain 10 mm/h. Before the test in the field, we conducted many experiments about rainfall simulation. Firstly, we screened out one type of shower that sprayed the smallest water and most homogeneous when spraying. We also controlled the moving speed of spray lance by ourselves and found it is equal to the rainfall whose intensity is 2、6、10 mm/h when the capable of spraying was 4, 12 and 20 L per half an hour. According to the means and data of the preliminary experiment, we chose a windless and sunny day in the field experiment, and we put many baffles around the rainfall simulation areas of the neighborhood like the preliminary experiment. The simple facilities that put up by us when we test the rainfall intensity are as follows.

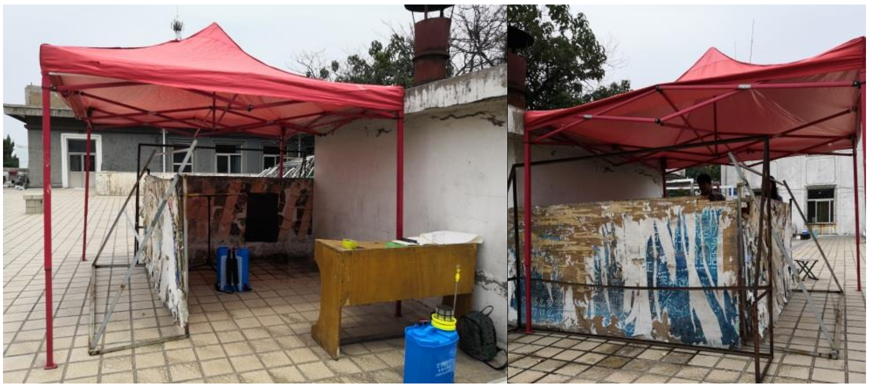

8. Response to comment: The experiment was conducted for one season only (4 months), whereas repeated results can explain the cause and effects better.

Response: The experiment was mainly conducted in the field, and we tested it again in the

greenhouse. Ultimately, the conclusion we drew in the field and the greenhouse were almost consistent. These are some of the photos from our greenhouse experiments. The following figures show the influence of different intervals under the rain intensity of 2, 6 and 10 mm/h, with 0.5 h, 1h, 2 h and 4 h intervals from top to bottom.

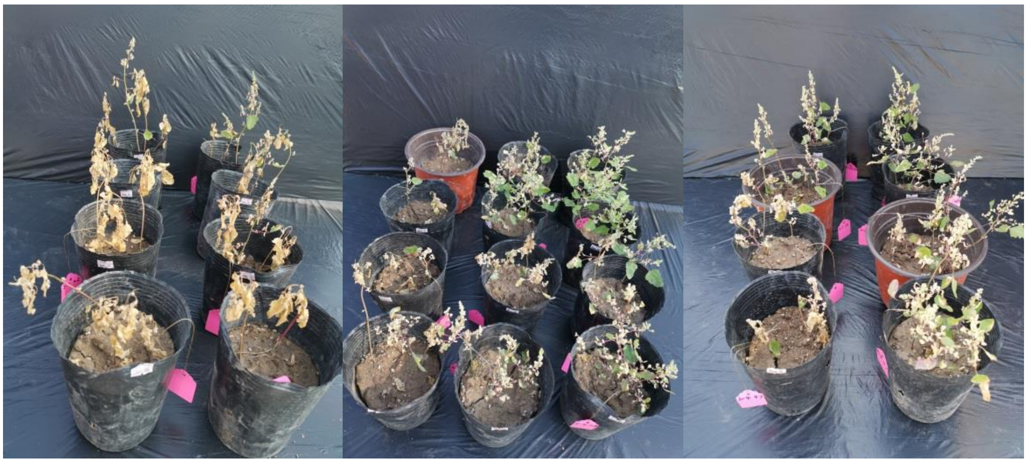

9. Response to comment: Line 86: manual or power operated sprayer

Response: We choose power operated sprayer in our experience.

10. Response to comment: Line 92: How artificial spraying mimicked the natural rain, what is the height of spraying on the weed. If you have picture just present for clear understanding.

Response: Before the start of the field test, we conduct an experiment for two months when the intensity and the precipitation time were suitable. The concrete operations are as follows： when spraying and mark the appropriate place in the electric spray knob by many tests, screening out one type of shower that sprayed the smallest water and most homogeneous, and we moved the spray lance in a constant speed within the unit area. The electric sprayer was continuously adjusted to the appropriate site according to the water yield, so that the water yield of 0.5 h was 4 L, 12 L and 20 L, reaching the rain intensity standard of 2, 6 and 10mm/h. The error is maintained at ±0.2 mm/h. The height of the spray is 0.5 meters, mark the wall in advance, such as the position of the green line in the last picture. In the newly submitted manuscript, we have rewritten the Materials and methods.

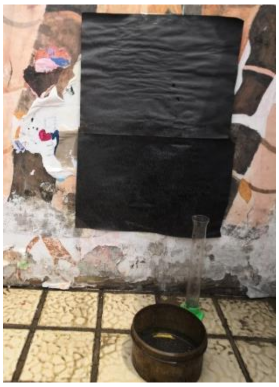
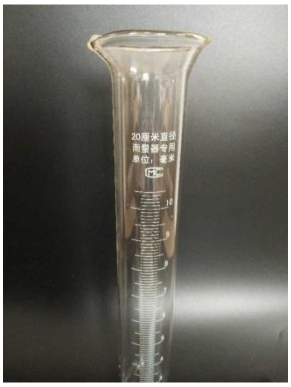
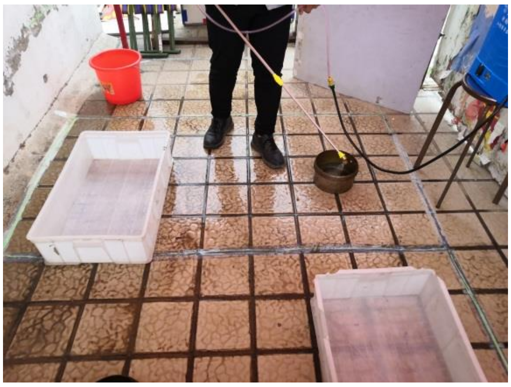
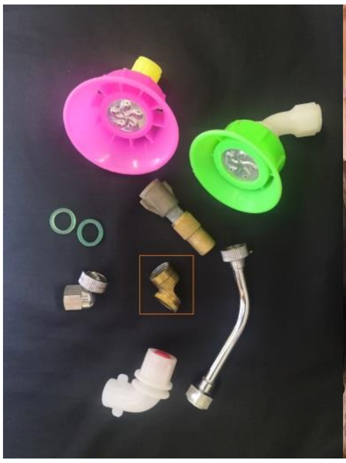
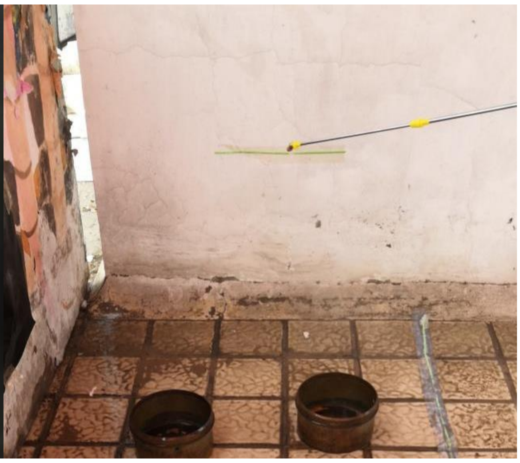

11. **Response to comment:** Only *Chenopodium* plans were measured? What about remaining weed species?

Response: *Chenopodium* is easy to survive under any bad existent conditions. It competes with crops on the water and nutrition, so the yield and the quality of crops sharply drop to a low level. And then the agriculture suffers massive loss. The damage that is caused by *Chenopodium* interference has been well documented in field crops. *Chenopodium* has been reported to reduce the barley (*Hordeum vulgare* L.) yield by 36%[1]. In sugarbeet (*Beta vulgaris* L.) field, the yield was reduced by 48%, which was associated with interference of this weed[2]. The presence of *Chenopodium* in corn fields has been shown to reduce the yield by 58%, and the yield of soybean will drop by 61% in the severe cases[3]. *Chenopodium* are always companied by the mixed agriculture pattern of millet (both foxtail millet and broomcorn millet), rice and wheat[4]. The experiment was carried out in foxtail millet field, *Chenopodium* is one of major weeds, so we did this experiment that focused on the *Chenopodium*. Of course, other weeds can also be targeted for future research, we will continue our study, thanks for your advice!

Reference:

[1] Conn JS, Thomas DL. Common lambsquarters (*Chenopodium album*) interference in spring

barley. Weed Technology. 1987(1): 312–313.

[2] Schweizer EE. Common lambsquarters (*Chenopodium album*) interference in sugarbeets (Beta vulgaris). Weed Science. 1983; 31(1):5 – 7.

[3] Conley SP, Stoltenberg DE, Boerboom CM, Binning LK. Predicting soybean yield loss in giant foxtail (*Setaria faberi*)a n d common lambsquarters (*Chenopodium album*) communities. Weed Science. 2003; 51(3): 402–407.

[4] Gao YY. Research on *Chenopodium* in ancient southwest China. IOP Conference Series Earth and Environmental Science, 2021; 621: 012124.

12. Response to comment: What is the plot size?

Response: As the article says, the plot size is 4 m^2 (2 m × 2 m). Here are some pictures of our experiment.

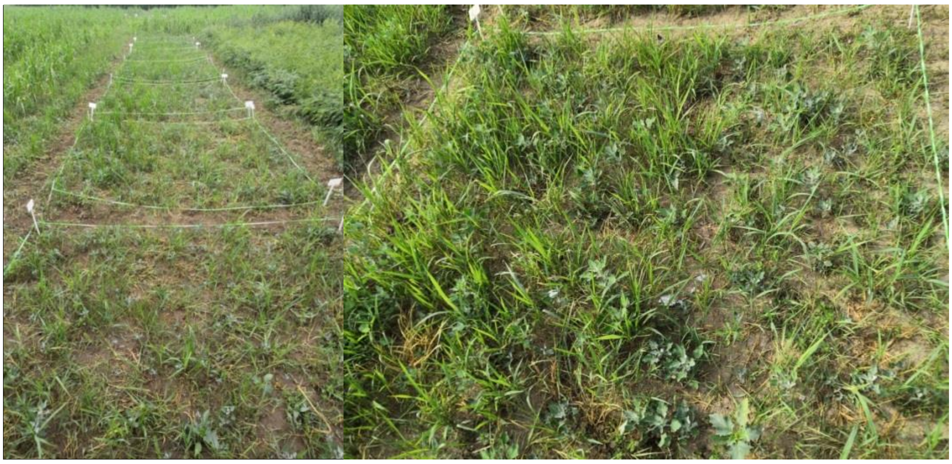

Response: I have carefully modified every figure according to the comments of the reviewer.

13. Response to comment: Study conducted in open field or green house conditions?

Response: This study was conducted in open field, but we did a confirmatory experiment in greenhouse.

14. Response to comment: Line 136: weed may be removed

Response: As Reviewer suggested that we have changed this part.

15. Response to comment: Line no. 137: mention disease like

Response: We agree with the reviewer and corrected accordingly, to make it more informative and read more smoothly.

16. Response to comment: Line no. 203: 4th

Response: We are very sorry for the mistakes in this manuscript and inconvenience they

caused in your reading. The manuscript has been thoroughly revised.

17. Response to comment: Results are clearly explained but, Herbicide effect was studied on weed under sole weed cultivation.

Response: *Chenopodium* is listed among the most common dicotyledonous weeds in the world and it's widely distributed in many agricultural areas, which severely reduce the yield and decrease the quality of the crops[1]. Because *Chenopodium* is one of the main weeds in the foxtail millet field and it is flooding in the drought areas, so we choose *Chenopodium* as our primary research object[2]. In order to show that the efficacy of mesotrione to weeds under different intensity, we use *Chenopodium* as the only research object in the experiment. We can regard the other weeds as a research in the following experiment period.

Reference:

[1] Moghadam S H, Alebrahim M T, Tobeh A, *et al*. Redroot Pigweed (*Amaranthus retroflexus* L.) and Lamb's Quarters (*Chenopodium album* L.) Populations Exhibit a High Degree of Morphological and Biochemical Diversity. Frontiers in Plant Science, 2021, 12: 593037.

[2]Song Xie, Guo Haoxuan, LiuYanan, et al. Investigation and Analysis on Occurrence of Weeds in Spring Sowing Millet Fields in Jinzhong City, Shanxi Province. Journal of Weed Science, 2020, 38(2): 9-15.(in Chinese)

18. Response to comment: But the response of herbicide would be different in real (mixed) condition with crop plants). Hence combined plant stand would explain better.

Response: We understand that combined crop plant stand would better. However, my senior schoolmates who study in our laboratory, he did some research about how much mesotrione would be safe or harmful for the foxtail millet. He found that the mesotrione is suitable for the foxtail millet field under the dose about 75-300 g/hm^2. Because the weather in summer is changeable and rainy days are common here, so we had a further experiment to study if the rainwash would have an effect on the mesotrione and what effect would have. Then we can hope to screen out a safe and effective herbicide. In the present study, we mainly focused on the rainwash of the mesotrione and regarded *Chenopodium* as an assistant research object. We think that study under sole weed may not be optimal, but should be sufficient to draw my conclusion about the effect of rainfall intensity on mesotrione. As for the influence in the mixed condition, we will make further in-depth study, many thanks for your kind help!

19. Response to comment: No data on *Chenopodium* leaf area, since LA is the most important factor decides the efficacy of herbicide.

Response: Thank you for your instructive suggestions. We are sorry for not describing the data on *Chenopodium* leaf area in our manuscript. All leaf area were conducted statistical analyses by Image J, we found that the leaf area of the treatment was between 26.7 to 39.4 cm^2.

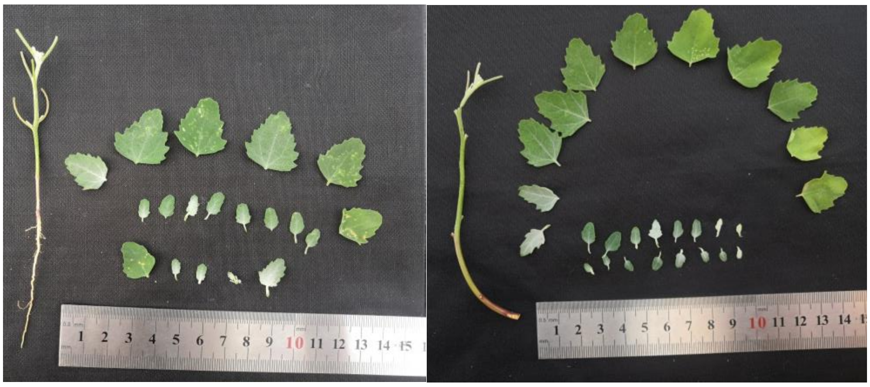

20. Response to comment: What is the retention of herbicide concentration on weed leaves?

Response: I'm very apologetic for I didn't mention the residual quantity of the herbicide in my article, but I really agree with the reviewer's suggestions about my article. The retention of herbicide concentration on weed leaves will have an impact on the efficacy of mesotrione straightly. This experiment mainly studied the influence of rainfall intensity and time interval on the effect of mesotrione on *Chenopodium*. We are concentrating on the control effect and the physiological indexes of *Chenopodium*. This only is a pretty elementary research result, then we will conduct more studies about the leaves residual etc. Thanks for your valuable advice!

21. Response to comment: How much of applied herbicide was lost?

Response: I'm so sorry. We didn't measure the residual of the herbicide before or after the rain fall. But we calculate how much the efficacy of herbicide dropped by comparing the control effect of mesotrione on *Chenopodium* under different rainfall intensity. Because we chose plants that have similar size, the reduction of the plant height inhibition rate, the plant control effect, fresh weight control effect could show the loss of the mesotrione from the another side.

When the rain intensity was 2 mm/h, the plant height inhibition rate, plant control efficiency and fresh weight control efficiency of *Chenopodium* at intervals of 0.5 h and 4 h decreased by 18.07%, 18.12%, 16.25%, 4.86%, 1.50% and 2.01% respectively at 15 DAT. So we can infer that the loss of the herbicide is between 1.50% and 18.12%. In the same way, the weed control loss of

the herbiside between 16.02% and 48.25%. when the rainfall intensity is 6 mm/h. And the loss is between 17.38% and 52.82% when the rainfall intensity is 10 mm/h, and we have added these datas in the new manuscript. As for the detail about herbicide loss, we will make a further study. Thanks for your meaningful comments!

22. Response to comment: Discussion is fine with the supporting literature. However, your results could have discussed more, for example time of rain interval, all rain intensities and interaction effect, what extent it affected.

Response: Your comments were highly insightful and enabled us to greatly improve the quality of our manuscript. We have re-written Discussion according to the Reviewer's suggestion. We hope that the correction will meet with approval, thank you very much for your comments and suggestions.

23. Response to comment: Discussion section is lacking the supporting data on herbicide retention on leaves, leaching loss, biomass reduction.

Response: I agree with you ! Herbicide retention on leaves, leaching loss, biomass reduction are important datas what could show the efficacy of the mesotrione straightly. We only explore the control effect of mesotrione and the physiological indexes on *Chenopodium* under different rainfall intensity, this is a pretty basical research. We have konwn the loss and the residual of the herbiside are closely related to the efficacy. Hence, our group is going to carry out further experiments.

In addition to the above commernts, there is a comment regarding data in Table 3.

1. Response to comment: I think treatments comparison was done for T1 to T4 within the rainfall intensities. Whereas, comparison between all the treatment combinations (3*5= 15) would be good.

Response: I also think comparison between all the treatment combinations (3*5= 15) is a good idea. But in our experiment, we could intuitively observe that the efficacy loss of the mesotrione will be serious when the rainfall intensity was large. And the emphasis of this experiment was comparing the effect of rainfall interval to the efficacy of mesotrione under different rainfall intensity. Hence in order to draw a nice conclusion, we compared them separately.

We tried our best to improve the manuscript and made some changes in the manuscript. These changes will not influence the content and framework of the paper. And here we did not list

the changes but marked in red in revised paper. We appreciate for Reviewers' warm work earnestly and hope that the correction will meet with approval. Once again thank you very much for your comments and suggestions.

In all, I found the reviewer's comments are quite helpful, and I revised my paper point-by-point. Thank you and the review again for your help.
