# Supplementary material for: Effects of mesotrione on the control efficiency and chlorophyll fluorescence parameters of Chenopodium album under simulated rainfall conditions
Source: PLoS One. 2022 Jun 3;17(6):e0267649. doi: 10.1371/journal.pone.0267649 (PMC9165882; doi:10.1371/journal.pone.0267649)
Supplement: Attachment — Submitted filename: Comments to editor.docx [file pone.0267649.s007.docx]

**Dear PLOS ONE,**

I have reviewed the manuscript "Effects of mesotrione on the control efficiency and chlorophyll fluorescence parameters of *Chenopodium* under simulated rainfall conditions by Mengmeng Sun, Meijun Guo, Shuai Guo, Yanfen Li, Shuqi Dong, Sie Song, Xiangyang Yuan. I tried my level best to comment critically and impartially on the article. Some of the more critical points I observed and commented as per my idea.

I recommend this article for further process after attending the comments (major modification) given to author.

**Some of the concerns are;**

- The idea is good and seems novel but study was conducted only one year (4 months) on weed only.
- Whereas, the situation in entirely different under field condition due to both crop and weeds.
- Introduction needs improvement: information on chenopodium yield loss, and morphological behaviour is required.
- Some of the important observation may be included in the results as I commented.

The final decision may be taken by the editor.

Finally express my gratitude to editor for giving this opportunity to review article which may be a good exposure for me in this field.

Thanking you,

**Yours**

Dr. H. M. Halli

Scientist (Agronomy), Division of Seed Technology

ICAR- Indian Grassland and Fodder Research Institute

Jhansi, India-284003.
