# Supplementary material for: Effects of mesotrione on the control efficiency and chlorophyll fluorescence parameters of Chenopodium album under simulated rainfall conditions
Source: PLoS One. 2022 Jun 3;17(6):e0267649. doi: 10.1371/journal.pone.0267649 (PMC9165882; doi:10.1371/journal.pone.0267649)
Supplement: Attachment — Submitted filename: table.docx [file pone.0267649.s009.docx]

Table 1 The basic physical and chemical properties of soil

| Depth of soil layer | pH | Available potassium(mg/kg) | Available phosphorus(mg/kg) | Available nitrogen(mg/kg) | organic matter(g/kg) |
| --- | --- | --- | --- | --- | --- |
| 0-5 cm | 8.21 | 484.21 | 43.70 | 64.20 | 8.52 |
| 5-10 cm | 8.14 | 471.93 | 26.79 | 42.80 | 8.52 |
| 10-15 cm | 8.17 | 319.60 | 21.28 | 49.93 | 7.99 |

Table 2 Effect of simulated rainfall on the weed control of mesotrione

| Rainfall Intensity | International time | Plant height inhibition rate/% | |  | Plant control efficacy/% | |  | Fresh weight control effect/% | | |  |
| --- | --- | --- | --- | --- | --- | --- | --- | --- | --- | --- | --- |
|  |  | 15 d | 30 d |  | 15 d | 30 d |  | | 15 d | 30 d | |
| 2 mm·h^-1^ | CK | 65.29±1.81a | 84.29±1.14a |  | 83.09±1.78a | 96.42±3.39a |  | | 81.91±2.71a | 96.67±3.01a | |
|  | T1 | 53.49±2.10b | 80.79±2.11a |  | 68.03±4.22b | 91.20±5.12a |  | | 68.60±2.74b | 93.89±2.83a | |
|  | T2 | 54.42±1.00b | 81.85±3.06a |  | 71.26±3.73b | 93.68±2.80a |  | | 70.53±5.82b | 94.39±2.85a | |
|  | T3 | 55.59±1.95b | 82.37±2.24a |  | 77.92±5.58ab | 95.32±1.37a |  | | 76.45±3.67ab | 95.28±3.54a | |
|  | T4 | 62.12±2.68a | 83.94±1.89a |  | 81.84±3.38ab | 96.08±7.82a |  | | 80.26±4.78a | 95.74±2.53a | |
| 6 mm·h^-1^ | CK | 65.29±1.81a | 84.29±1.14a |  | 83.09±1.78a | 96.42±3.39a |  | | 81.91±2.71a | 96.67±3.01a | |
|  | T1 | 41.19±1.42c | 78.72±2.67b |  | 43.00±4.39c | 76.98±4.01c |  | | 51.16±4.82c | 80.33±3.20c | |
|  | T2 | 43.27±2.06c | 81.02±1.84ab |  | 56.69±2.46b | 85.21±7.30b |  | | 54.92±4.39c | 89.77±6.00b | |
|  | T3 | 50.71±1.72b | 82.14±2.46a |  | 63.30±3.94b | 89.50±5.51b |  | | 64.42±3.43b | 94.28±3.68a | |
|  | T4 | 54.83±1.16b | 83.44±1.81a |  | 65.73±2.37b | 90.41±4.72b |  | | 68.74±4.01b | 96.28±2.15a | |
| 10 mm·h^-1^ | CK | 65.29±1.81a | 84.29±1.14a |  | 83.09±1.78a | 96.42±3.39a |  | | 81.91±2.71a | 96.67±3.01a | |
|  | T1 | 40.32±2.38c | 76.76±1.98c |  | 39.20±3.63c | 68.96±3.63d |  | | 43.09±2.45c | 77.37±2.12c | |
|  | T2 | 42.86±1.61bc | 79.38±2.93bc |  | 42.96±1.92c | 78.66±2.51c |  | | 55.12±5.23b | 88.20±4.31b | |
|  | T3 | 47.16±1.05bc | 82.12±1.30ab |  | 55.90±2.61bc | 82.17±1.53c |  | | 57.16±4.23b | 89.87±3.39b | |
|  | T4 | 53.94±0.99b | 82.36±1.28ab |  | 65.46±1.17b | 87.68±3.43b |  | | 62.08±3.62b | 94.09±4.09a | |

Note：Data are shown as mean ±SE with the different lowercase letters in the same column represent significant differences (P<0.05),the same as follows..

| Rainfall Intensity | International time | Chlorophyll a content(mg g^-1^ FW) | | |  | | | Chlorophyll b content (mgg^-1^ FW) | | |  | | | Chlorophyll a+b content(mg g^-1^ FW) | | | | |  |  |
| --- | --- | --- | --- | --- | --- | --- | --- | --- | --- | --- | --- | --- | --- | --- | --- | --- | --- | --- | --- | --- |
|  |  | 3 d | 6 d |  | | 3 d | | | 6 d | | |  | | | 3 d | | 6 d | | |  |
| 2 mm·h^–1^ | CK | 0.9167±0.0578b | 0.7900±0.1012a |  | | 0.2733±0.0203c | | | 0.2633±0.0273a | | |  | | | 1.1900±0.0400c | | 1.0533±0.0751b | | |  |
|  | T1 | 1.3900±0.0208a | 1.0000±0.0781a |  | | 0.4633±0.0120a | | | 0.3467±0.0617a | | |  | | | 1.8533±0.0120a | | 1.3467±0.0186a | | |  |
|  | T2 | 1.0567±0.0982b | 0.9000±0.0493a |  | | 0.3833±0.0120b | | | 0.3067±0.0296a | | |  | | | 1.4400±0.0866b | | 1.2067±0.0203ab | | |  |
|  | T3 | 1.0933±0.0736b | 0.8233±0.0736a |  | | 0.3400±0.0231b | | | 0.3200±0.0265a | | |  | | | 1.4333±0.0536b | | 1.1433±0.0484b | | |  |
|  | T4 | 0.9500±0.0404b | 0.7867±0.0448a |  | | 0.3500±0.0208b | | | 0.2800±0.0252a | | |  | | | 1.3000±0.0300bc | | 1.0667±0.0689b | | |  |
| 6 mm·h^–1^ | CK | 0.9167±0.0578b | 0.7900±0.1012b |  | | | 0.2733±0.0203c | | | 0.2633±0.0273b | | |  | | | 1.1900±0.0400c | | 1.0533±0.0751b | | |
|  | T1 | 1.2900±0.0745a | 1.1833±0.0775a |  | | | 0.5100±0.0208a | | | 0.4667±0.0088a | | |  | | | 1.8000±0.0100a | | 1.6500±0.0862a | | |
|  | T2 | 1.2433±0.0306a | 1.1400±0.0666a |  | | | 0.3700±0.0351b | | | 0.4600±0.0115a | | |  | | | 1.5667±0.0348b | | 1.6000±0.0551a | | |
|  | T3 | 1.1967±0.0384a | 1.1433±0.1268a |  | | | 0.3467±0.0203bc | | | 0.4167±0.0176a | | |  | | | 1.5900±0.0557b | | 1.5600±0.1106a | | |
|  | T4 | 0.9833±0.0498b | 0.9700±0.0513ab |  | | | 0.3533±0.0371bc | | | 0.3133±0.0549b | | |  | | | 1.3367±0.0088c | | 1.2833±0.0145b | | |
| 10 mm·h^–1^ | CK | 0.9167±0.0578c | 0.7900±0.1012b |  | | | 0.2733±0.0203c | | | 0.2633±0.0273c | | |  | | | 1.1900±0.0400c | | 1.0533±0.0751b | | |
|  | T1 | 1.3333±0.0636a | 1.2067±0.0788a |  | | | 0.5300±0.0361a | | | 0.5100±0.0306a | | |  | | | 1.8633±0.0991a | | 1.7167±0.0713a | | |
|  | T2 | 1.3433±0.0639ab | 1.1933±0.0410a |  | | | 0.4033±0.0033b | | | 0.4900±0.0208a | | |  | | | 1.7467±0.0669a | | 1.6833±0.0555a | | |
|  | T3 | 1.1367±0.0203ab | 1.1600±0.1127a |  | | | 0.3767±0.0219b | | | 0.4067±0.0260b | | |  | | | 1.5133±0.0410b | | 1.5667±0.1387a | | |
|  | T4 | 1.1833±0.0825b | 1.1533±0.0504a |  | | | 0.3567±0.0348b | | | 0.3733±0.0186b | | |  | | | 1.5400±0.0503b | | 1.5267±0.0367a | | |

Table 3 Effect of simulated rainfall on photosynthetic pigment content in leaves of Chenopodium treated by mesotrione
