# Supplementary material for: Effects of mesotrione on the control efficiency and chlorophyll fluorescence parameters of Chenopodium album under simulated rainfall conditions
Source: PLoS One. 2022 Jun 3;17(6):e0267649. doi: 10.1371/journal.pone.0267649 (PMC9165882; doi:10.1371/journal.pone.0267649)
Supplement: Attachment — Submitted filename: Comments to author.docx [file pone.0267649.s010.docx]

**Comments to author**

**Dear Author,**

I have reviewed the manuscript "Effects of mesotrione on the control efficiency and chlorophyll fluorescence parameters of *Chenopodium* under simulated rainfall conditions by Mengmeng Sun, Meijun Guo, Shuai Guo, Yanfen Li, Shuqi Dong, Sie Song, Xiangyang Yuan. The main objective of the study is to study the effect of rainfall intensity and time interval on the efficacy of mesotrione. The study seems well planned data collection and analysis.

**Some of the more critical points that I observed were:**

- Line no. 14 space
- Kindly mention control efficiency reduced how much percentage?
- Line no. 37 rewrite the sentence
- In introduction: Give at-least one example how much yield loss? What is the importance of Chenopodium weed, its biology, and morphological behavior could better explain the results.
- Mention its chemical content and concentration
- Line no. 73 materials and methods the number may be given 2
- How you controlled the rainfall intensity, duration under field condition. Also drift effect is common under field condition.
- The experiment was conducted for one season only (4 months), whereas repeated results can explain the cause and effects better.
- Line 86: manual or power operated sprayer
- Line 92: How artificial spraying mimicked the natural rain, what is the height of spraying on the weed. If you have picture just present for clear understanding
- Only chenopodium plans were measured? What about remaining weed species?
- What is the plot size?
- Study conducted in open field or green house conditions?
- Line 136: weed may be removed
- Line no. 137: mention disease like
- Line no. 203: 4^th^
- Results are clearly explained but, Herbicide effect was studied on weed under sole weed cultivation.
- But the response of herbicide would be different in real (mixed) condition with crop plants). Hence combined plant stand would explain better.
- No data on chenopodium leaf area, since LA is the most important factor decides the efficacy of herbicide.
- What is the retention of herbicide concentration on weed leaves?
- How much of applied herbicide was lost?
- Discussion is fine with the supporting literature. However, your results could have discussed more, for example time of rain interval, all rain intensities and interaction effect, what extent it affected.
- Discussion section is lacking the supporting data on herbicide retention on leaves, leaching loss, biomass reduction.
