# Supplementary material for: Effects of mesotrione on the control efficiency and chlorophyll fluorescence parameters of Chenopodium album under simulated rainfall conditions
Source: PLoS One. 2022 Jun 3;17(6):e0267649. doi: 10.1371/journal.pone.0267649 (PMC9165882; doi:10.1371/journal.pone.0267649)
Supplement: Attachment — Submitted filename: Response to Reviewers.docx [file pone.0267649.s011.docx]

Dear Editors and Reviewers:

Thank you for your letter and for the reviewers’ comments concerning our manuscript entitled “**Effects of mesotrione on the control efficiency and chlorophyll fluorescence parameters of *Chenopodium* *album* under simulated rainfall conditions**”. Those precious comments are all valuable and very helpful for revising and improving our paper, as well as the important guiding significance to our researches. We have studied comments carefully and have made correction which we hope meet with approval. Revised portion are marked in red in the paper. The main corrections in the paper and the responds to the reviewer’s comments are as flowing:

Responds to the reviewer’s comments:

**Reviewer #1:**

**1. Response to comment:** The idea is good and seems novel but study was conducted only one year (4 months) on weed only

**Response:** The experiment was mainly conducted in the field, and we tested it again in the greenhouse. Ultimately, the conclusion we drew in the field and the greenhouse were almost consistent. These are some of the photos from our greenhouse experiments. The following figures show the influence of different intervals under the rain intensity of 2, 6 and 10 mm/h, with 0.5 h, 1 h, 2 h and 4 h intervals from top to bottom.

Interval time

0.5 h

1 h

2 h

4 h

**2. Response to comment:** Whereas, the situation in entirely different under field condition due to both crop and weeds.

**Response:** This question is very good. As for the effect of mesotrione on safety of crops in normal weather condition, our laboratory has conducted research in the previous stage and made sure that mesotrione can be used in foxtail millet field. And we analyzed those experimental results by combine agronomic traits and physiological indexes and found the safe dosage of mesotrione in foxtail millet field is 75-300 g/hm^2^. Other studies have shown that mesotrione has the best control effect on *Chenopodium* in broad-leaved weeds, so we chose *Chenopodium* as the research object. Combined with the background of frequent extreme weather, this experiment mainly studied the influence of rainfall intensity and time interval on the effect of mesotrione on *Chenopodium*. This study can help people to understand how rainfall affect the weed control efficiency of the herbicide. Due to space and content limitations, we only present the part results in this paper. As for the effect of mesotrione on the other weeds and the crop, we will present the results in another paper or make a further study.

**Reference：**

[1] Shi Guodong, Foliar application of mesotrione to different millet varieties safety study. Taigu: Shanxi Agricultural University. 2019.

[2] Wang JP, Liu XM, Xu X, *et al*. Control Effects of Seven Herbicides on Weed in Summer Maize Fields and Their Safety. Journal of Hebei Agricultural Sciences, 2018, 22(1): 50-53. (in Chinese)

**3.** **Response to comment:** Introduction needs improvement: information on *Chenopodium* yield loss, and morphological behaviour is required.

**Response:** As for the reviewer’s concern, we have added information on *Chenopodium* yield loss, and morphological behavior in the introduction. Please check and review the part of Introduction.

**4. Response to comment:** Some of the important observation may be included in the results as I commented.

**Response:** We are appreciative of the reviewer’s suggestion. According to the previous experimental photos and datas, we have made a careful observation again and graded the harmful symptoms of *Chenopodium*. Some of the important observation have been added in the “3.1 The visual control effect ” of Results, please review again.

Another comments on mailbox:

1. **Response to comment:** Title: Please correct spelling of “conditions”

**Response:** I have modified this word in our title.

2. **Response to comment:** The authors mentioned the rainfall intensities and intervals investigated without any context of how do these treatments hold relevance in the local climate systems.

**Response:** Thank you for your comments. In this article, we added the rainfall intensity of five months during the experiment, and found that light, medium and heavy rainfall intensity will occur in the summer rainfall. Summer is the key period for crop growth and weeding, but the herbicide would reduce the efficacy due to the rainfall scouring. What rainfall intensity has a great impact on the efficacy of mesotrione, and whether to be treated again. The influence of rain intensity and interval time on the efficacy was determined, which provide a theoretical basis for the use of mesotrione.

Special thanks to you for your good comments, these are very helpful to our article.

**Reviewer #2:**

**1. Response to comment:** Line no. 14 space

**Response:** We are very sorry for our negligence, we have made correction according to the Reviewer’s comments.

**2. Response to comment:** Kindly mention control efficiency reduced how much percentage?

**Response:** The percentage of control efficiency reduction has been supplemented in the Summary and Conclusion. “At 6 mm/h within 1 h after treatment, the control effect of fresh weight was significantly reduced by more than 7.14%, and at 10 mm/h within 2 h, the control effect was significantly reduced by more than 14.78%”. Please review. Thanks so much for your useful comments.

**3. Response to comment:** Line no. 37 rewrite the sentence.

**Response:** As for the reviewer’s concern, we have re-written this sentence in line 57-58. The new sentence is “Weed would reduce the yield and quality of the crops, hinder mechanized sowing and harvest and then slow down the process of agricultural modernization”

**4. Response to comment:** Give at-least one example how much yield loss? What is the importance of *Chenopodium* weed, its biology, and morphological behavior could better explain the results.

**Response:** We are very sorry for our unclear report in this manuscript, we have made additions and changes in the Introduction, please review.

**5.** **Response to comment:** Mention its chemical content and concentration

**Response:** We choose 10% mesotrione emulsifiable concentrate (EC) as our herbicide, we added this part in Materials and methods .

**6. Response to comment:** Line no. 73 materials and methods the number may be given 2

**Response:** We have made correction according to the Reviewer’s comments, the full text has been modified in this way.

**7.** **Response to comment:** How you controlled the rainfall intensity, duration under field condition. Also drift effect is common under field condition.

**Response:** Because the weather is changeable in summer, so the different intensity of the short interval rainfall like light, moderate, heavy rainfall will all appear. According to the standard of China Meteorological Administration and the rainfall circumstance of TaiGu, we simulated the rainfall intensities were light rain 2 mm/h, moderate rain 6 mm/h and heavy rain 10 mm/h. Before the test in the field, we conducted many experiments about rainfall simulation. Firstly, we screened out one type of shower that sprayed the smallest water and most homogeneous when spraying. We also controlled the moving speed of spray lance by ourselves and found it is equal to the rainfall whose intensity is 2、6、10 mm/h when the capable of spraying was 4, 12 and 20 L per half an hour. According to the means and data of the preliminary experiment, we chose a windless and sunny day in the field experiment, and we put many baffles around the rainfall simulation areas of the neighborhood like the preliminary experiment. The simple facilities that put up by us when we test the rainfall intensity are as follows.

**8. Response to comment:** The experiment was conducted for one season only (4 months), whereas repeated results can explain the cause and effects better.

**Response:** The experiment was mainly conducted in the field, and we tested it again in the greenhouse. Ultimately, the conclusion we drew in the field and the greenhouse were almost consistent. These are some of the photos from our greenhouse experiments. The following figures show the influence of different intervals under the rain intensity of 2, 6 and 10 mm/h, with 0.5 h, 1h, 2 h and 4 h intervals from top to bottom.

**9．Response to comment:** Line 86: manual or power operated sprayer

**Response:** We choose power operated sprayer in our experience.

**10．Response to comment:**	Line 92: How artificial spraying mimicked the natural rain, what is the height of spraying on the weed. If you have picture just present for clear understanding.

**Response:** Before the start of the field test, we conduct an experiment for two months when the intensity and the precipitation time were suitable. The concrete operations are as follows： when spraying and mark the appropriate place in the electric spray knob by many tests, screening out one type of shower that sprayed the smallest water and most homogeneous, and we moved the spray lance in a constant speed within the unit area. The electric sprayer was continuously adjusted to the appropriate site according to the water yield, so that the water yield of 0.5 h was 4 L, 12 L and 20 L, reaching the rain intensity standard of 2, 6 and 10mm/h. The error is maintained at ±0.2 mm/h. The height of the spray is 0.5 meters, mark the wall in advance, such as the position of the green line in the last picture. In the newly submitted manuscript, we have rewritten the Materials and methods.

**11．Response to comment:** Only *Chenopodium* plans were measured? What about remaining weed species?

**Response:** *Chenopodium* is easy to survive under any bad existent conditions. It competes with crops on the water and nutrition, so the yield and the quality of crops sharply drop to a low level. And then the agriculture suffers massive loss. The damage that is caused by *Chenopodium* interference has been well documented in field crops. *Chenopodium* has been reported to reduce the barley (*Hordeum vulgare* L.) yield by 36%^[1]^. In sugarbeet (*Beta vulgaris* L.) field, the yield was reduced by 48%, which was associated with interference of this weed^[2]^. The presence of *Chenopodium* in corn fields has been shown to reduce the yield by 58%, and the yield of soybean will drop by 61% in the severe cases^[3]^. *Chenopodium* are always companied by the mixed agriculture pattern of millet (both foxtail millet and broomcorn millet), rice and wheat^[4]^. The experiment was carried out in foxtail millet field, *Chenopodium* is one of major weeds, so we did this experiment that focused on the *Chenopodium*. Of course, other weeds can also be targeted for future research, we will continue our study, thanks for your advice!

**Reference:**

[1] Conn JS, Thomas DL. Common lambsquarters (*Chenopodium album*) interference in spring barley. Weed Technology. 1987(1): 312–313.

[2] Schweizer EE. Common lambsquarters (*Chenopodium album*) interference in sugarbeets (Beta vulgaris). Weed Science. 1983; 31(1):5 – 7.

[3] Conley SP, Stoltenberg DE, Boerboom CM, Binning LK. Predicting soybean yield loss in giant foxtail (*Setaria faberi*)a n d common lambsquarters (*Chenopodium album*) communities. Weed Science. 2003; 51(3): 402–407.

[4] Gao YY. Research on *Chenopodium* in ancient southwest China. IOP Conference Series Earth and Environmental Science, 2021; 621: 012124.

**12．Response to comment:** What is the plot size?

**Response:** As the article says, the plot size is 4 m^2^ (2 m × 2 m). Here are some pictures of our experiment.

**Response:** I have carefully modified every figure according to the comments of the reviewer.

**13．Response to comment:** Study conducted in open field or green house conditions?

**Response**: This study was conducted in open field, but we did a confirmatory experiment in greenhouse.

**14．Response to comment:** Line 136: weed may be removed

**Response**: As Reviewer suggested that we have changed this part.

**15.	Response to comment:** Line no. 137: mention disease like

**Response**: We agree with the reviewer and corrected accordingly, to make it more informative and read more smoothly.

**16.	Response to comment:** Line no. 203: 4^th^

**Response**: We are very sorry for the mistakes in this manuscript and inconvenience they caused in your reading. The manuscript has been thoroughly revised.

**17. Response to comment:** Results are clearly explained but, Herbicide effect was studied on weed under sole weed cultivation.

**Response**: *Chenopodium* is listed among the most common dicotyledonous weeds in the world and it’s widely distributed in many agricultural areas, which severely reduce the yield and decrease the quality of the crops^[1]^. Because *Chenopodium* is one of the main weeds in the foxtail millet field and it is flooding in the drought areas, so we choose *Chenopodium* as our primary research object^[2]^. In order to show that the efficacy of mesotrione to weeds under different intensity, we use *Chenopodium* as the only research object in the experiment. We can regard the other weeds as a research in the following experiment period.

**Reference:**

[1] Moghadam S H, Alebrahim M T, Tobeh A, *et al*. Redroot Pigweed (*Amaranthus retroflexus* L.) and Lamb's Quarters (*Chenopodium album* L.) Populations Exhibit a High Degree of Morphological and Biochemical Diversity. Frontiers in Plant Science, 2021, 12: 593037.

[2]Song Xie, Guo Haoxuan, LiuYanan, et al. Investigation and Analysis on Occurrence of Weeds in Spring Sowing Millet Fields in Jinzhong City, Shanxi Province. Journal of Weed Science, 2020, 38(2): 9-15.(in Chinese)

**18. Response to comment:** But the response of herbicide would be different in real (mixed) condition with crop plants). Hence combined plant stand would explain better.

**Response**: We understand that combined crop plant stand would better. However, my senior schoolmates who study in our laboratory, he did some research about how much mesotrione would be safe or harmful for the foxtail millet. He found that the mesotrione is suitable for the foxtail millet field under the dose about 75-300 g/hm^2^. Because the weather in summer is changeable and rainy days are common here, so we had a further experiment to study if the rainwash would have an effect on the mesotrione and what effect would have. Then we can hope to screen out a safe and effective herbicide. In the present study, we mainly focused on the rainwash of the mesotrione and regarded *Chenopodium* as an assistant research object. We think that study under sole weed may not be optimal, but should be sufficient to draw my conclusion about the effect of rainfall intensity on mesotrione. As for the influence in the mixed condition, we will make further in-depth study, many thanks for your kind help!

**19. Response to comment:** No data on *Chenopodium* leaf area, since LA is the most important factor decides the efficacy of herbicide.

**Response**: Thank you for your instructive suggestions. We are sorry for not describing the data on *Chenopodium* leaf area in our manuscript. All leaf area were conducted statistical analyses by Image J, we found that the leaf area of the treatment was between 26.7 to 39.4 cm^2^.

**20. Response to comment:** What is the retention of herbicide concentration on weed leaves?

**Response**: I'm very apologetic for I didn't mention the residual quantity of the herbicide in my article, but I really agree with the reviewer's suggestions about my article. The retention of herbicide concentration on weed leaves will have an impact on the efficacy of mesotrione straightly. This experiment mainly studied the influence of rainfall intensity and time interval on the effect of mesotrione on *Chenopodium*. We are concentrating on the control effect and the physiological indexes of *Chenopodium*. This only is a pretty elementary research result, then we will conduct more studies about the leaves residual etc. Thanks for your valuable advice!

**21.** **Response to comment:** How much of applied herbicide was lost?

**Response**: I'm so sorry. We didn't measure the residual of the herbicide before or after the rain fall. But we calculate how much the efficacy of herbicide dropped by comparing the control effect of mesotrione on *Chenopodium* under different rainfall intensity. Because we chose plants that have similar size, the reduction of the plant height inhibition rate, the plant control effect, fresh weight control effect could show the loss of the mesotrione from the another side.

When the rain intensity was 2 mm/h, the plant height inhibition rate, plant control efficiency and fresh weight control efficiency of *Chenopodium* at intervals of 0.5 h and 4 h decreased by 18.07%, 18.12%, 16.25%, 4.86%, 1.50% and 2.01% respectively at 15 DAT. So we can infer that the loss of the herbicide is between 1.50% and 18.12%. In the same way, the weed control loss of the herbiside between 16.02% and 48.25%. when the rainfall intensity is 6 mm/h. And the loss is between 17.38% and 52.82% when the rainfall intensity is 10 mm/h, and we have added these datas in the new manuscript. As for the detail about herbicide loss, we will make a further study. Thanks for your meaningful comments!

**22.** **Response to comment:** Discussion is fine with the supporting literature. However, your results could have discussed more, for example time of rain interval, all rain intensities and interaction effect, what extent it affected.

**Response**: Your comments were highly insightful and enabled us to greatly improve the quality of our manuscript. We have re-written Discussion according to the Reviewer’s suggestion. We hope that the correction will meet with approval, thank you very much for your comments and suggestions.

**23. Response to comment:** Discussion section is lacking the supporting data on herbicide retention on leaves, leaching loss, biomass reduction.

**Response**: I agree with you ! Herbicide retention on leaves, leaching loss, biomass reduction are important datas what could show the efficacy of the mesotrione straightly. We only explore the control effect of mesotrione and the physiological indexes on *Chenopodium* under different rainfall intensity, this is a pretty basical research. We have konwn the loss and the residual of the herbiside are closely related to the efficacy. Hence, our group is going to carry out further experiments.

In addition to the above commernts, there is a comment regarding data in Table 3.

**1. Response to comment:** I think treatments comparison was done for T1 to T4 within the rainfall intensities. Whereas, comparison between all the treatment combinations (3*5= 15) would be good.

**Response:** I also think comparison between all the treatment combinations (3*5= 15) is a good idea. But in our experiment, we could intuitively observe that the efficacy loss of the mesotrione will be serious when the rainfall intensity was large. And the emphasis of this experiment was comparing the effect of rainfall interval to the efficacy of mesotrione under different rainfall intensity. Hence in order to draw a nice conclusion, we compared them separately.

We tried our best to improve the manuscript and made some changes in the manuscript. These changes will not influence the content and framework of the paper. And here we did not list the changes but marked in red in revised paper. We appreciate for Reviewers’ warm work earnestly and hope that the correction will meet with approval. Once again thank you very much for your comments and suggestions.

In all, I found the reviewer’s comments are quite helpful, and I revised my paper point-by-point. Thank you and the review again for your help.
