# Supplementary material for: Effects of mesotrione on the control efficiency and chlorophyll fluorescence parameters of Chenopodium album under simulated rainfall conditions
Source: PLoS One. 2022 Jun 3;17(6):e0267649. doi: 10.1371/journal.pone.0267649 (PMC9165882; doi:10.1371/journal.pone.0267649)
Supplement: Attachment — Submitted filename: Response to Reviewers.docx [file pone.0267649.s013.docx]

Dear Editors and Reviewers:

Thank you very much for your careful review on our manuscript and giving us the opportunity to resubmit our manuscript. Here we are submitting our manuscript that we have revised extensively according to your and reviewers` instructions and comments. We hope you agree that our revision has satisfactorily improved our manuscript for publication in *PLOS ONE*.

Here are our responses and corrections in the paper:

**1. Reviewers' comments:** Line no. 30、36, add space

**Responses to Questions:** Thank you for pointing this out, we have changed it.

**2. Reviewers' comments:** Line no. 64-66, Make the botonical name italic

**Responses to Questions:** We are very sorry for our negligence, we have made correction according to the Reviewer’s comments.

**3. Reviewers' comments:** Line no. 112——Table 1, check the min and max. temperatures

**Responses to Questions:** Our meteorological data was from the Taigu County Meteorological Bureau. To check the min and max temperature, we applied it to Jinzhong Meteorological Bureau again for data comparison, and the result is consistent. (Note: Taigu is a county of Jinzhong City)

**4. Reviewers' comments:** Line no. 114——Table 2, add space

**Responses to Questions:** Thank you for pointing this out, we have changed it.

**5. Reviewers' comments:** Line no. 118, CRD for field condition? Plz. check its CRD or randomized block design.

**Responses to Questions:** We are very sorry for our careless in the manuscript, we have corrected CRD to randomized block design in our revised manuscript.

**6. Reviewers' comments:** Line no. 120, mention the recommendation in terms of active ingredient (a.i.). The given dose may vary.

Responses to Questions: As for the reviewer’s concern, we checked the label of mesotrione again. We have changed line 120-122 as followed: “Mesotrione (10%, OD) was provided by Shandong Shengbanglvye Chemical Limited Company, the recommended effective dose given by the manufacturer was 150 g ai ha^-1^. The application was performed with a laboratory pot sprayer to deliver 450 L ha^-1^. ”

**7. Reviewers' comments:** Line no. 128-129, 1 L rainfall per 1 m^2^ = 1 mm Likewise, 1 L rainfall per 0.5 m^2^ = 2mm Why half an hour is mentioned here? clarify is is half an hour or half meter square.

**Responses to Questions:** I'm sorry I didn't write it clearly enough, “1 L rainfall per 1 m^2^ = 1 mm”, this is not entirely true, because it was limited by the time——one hour. It means that we have to make sure it lasted one hour when the 1 L rainfall fell on the ground whose area is 1 m^2^. We simulated light rain 2 mm/h in each experimental plot featured a dimension of 2 m × 2 m, it was that 8 L/h =2mm/h. If our spraying time is half an hour, it is equal to the rainfall whose intensity is 2 mm/h when the capacity of spraying was 4 L. In order to make it more clear for the audience, we have added the experimental plot size on the line 131 of the revised manuscript.

**8. Reviewers' comments:** Line no. 142、154、192、252, add space

**Responses to Questions:** Thank you for pointing this out, we have changed it.

**9. Reviewers' comments:** Line no. 598——Figures, Try to include clear figures as they are blurring after zooming

**Responses to Questions:** Thank you very much for this excellent suggestion, we have resumitted some pictures which are more clearer.

In all, I found the reviewer’s comments are quite helpful, and I revised my paper point-by-point. Thank you and the review again for your help.
